# Ischemic tolerance and cardiac repair in the spiny mouse (*Acomys*)

**DOI:** 10.1038/s41536-021-00188-2

**Published:** 2021-11-17

**Authors:** Tim Koopmans, Henriette van Beijnum, Elke F. Roovers, Antonio Tomasso, Divyanshu Malhotra, Jochem Boeter, Olympia E. Psathaki, Danielle Versteeg, Eva van Rooij, Kerstin Bartscherer

**Affiliations:** 1 Hubrecht Institute for Developmental Biology and Stem Cell Research, Royal Netherlands Academy of Arts and Sciences, Utrecht, The Netherlands; 2 Department of Biology and Center for Cellular Nanoanalytics (CellNanOs), Osnabrück University, Osnabrück, Germany; 3 Department of Cardiology, University Medical Center Utrecht, Utrecht, The Netherlands

**Keywords:** Mechanisms of disease, Cardiac regeneration

## Abstract

Ischemic heart disease and by extension myocardial infarction is the primary cause of death worldwide, warranting regenerative therapies to restore heart function. Current models of natural heart regeneration are restricted in that they are not of adult mammalian origin, precluding the study of class-specific traits that have emerged throughout evolution, and reducing translatability of research findings to humans. Here, we present the spiny mouse (*Acomys spp*.), a murid rodent that exhibits bona fide regeneration of the back skin and ear pinna, as a model to study heart repair. By comparing them to ordinary mice (*Mus musculus*), we show that the acute injury response in spiny mice is similar, but with an associated tolerance to infarction through superior survivability, improved ventricular conduction, and near-absence of pathological remodeling. Critically, spiny mice display increased vascularization, altered scar organization, and a more immature phenotype of cardiomyocytes, with a corresponding improvement in heart function. These findings present new avenues for mammalian heart research by leveraging unique tissue properties of the spiny mouse.

## Introduction

Heart failure affects more than 26 million people worldwide^1^, making effective therapies to treat cardiac disease a major public health goal. A key underlying cause of heart failure is the inability of the adult human myocardium to regenerate following injury combined with progressive deterioration known as pathological remodeling. Correspondingly, a central focus in cardiac regenerative research has been the restoration of heart lineages, including vascular cells, support cells, and most notably, cardiomyocytes. However, its effectiveness has been limited by the absence of naturally regenerating adult mammalian organisms^2^, restricting research to either non-mammalian vertebrates (such as zebrafish^3,4^) or neonatal mice, which are capable of scar-free cardiac repair up to 1 week after birth^5^. The spiny mouse (*Acomys spp*.) has emerged as a new model system, primarily due to its ability to scarlessly regenerate full thickness excisional wounds in the back skin and ear pinna^6,7^. However, whether regenerative capacity extends towards the essential internal organs is incompletely understood^8^. Here, we compared the response to ischemic injury following ligation of the left anterior descending (LAD) coronary artery (resulting in myocardial infarction (MI)), in *Mus musculus* (C57BL/6N, hereafter referred to as B6) and *Acomys cahirinus* (hereafter referred to as Ac), for whom coronary artery anatomy is largely similar^9^. We report that, despite an initial scar forming response and corresponding drop in heart function that is similar between the species, spiny mice exhibit significant tolerance to infarction, demonstrated by superior survivability, retainment of ventricular conduction, and near-absence of pathological remodeling. Importantly, *Acomys* hearts display a partial recovery in contractile output, a phenomenon associated with increased vascularization and altered scar organization.

## Results

### Increased tolerance and partial recovery following myocardial infarction in spiny mice

**Fig. 1:**
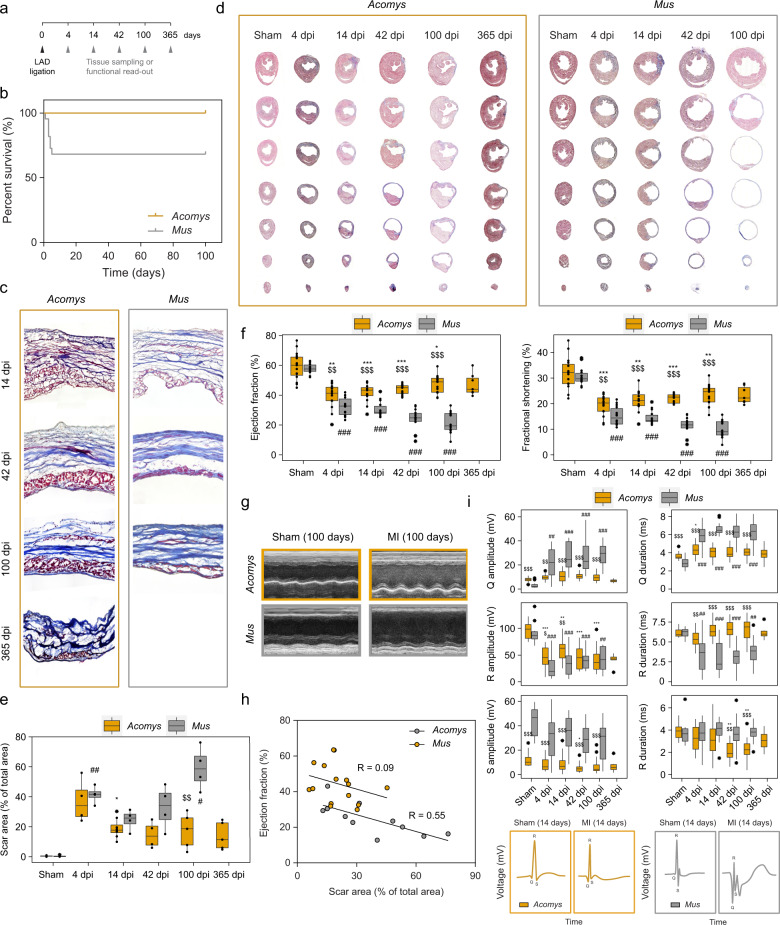
*Acomys* exhibit ischemic tolerance and partially recover heart function despite scar formation. **a** Overview showing the different time points after LAD ligation surgery, used for functional measurements or tissue sampling. **b** Kaplan−Meier survival curve for adult *Acomys* and *Mus* after LAD ligation, up to 100 days dpi. *n* = 12 per group. **c** Representative close-up images of a Masson’s Trichrome stain showing the collagenous scar area (blue) after different time points. **d** Representative images of infarcted hearts showing transversal progression from point of ligation down to the apex. **e** Scar area (% of total area) development over time, based on transversal stacks stained with Masson’s Trichrome (see ‘Methods’). **f** Echocardiographic quantification of cardiac output after MI or sham-control, including ejection fraction (volumetric percentage of fluid ejected from the chamber with each contraction) and fractional shortening (percentage of diastolic dimension that is lost in systole). Graph contains mostly animals that were only measured at one time point (unpaired samples), and some that were measured over different time points (paired samples). **g** Corresponding representative images of the M-mode recording shown in (**f**). **h** Correlation plot from all animals that had their ejection fraction and corresponding scar area measured. **i** Electrocardiogram quantification (see ‘Methods’) showing QRS wave parameters (amplitude and duration) after MI or sham-control, with corresponding representative illustration of the QRS wave. Graph contains animals that were measured over different time points (paired samples), as well as animals that were only measured at one time point (unpaired samples). *n* = 15 for *Acomys* (*n* = 7 for 365 days), *n* = 14 for *Mus*. **e**, **f**, **i**: Two-way mixed ANOVA followed by Bonferroni post hoc test. For all comparisons: * is significant compared to *Acomys*-sham, # compared to *Mus*-sham, $ is significant in an inter-species comparison of the same time point (e.g. 100 days to 100 days). * is *p* < 0.05, ** is *p* < 0.01, and *** is *p* < 0.001. Box plots represent the median, interquartile range (IQR), minimum (25th percentile – 1.5 × IQR), and maximum (75th percentile – 1.5 × IQR).

Before the assessment of injury parameters, we measured basic metrics concerning heart function and morphology. Although B6 animals had a lower total body weight and heart rate than Ac (24 ± 0.3 g vs 32 ± 0.6 g and 402 ± 6 bpm vs 488 ± 27 bpm respectively), we observed similar weights and morphology for healthy hearts (122 ± 2.8 mg compared to 130 ± 4.3 mg) (Supplementary Fig. 1a, b). To capture the dynamics of cardiac ischemia and potential repair following LAD ligation, we analyzed heart function and tissue samples at different time points (days post-injury, dpi) up to 1 year after initial injury (Fig. 1a). Although B6 took on body weight more rapidly than Ac, we found that the MI procedure had no add-on effect on body weight compared to sham-controls, for both species (Supplementary Fig. 1c). Because of potential differences in tibia length in Ac, we proceeded to use body weight as a reference point for our subsequent analyses where appropriate. Remarkably, Ac was extremely tolerant towards the surgical procedure and all animals survived, contrary to B6 who exhibited 37.5% lethality (Fig. 1b). Yet, Ac developed scar tissue throughout the damaged area, which appeared grossly similar to that of B6 (Fig. 1c). We proceeded to accurately quantify scar area using a series of transversal stacks, from the point of ligation down to the apex (see ‘Methods’ for details). Both species developed an initial scar that was similar in size; however, scar size started diverging thereafter. While scar size in Ac decreased over time until eventually plateauing 42 dpi, B6 developed a large scar that continued to expand due to compensatory hypertrophy and progressive dilatation (Fig. 1d, e, see also Extended Table 1 for an overview of all statistical results). In accordance with an increased scar area in B6, echocardiography revealed a continued worsening of ejection fraction (from 32.1 ± 1.6% at 4 dpi to 21.5 ± 1.9% at 100 dpi) and fractional shortening in B6, whereas Ac continuously improved over a 100-day period post-MI (from 39.9 ± 2.2% at 4 dpi to 47.0 ± 1.7% at 100 dpi) (Fig. 1f, g), but started plateauing thereafter. Importantly, scar area negatively correlated with ejection fraction in B6, whereas Ac showed a much poorer correlation (Fig. 1h). These results suggest scar tissue in Ac may differ from conventional mammalian scars, with functional properties that permit cardiac output. Accordingly, we measured electrical conductance of the heart using electrocardiography (ECG), and measured quantitative differences in QRS behavior that serves as a critical indicator of left ventricular conductivity. Most notably, after MI, we found a strong and enduring increase in both Q amplitude and duration for B6, resembling pathological Q waves in human patients^10^, with no significant alterations for Ac (Fig. 1i). To substantiate our findings, we measured functional heart parameters using a reperfusion injury model, whereby we temporary ligated the LAD for 1 h, after which arterial blood flow was restored (Supplementary Fig. 2a). With regards to scar size (Supplementary Fig. 2b, c, d), echocardiography (Supplementary Fig. 2e, f), and ECG (Supplementary Fig. 2g) we observed similar differences between Ac and B6 compared to the MI model, albeit in a milder form. Collectively, our findings demonstrate that Ac exhibits ischemic tolerance in spite of significant scar deposition and reduced heart function, followed by partial recovery of cardiac output over time.

### Near-absence of pathological remodeling in spiny mice

**Fig. 2:**
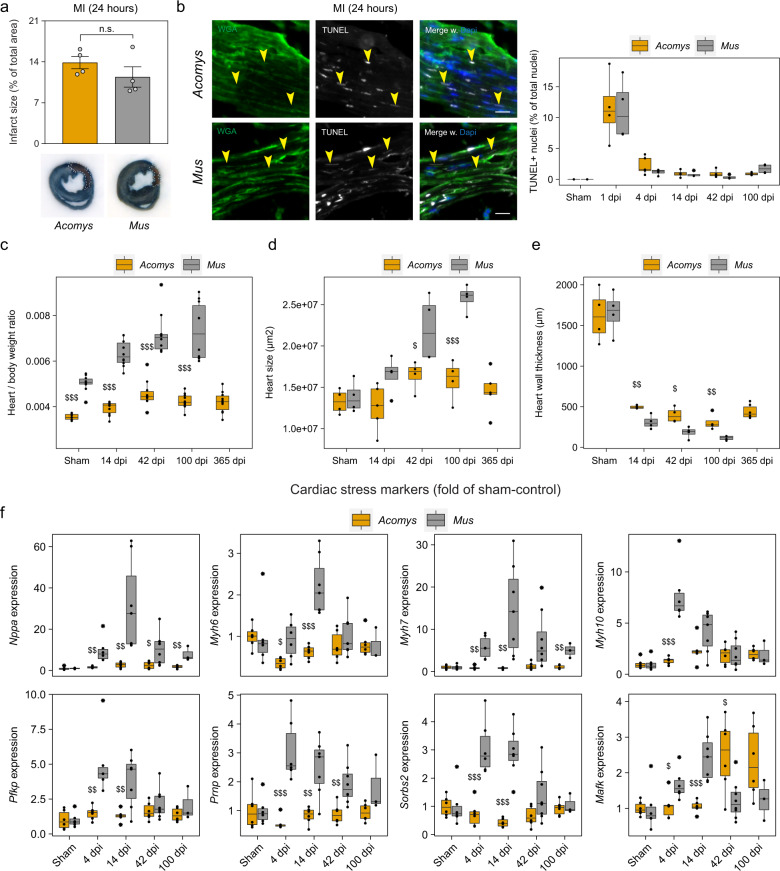
Near-absence of infarction-induced remodeling in *Acomys*. **a** Infarct size of mice injected with Evans blue 24 h after surgery. Unpaired *t* test. **b** TUNEL-reactive dying cells (yellow arrows) in the scar area of infarcted hearts. Scale bar = 50 µm. **c** Heart to body weight ratio progression over time. **d** Heart size progression, based on transversal stacks stained with Masson’s Trichrome (see ‘Methods’). **e** Heart wall thickness derived from five points-of-measurements along the scar wall. **f** Relative gene expression from whole heart ventricle homogenates subjected to RT-qPCR. Samples have been normalized to the sham-control (*Acomys* or *Mus*). **b**−**f** Two-way mixed ANOVA followed by Bonferroni post hoc test. For all comparisons: * is significant compared to *Acomys*-sham, # compared to *Mus*-sham, $ is significant in an inter-species comparison of the same time point (e.g. 100 days to 100 days). * is *p* < 0.05, ** is *p* < 0.01, and *** is *p* < 0.001. Box plots represent the median, interquartile range (IQR), minimum (25th percentile – 1.5 × IQR), and maximum (75th percentile – 1.5 × IQR). All bar graphs represent the mean ± standard error of the mean (SEM).

To analyze the acute response to ischemia and test if both species are initially similarly affected by the surgery, we injected mice with Evans blue dye intravascularly 24 h after surgery, and quantified the infarct zone. We additionally quantified the number of TUNEL-reactive cells in the scar area post-MI as a proxy for cell death and used a wheat germ agglutinin (WGA) conjugate to label the scar^11^. In both cases there were no statistical differences between the groups (Fig. 2a, b). We next assessed differences in heart and body weight as a read-out for pathological remodeling (clinically measurable changes that affect heart size, shape or function^12^). We observed a stark and continued increase for B6, with only a mild increase for Ac (Fig. 2c). Correspondingly, heart size (calculated as the mean surface area from six equally spaced transversal stacks) and heart wall thickness continued to deteriorate for B6, but plateaued for Ac 42 dpi (Fig. 2d, e). In addition, mRNA levels derived from whole ventricles marking known targets for heart failure or cardiac hypertrophy^13^, including members of the myosin heavy chain and natriuretic peptide family, confirmed these findings, showing a dramatic MI-induced increase in B6, but virtual absence in Ac (Fig. 2f). We conclude that although Ac animals suffer from an initial damage response that is similar to B6, animals are largely protected from pathological remodeling that develops thereafter.

### Altered scar organization in spiny mice

**Fig. 3:**
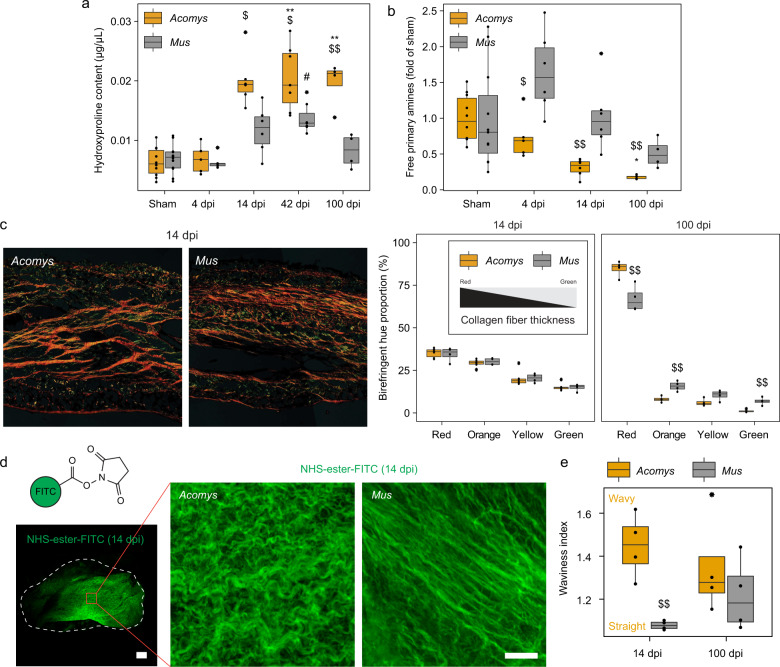
Scar organization is different in *Acomys* and *Mus* hearts. **a** Hydroxyproline assay from whole heart ventricle homogenates. **b** TNBSA assay from whole heart ventricle homogenates, normalized to total collagen content (hydroxyproline assay) and sham-control. **c** Polarized light imaging of injured hearts to analyze collagen fiber thickness. Left panel are representative images of the scar center (14 dpi) with the corresponding quantification on the right. **d** N-hydroxysuccinimide ester labeling and whole-mount imaging of injured hearts (14 dpi). Scale bar = 200 µm (overview) and 50 µm (inlet). **e** Waviness of individual collagen fibers, quantified from (**d**). **a**, **b**, **c**, **e** Two-way mixed ANOVA followed by Bonferroni post hoc test. For all comparisons: * is significant compared to *Acomys*-sham, # compared to *Mus*-sham, $ is significant in an inter-species comparison of the same time point (e.g. 100 days to 100 days). * is *p* < 0.05, ** is *p* < 0.01, and *** is *p* < 0.001. Box plots represent the median, interquartile range (IQR), minimum (25th percentile – 1.5 × IQR), and maximum (75th percentile – 1.5 × IQR).

Our electrocardiography findings (Fig. 1i) suggest altered scar properties that may facilitate regenerative processes in Ac. Accordingly, we measured total collagen content of sham-operated and injured heart ventricles using hydroxyproline as a read-out for collagen abundance. Unlike our findings related to scar size, Ac scars were characterized by increased collagen content compared to B6, maintaining high levels up to 100 dpi (Fig. 3a). In line with this, relative Platelet-derived growth factor receptor beta (PDGFRβ)+ fibroblast coverage within the scar developed similarly with hydroxyproline levels, with increased levels for Ac especially during late-stage conditions (Supplementary Fig. 3a). Consistent with increased collagen production in Ac and a thicker heart wall (Fig. 2e), we observed enhanced collagen cross-linking over time compared to B6 scars (Fig. 3b). To confirm these findings, we performed polarized light microscopy on heart sections, which revealed an increased presence of large matured fibers in late-stage scars that can be captured in the red color hue range^14^, at the cost of smaller less matured fibers (orange to green hue range) (Fig. 3c) for both species (14 vs 100 dpi). However, while early scars showed no difference, Ac late scars contained more matured fibers than B6 scars (Fig. 3f) in line with our earlier observations. Because increased collagen load and cross-linking is associated with diastolic dysfunction and cardiac remodeling^15,16^, we reasoned that the ECM architecture must be altered in Ac to permit partial heart function recovery in spite of increased collageneic pressure. We therefore labeled the scar surface with an N-hydroxysuccinimide ester compound, an amine-reactive crosslinker conjugated to a green fluorescent fluorophore (Fig. 3d). Due to the presence of free amine groups in collagen, we were able to perform a highly selective labeling of the surface matrix using these ester compounds, without labeling any cellular structures (Fig. 3d). Consistent with our earlier findings, Ac scars were loaded with thick ECM fibers positioned in a basket-weave pattern that is generally associated with favorable regeneration outcomes in dermal compartments^17^. Contrary, B6 scars were much more heterogeneous within the scope of the entire scar, and at numerous locations presented with thinner straight fibers that appeared more aligned to one another. To confirm these observations, we analyzed four main ECM parameters using Euclidean and non-Euclidean geometry: (1) the fractal dimension representing the complexity and scalability of the scar; (2) lacunarity representing the gappiness and inhomogeneity of the scar; (3) anisotropy representing the directional homogeneity of adjacent fibers; and (4) waviness representing the straightness of individual fibers. Interestingly, we found that the overall complexity of the scar was similar between the species, but differed for the individual fiber arrangements. Most prominently, Ac fibers were more wavy during early scar conditions (Fig. 3d, e). In accordance, the Ac fiber network appeared less aligned as indicated by lower anisotropy values, although this did not reach statistical significance (Supplementary Fig. 3b). Overall, we conclude that Ac scars, although smaller in size compared to B6, present with a higher collagen load and accompanying maturation profile, but with greater recoil potential that may facilitate heart function during systole and permit regenerative processes.

### Cardiomyocyte maturation profile altered in spiny mice

**Fig. 4:**
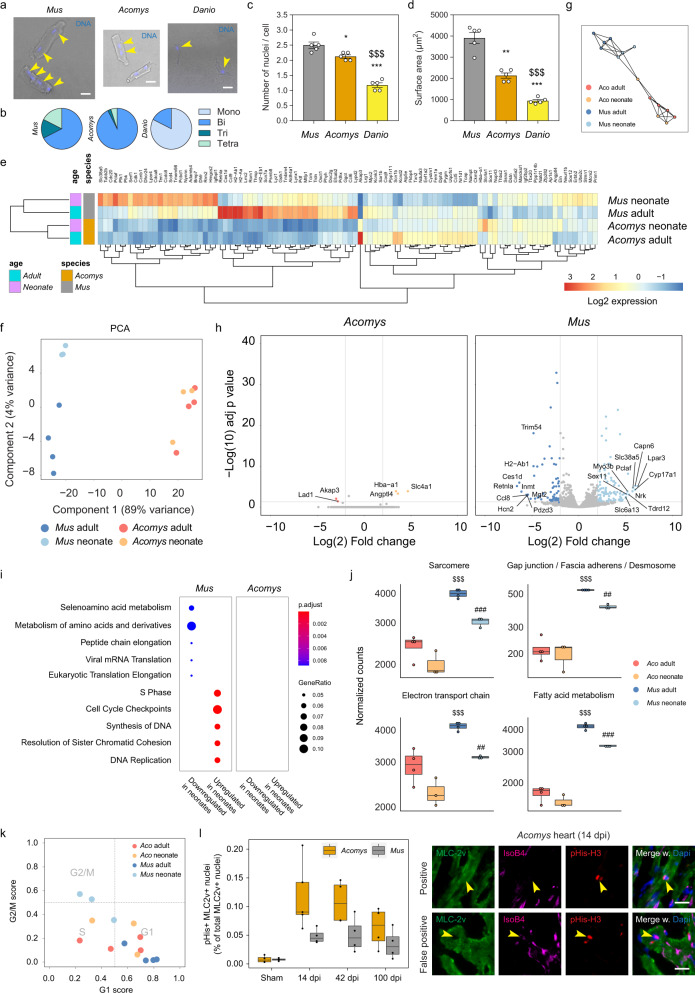
Cardiomyocytes in *Acomys* hearts display an immature phenotype. **a** Representative images of isolated cardiomyocytes (CM) using potassium hydroxide (see ‘Methods’), counterstained with Hoechst (yellow arrows). Scale bar = 30 µm. **b** Pie chart showing the mean percentage of CM nucleation per species. **c** Mean nuclei number and **d** corresponding surface area of isolated CMs. One-way ANOVA followed by Tukey’s post hoc test. **e** Heatmap from healthy bulk-sequenced heart ventricles, showing the top 100 most significant differentially expressed genes between the samples. **f** Sample-to-sample distance visualization through principal component analysis (PCA). **g** Pairwise correlation network reveals a maturation topology. Correlation threshold, Pearson’s *r* > 0.4. **h** Volcano plot of top regulated genes (after thresholding) in *Acomys* (adult vs neonate) and *Mus* (adult vs neonate). **i** Over- or underrepresented enrichment terms (Reactome database) in *Acomys* (neonate) and *Mus* (neonate). **j** Cumulative expression score of genes that fall under different ‘Cellular components’ GO terms, e.g. the sarcomere (GO:0030017). **k** Assignment of cell cycle stage for each sample (see ‘Methods’). **l** CM (marked by MLC-2v) proliferation that are isolectin B4 negative (to exclude capillaries) in heart ventricles, indicated by phosphorylated Histone H3 (ser 28). Panels at the right side indicate representative fluorescent antibody or dye images showing proliferating (positive and false positive) CMs (yellow arrows). Scale bar = 20 µm. **j**, **l** Two-way mixed ANOVA followed by Bonferroni post hoc test. For all comparisons: * is significant compared to *Acomys*-sham, # compared to *Mus*-sham, $ is significant in an inter-species comparison of the same time point (e.g. 100 days to 100 days). * is *p* < 0.05, ** is *p* < 0.01, and *** is *p* < 0.001. Box plots represent the median, interquartile range (IQR), minimum (25th percentile – 1.5 × IQR), and maximum (75th percentile – 1.5 × IQR). All bar graphs represent the mean ± standard error of the mean (SEM).

To assess a putative capability for cardiac regeneration in spiny mice we first addressed global proliferation dynamics using 5-ethynyl-2ʹ-deoxyuridine (EdU) (Supplementary Fig. 4a) and subsequent confocal immunofluorescence (IF) imaging. Ac and B6 hearts showed similar proliferation dynamics and relative cell numbers within the scar area, with cycling cells present throughout the different scar areas (Supplementary Fig. 4b, c). As proliferation per se is not necessarily a measure of cardiac repair, we expanded our search towards specific lineages of the heart. Accordingly, we diverted our attention towards the cardiomyocyte (CM) as the central player of heart function. We first assessed nuclei numbers in isolated CMs (Fig. 4a) as an indication of regeneration potential^18^, and included zebrafish (*Danio rerio*), which have high regeneration potential and mainly mononucleated CMs, in our analysis to establish a ‘regeneration framework’. While *Danio* hearts presented with a single nucleus per cell in more than 80% of the cases (on average 1.18 ± 0.08 nuclei), we did not find any of these cells in B6 or Ac (Fig. 4b), even though these have been reported in refs. ^18,19^. The degree of tri- and tetra-nucleated CMs and accompanying cell surface area, however, was significantly higher in B6 compared to Ac (on average 2.5 ± 0.10 nuclei vs 2.1 ± 0.06 nuclei for B6 and Ac respectively, Fig. 4c, d). Based on these results we predicted that Ac CMs may exhibit a more immature phenotype than B6 CMs^19^. Correspondingly, we quantified the distance between sarcomeres as a proxy for CM maturation and observed a modest increase in spacing for Ac compared to B6 (Supplementary Fig. 5a). To further test this hypothesis, we performed bulk sequencing on whole ventricles on adult (7 weeks) and neonatal (p3) mice—which still have the potential to regenerate damaged heart tissue^5^—to establish maturity parameters of the healthy myocardium, compared between the species. We employed an additive model to compare the effects of age, while controlling for species, and found many differentially expressed genes (Fig. 4e). Interestingly, principal component analysis (PCA) showed a clear separation for the species with strong overlap for Ac adult and neonatal samples (Fig. 4f). To take out potentially masking effects from the B6 samples, Ac was additionally graphed separately, which demonstrated slight separation between adult and neonates (Supplementary Fig. 5b). Using genes from the PCA, we inferred lineage relationships among the samples in an adjacency network on the basis of pairwise correlations between samples^20^, which confirmed the species separation, with overlap within the Ac pool (Fig. 4g). Correspondingly, when adult hearts were individually compared to neonatal hearts, virtually no significantly changed genes above a 2.0 log_(2)_ fold-change threshold were found for Ac, whereas these differences were much more apparent in B6 (Fig. 4h). Equally, while we found enrichment terms associated with cell cycling and DNA synthesis for B6 neonates, no significant enrichment was found in Ac neonates (Fig. 4i). These results strengthen the hypothesis that adult Ac ventricles exist in a more immature state as compared to B6. To expand on these findings, we generated a cumulative score of genes associated with CM maturation, based on published GO terms (cellular components): (1) sarcomeric load (GO:0030017), (2) cellular integration (GO:0005921, GO:0005916, GO:0030057), (3) electron transport chain components (GO:0022900), and (4) and fatty acid metabolism (GO:0006631)^21^. Although B6 presented with lower sample variation likely due to its inbred nature, we consistently found elevated expression for members of these categories in B6 adults compared to B6 neonates, and an even lower expression for Ac regardless of age (Fig. 4j). To validate our RNAseq findings, we performed RT-qPCR on whole ventricles and probed for known maturation genes. In the mammalian heart, transition into adulthood is characterized by the inactivation of the fetal gene products Tnni1 and Myl7, in concert with its stoichiometric replacement by the adult products Tnni3 and Myl2, thus representing a quantifiable ratiometric maturation signature^22^. RT-qPCR confirmed our RNAseq data with regards to these genes, and showed an exaggerated ‘maturation’ response in B6 as compared to Ac, further supporting a more juvenile phenotype of the ventricular myocardium in Ac (Supplementary Fig. 5c).

Finally, to establish the potential proliferative capacity of CMs, we predicted cell cycle states based on expression patterns of pre-trained classifiers (see ‘Methods’)^23^. All B6 adult replicates existed in G1 phase, while B6 neonates mostly occupied G2/M. Interestingly, Ac adult and neonates displayed a shift from G1 into S phase (Fig. 4k). We next quantified all dividing Myosin light chain-2 (MLC-2v) positive nuclei in transversal sections that were isolectin B4 negative (to rule out false positives from the intermingled capillary network) (Fig. 4l). In line with their immature phenotype, the number of Ac CMs positive for EdU was higher compared to B6 at 14 days after MI (3.6 ± 0.5% vs 1.2 ± 0.07%) and borderline significant for phosphorylated Histone H3 (0.12 ± 0.03% vs 0.05 ± 0.007%) (Fig. 4l and Supplementary Fig. 5d). Overall, our data suggest a modestly increased proliferative potential of the ventricular myocardium to respond to ischemia in Ac, possibly due to the presence of less differentiated cardiomyocytes.

### Functional and sustained angiogenesis in scarred spiny mouse hearts

**Fig. 5:**
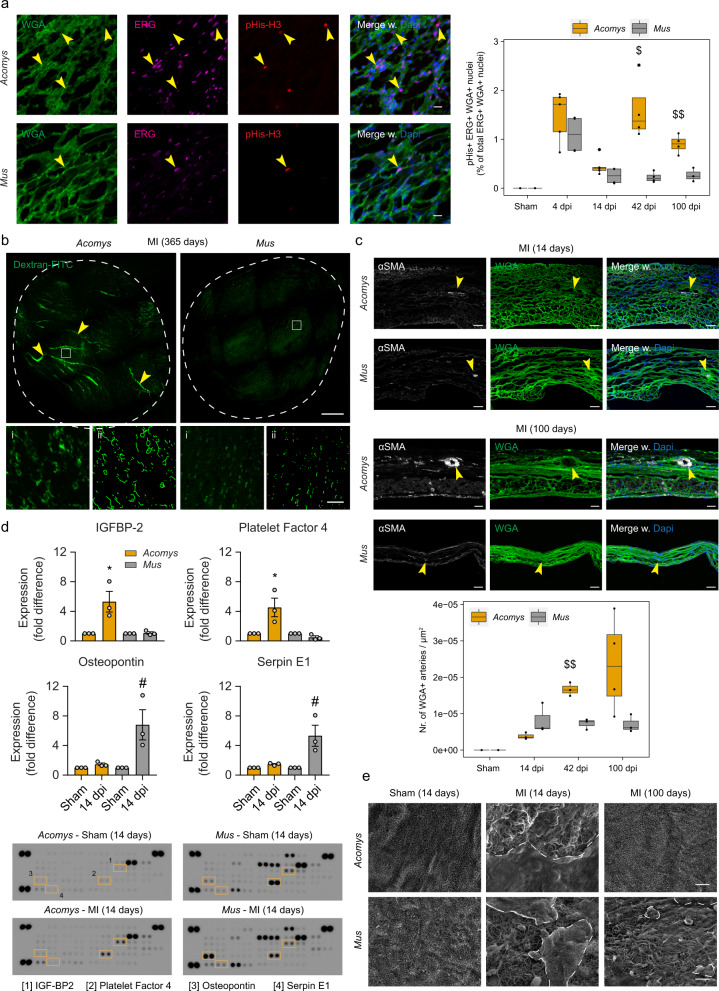
Vascular repair in injured *Acomys* hearts. **a** ERG-positive (yellow arrows) endothelial proliferation in the scar area of infarcted hearts, marked by phosphorylated Histone H3 (ser 28). Scale bar = 20 µm. **b** Whole-mount confocal images showing Dextran-FITC-perfused vessels inside 365-day-old infarcted hearts. Yellow arrows point towards large vessels. Scale bar = 500 µm (overview) or 50 µm (inlet). Inlets (i) were skeletonized with ImageJ to create binary images (ii) showing 2D branches and junctions of the capillary network. Experiment was repeated two times per conditions. **c** Number of muscularized arteries (>10 µm in diameter, yellow arrows) marked by α-SMA in the scar area of infarcted hearts. Scale bar = 100 µm. **d** Proteomic profiling of angiogenic proteins, from whole heart ventricle homogenates (14-day post-MI and 14-day sham-controls). Samples are normalized to the sham-control (*Acomys* or *Mus*). Graphs are derived from the blots pictured below, showing numbered squares for each indicated graph. Every dot inside the square represents one protein in duplicate. The array was repeated three times. **e** Scanning electron microscopy image of sham-operated and infarcted hearts showing the disrupted/regenerated epicardium. Scale bar = 10 µm. Experiment was repeated two times per condition. **a**, **c**, **d** Two-way mixed ANOVA followed by Bonferroni post hoc test. For all comparisons: * is significant compared to *Acomys*-sham, # compared to *Mus*-sham, $ is significant in an inter-species comparison of the same time point (e.g. 100 days to 100 days). * is *p* < 0.05, ** is *p* < 0.01, and *** is *p* < 0.001. Box plots represent the median, interquartile range (IQR), minimum (25th percentile – 1.5 × IQR), and maximum (75th percentile – 1.5 × IQR). All bar graphs represent the mean ± standard error of the mean (SEM).

We next addressed vascular dynamics and angiogenesis. Using the ETS transcription factor ERG and phosphorylated Histone H3 (serine 28) to mark dividing endothelial cells^24^, we observed an increased proliferative response during the early injury phase that was similar for both species (Fig. 5a). However, while the number of dividing cells rapidly declined after 4 days in B6, Ac exhibited a second wave of cell divisions that maintained high levels up to a 100 days post-MI (Fig. 5a). We confirmed these findings using isolectin B4 to label capillary vessels (Supplementary Fig. 6a, b). To analyze the angiogenic response further, we injected mice with Dextran-FITC intravascularly 1 year after surgery, and imaged the perfused scar using whole-mount confocal imaging. While both species showed a dense capillary network throughout the scar surface, Ac capillaries appeared more connected to one another (Fig. 5b). We additionally observed the presence of large arteries in Ac scars, which were completely absent in B6 scars (Fig. 5b). To confirm these findings and visualize muscularized arteries, we stained whole-mount scars or healthy myocardium for alpha-smooth muscle actin (α-SMA). While both species showed a strong regression in the number of arteries 14 days post-MI, Ac was able to regenerate the vascular bed, as demonstrated by a large and well-branched network of vessels in 100-day old scars (Supplementary Fig. 6c, d). Conversely, in line with our perfusion experiments, B6 scars 100 days post-MI appeared to have regressed even further. We proceeded to quantify the number of α-SMA+ vessels in the scar on tissue cryo-sections, which confirmed the gradual increase in artery coverage in Ac, with a more stagnated response for B6 (Fig. 5c). To explore the angiogenic program underlying these responses, we screened for the expression of 53 different angiogenic proteins in infarcted (14 days post-MI) and sham-operated whole ventricles using proteomic profiling. Corresponding to our earlier observations, we observed divergent angiogenic responses between the species (Fig. 5d and Supplementary Fig. 7a). More specifically, IGFBP-2 and Platelet factor 4 were uniquely expressed in injured Ac hearts, while B6 hearts were associated with increased expression of Osteopontin and Serpin E1 (Fig. 5d). We next investigated the epicardium, a mesothelium covering the surface of the heart. The epicardium is critical for coronary vasculature development during mammalian embryogenesis and during adult zebrafish heart regeneration^25^. Using scanning electron microscopy on scarred and sham-operated tissues, we imaged the epicardial surface across different time points post-MI. In sham-operated hearts the epicardium appeared continuous and riddled with microvilli that is characteristic of the healthy mesothelium^26^. Following injury, both species showed a disrupted epicardial layer 14 days post-MI, exposing the underlying extracellular matrix/basement membrane. Interestingly, the epicardium appeared completely restored in Ac hearts after 100 days, while B6 hearts showed no improvement (Fig. 5e). Immunofluorescence on tissue cryo-sections using the mesothelial marker DDAH2 (Dimethylarginine Dimethylaminohydrolase 2) confirmed these results (Supplementary Fig. 6e). In conclusion, we report an angiogenic program in Ac leading to the formation of capillaries and arteries that is maintained up to 1 year post-MI. By contrast, the formation of these vessels never reaches full maturity in B6, and is incapable of reconstructing the pre-injury state. Collectively, our findings demonstrate that *Acomys cahirinus* presents a novel example of ischemic tolerance and cardiac repair in the mammalian class and constitutes a new experimental paradigm for cardiac research.

## Discussion

Given the crushing medical complications of patients with end-stage heart failure, the marked global increase in prevalence, and the severe limitation of donor hearts for transplantation, heart regeneration research has been the most compelling case for translating fundamental advances into clinical practice. Yet, as of today, an applicable working therapy to human patients still awaits discovery, urging the need for new and promising avenues in regeneration research. The essence of regeneration biology comes from our current regime of cardiac models, including zebrafish, teleost fish, urodele amphibians, and neonatal mice and pigs^27^. While these models have significantly advanced our understanding of regenerative mechanisms, and in some cases have led to marked improvements in cardiomyocyte expansion in adult mice^28–31^, they impose restrictions in that they are either of non-mammalian vertebrate (e.g. zebrafish or newts), or neonatal (mice or pigs) origin. On the basis of our findings in this study, we propose a new mammalian model for adult cardiac repair—the spiny mouse.

Compared to adult C57BL/6 mice, we demonstrate superior survivability, with accompanying near-absence of ventricular remodeling. In human patients, ventricular remodeling still occurs in around 30% of human myocardial infarcts despite timely primary coronary intervention and optimal standard pharmacotherapy (e.g. angiotensin-converting enzyme inhibitors (ACE-I), β-blockers, etc.)^32^. Interestingly, our findings revealed that scar size area correlates poorly with ejection fraction, contrary to B6, which may suggest that Ac scars hold properties that can foster repair and function rather than pathological remodeling. Notably, ventricular conductance only showed mild alterations, with no significant changes in Q wave dynamics. In infarcted hearts of human patients, pathological Q waves are characteristic of necrotic tissue and represent the area of the myocardium that cannot be depolarized. Electrogram measurements of human infarcted hearts have indicated a degree of residual electrical activity within the scar, albeit with nonuniform conduction^33^, which suggests potential differences in the cellular and molecular composition of the scar may influence electrical conductance. In Ac, scar-inhabiting fibroblasts may exhibit increased cell-cell coupling, or respond differently to the stiffening environment of the scar, with altered traction forces generated by these cells with accompanying differences in extracellular matrix production^34^. Consistent with this notion, we found an altered ECM organization in Ac compared to B6. How Ac hearts maintain conductivity in the presence of scar tissue, and whether or not the scar itself allows electrical conductance warrants further investigation, and may offer a new and currently unexplored platform for prevention strategies towards ventricular remodeling and heart failure.

In addition, we show partial functional recovery in Ac, possibly owing to increased vascularization, altered scar organization, and cardiomyocyte expansion, with an associated improvement in heart function that is clinically significant^35^. Whether Ac can completely regenerate the heart, including the resolution of existing scar tissue and full reconstitution of the myocardium is improbable and likely not clinically meaningful, since we only report a modest improvement in CM proliferation compared to B6. Although we document a more immature CM phenotype in Ac, we suspect the predominant bi-nucleation of these cells impedes an efficient expansion during injury conditions. For example, the proliferative response of zebrafish CMs, which are predominantly mononucleated, can be blunted following artificial polyploidization of the myocardium^36^. In spite of these limitations, the mammalian background of Ac presents unique opportunities for cardiac research that permits the study of a more systems-based approach within the context of mammalian physiology for the first time. Although we used the C57BL/6 parental *Mus* strain for our comparative approach, which has one of the highest MI-induced mortality rates^19,37^, we report on critical aspects of cardiac repair that to our knowledge have not been described in other *Mus* strains. Consequently, we propose that *Acomys* in essence initiates a wound-healing response much like *Mus* aimed primarily to compensate for the reduced cardiac output and debilitating conditions induced by the scar. However, while *Mus* strains resort to compensatory and pathological hypertrophy, *Acomys* recruits a more directed vascular response, restoration of epicardial continuity, and undergoes changes that affect scar properties. These changes likely prevent or significantly reduce the need for hypertrophic mechanisms in *Acomys*, leading to superior survivability and better overall heart function.

How did these regenerative traits evolve? *Acomys* exhibits tail sheath autotomy and shows an unusually fragile attachment of the back skin to the underlying fascia that tears off easily^6^. Presumably, these properties allow the animal to break free from its predators grip once captured, but result in open-wounds that need to be healed. Wounds can be substantial, up to 60% of the total dorsal surface area^6^. Yet animals do not perish from an overwhelming infectious response, likely underlying an immune response favorable of regeneration^38^. Even though this and other regenerative traits may have developed due to local selective pressures on the skin, the systemic reach of the regenerative response may ‘bleed’ into an otherwise non-regenerative heart in the context of injury. Alternatively, regenerative traits in resident skin cells could have already been acquired during early mesodermal development that gives rise to heart tissue and dermal compartments of the skin, and may therefore be shared across organ-specific lineages in adult stages. Future studies into these and other cellular mechanisms will be crucial to help understand the regenerative response of spiny mice.

Together with the accompanying study by Peng et al.^9^ in this issue of npj Regenerative Medicine and a study that was published during our revisions^39^, our findings solidify the spiny mouse (*Acomys cahirinus*) as a novel mammalian model for adult cardiac repair. Its vascular reconstitution, altered scar organization, and protection from pathological remodeling provide new avenues for cardiac regenerative medicine and alternative viewpoints in an otherwise cardiomyocyte-centric field.

## Methods

### Animals

All animal experiments were conducted under strict governmental and European guidelines and were approved by the Animal Welfare Committee of the Royal Netherlands Academy of Arts and Sciences, under license number AVD80100 2018 7144. Adult pathogen-free male C57BL/6N mice (7–9 weeks old) were obtained from Charles River Laboratories and group-housed at a density of 2–4 individuals in static individually ventilated cages (IVC) (Tecniplast, #GM500) with corncob granules (Rehofix, #MK1500). They were given autoclaved water and high nutrient food chow (RM3 pellets, Special diet) ad libitum. Adult pathogen-free male spiny mice (*A. cahirinus*; 7–9 weeks old) were bred and maintained in-house. They were group-housed at a density of 2–8 individuals in static IVCs (Tecniplast, #GR1800DD) with powdered cellulose pellets (ARBOCEL) and given autoclaved water and low nutrient and low protein food chow (RM1 pellets, Special diet) ad libitum. On occasion, and always after surgery, they additionally received black-oil sunflower seeds and a combination of dried vegetables. All cages were kept in climate-controlled quarters with a 12 h/12 h light/dark cycle.

### LAD ligation surgery

Myocardial infarction, ischemia-reperfusion, or sham surgery was performed in adult mice through permanent or temporary ligation (60 min) of the left anterior descending artery (LAD). Mice were anesthetized by an intraperitoneal injection of a Fentanyl (50 μg/kg), Midazolam (5 mg/kg), and Dexmedetomidine (500 μg/kg) cocktail, hereafter referred to as FMD. Monitoring anesthetic depth was assessed by toe reflex. Animals were placed on a warming plate (39 °C) and eyes were covered with Bepanthen to avoid dehydration. The thorax was shaved and disinfected with betadine and sterile phosphate-buffered saline (PBS). A tracheal tube was placed and the mouse was connected to a ventilator (UNO Microventilator UMV-03, Uno BV.). Using aseptic technique with sterile instruments the skin was incised at the midline to allow access to the left third intercostal space. Pectoral muscles were retracted and the intercostal muscles cut caudal to the third rib. Wound hooks were placed to allow access to the heart. The pericardium was incised longitudinally and the LAD was identified. A 7.0 silk suture was placed around the LAD for permanent blocking of arterial blood flow. For the ischemia-reperfusion model, an additional piece of 2–3 mm PE 10 tubing was placed between the suture. After 60 min, the tubing was removed and the ligature cut to allow for reperfusion via the LAD. After surgery, mice were injected with 0.1 mg/kg of Buprenorphine for *Mus* and 200 mg/kg Metamizole for *Acomys*, after which the rib cage as well as skin were closed with a 5.0 silk suture. For C57BL/6 mice, the anesthetic cocktail was antagonized through an i.p. injection containing Atipamezol (2 mg/kg). For spiny mice, anesthesia was antagonized through an i.p. injection containing Atipamezol (2 mg/kg), Flumazenil (0.1 mg/kg), and Naloxone (0.6 mg/kg). The animal was disconnected from the ventilator by removing the tracheal tube and placed on a nose cone with 100% oxygen. When the animal was capable of autonomous breathing, it was moved to its cage (placed on a 40 °C heating mat) for further recovery. Subsequent pain relief during the following days was achieved through the addition of Metamizol (160 mg/kg) to the drinking water, stored in light-protected sterile bottles, for a maximum of 4 days.

### Echocardiography

Cardiac function was determined by two-dimensional transthoracic echocardiography on sedated mice (1.5–2.0% isoflurane), using a a 30 MHz transducer operated by a Vevo 2100 ultrasound scanner (FUJIFILM VisualSonics Inc.). Based on M-mode tracings, morphometric parameters, e.g. LVID;d, LVID;s, LVFW;s, LVFW;d, IVS;s and IVS;d, were measured according to the American Society of Echocardiography leading edge rule^40^. These parameters were averaged based on five measurements. FS (%) and EF (%) were calculated from the measured parameters as: ((LVID;d − LVID;s)/LVID;d) × 100%; and ((LVID;d3 − LVID;s3)/LVID;d3) × 100%, respectively, using Vevo LAB image analysis software.

### Electrocardiography (ECG)

Electrocardiographs (ECG) were obtained during echocardiography measurements by the Vevo 2100 Imaging System. For the analysis of QRS amplitude and duration, a custom script was written using .NET Core 3.1 (C# 8.0). QRS-complexes were automatically detected using peak detection software. R peak detection was based on a previously published algorithm to ensure accurate detection in severely affected infarcted hearts characterized by abnormal ECG signatures^41^. Amplitudes were determined relative to the electromagnetic neutral point. Peak durations were determined by the difference in time between the start of the peak and the end of the peak. Datasets were manually verified for correct ECG measurement and accurate peak annotation.

### Tissue collection

Mice were euthanized through 5% isoflurane followed by cervical dislocation, and the chest was opened to expose the heart. After cutting the right atrium, the heart was immediately perfused slowly by injecting 5–10 mL cold perfusion buffer (130 mM NaCl, 5 mM KCl, 0.5 mM NaH_2_PO_4_, 10 mM HEPES, 10 mM glucose, 20 mM 2,3-butanedione monoxime (Sigma, #31550), 0.1 mM Blebbistatin (Sigma, # B0560), 10 mM taurine, and 5 mM EDTA, adjusted to pH 7.8) into the left ventricle. After perfusion, the heart was removed and washed in cold perfusion buffer before further processed.

### Evans blue intravascular injection

Evans blue dye (Sigma Aldrich, #E2129) was dissolved in physiological salt solution (30 mg/mL). Animals were weighed and sedated using isoflurane (5.0% induction/2.0% maintenance). The abdomen was shaved and sterilized, and subsequently incised to expose the abdominal cavity. The inferior vena cava was located and 12 µL/kg body weight of Evans blue solution was slowly injected. After 15 min of circulation, animals were sacrificed through cervical dislocation. Immediately thereafter, the right atrium was cut, and the heart was briefly flushed by injecting 1 mL cold perfusion buffer into the left ventricle. Hearts were then snap-frozen in optimal cutting temperature compound (OCT) (Leica, #14020108926) using dry ice and stored at −20 °C. Transversal cross-sections were cut using a Cryostar NX70 cryostat (Thermo Scientific) until the desired location was reached, and an image was shot using a Nikon DSLR camera. Four equally spaced-out transversal stacks, from point of ligation down to the tip of the apex, were used for image analysis (per animal), and the mean area was used to calculate ischemic area (relative to total area). Areas were manually traced based on color using Fiji (ImageJ 1.53c, USA).

### Imaging

#### 2D imaging of tissue sections

Upon organ excision, organs were fixed overnight at 4 °C in 2% formaldehyde. The next day, fixed tissues were washed three times in PBS, embedded and frozen in OCT using dry ice and stored at −20 °C. Transversal cross-sections of 8 µm were cut using a Cryostar NX70 cryostat (Thermo Scientific). For the staining protocol, sections were permeabilized in 0.1% Triton-X-100 in PBS for approximately 30 min at RT, and then washed with PBS. Sections were then blocked for non-specific binding with 1% BSA and 10% donkey serum (Abcam, #ab7475) in PBS for 60 min at RT, and then incubated with primary antibody in 1% BSA and 5% donkey serum in PBS, O/N at 4 °C (see Table 1). The next day, following washing, sections were incubated in PBS with fluorescent secondary antibody, for 120 min at RT in the dark. Finally, sections were washed and incubated with Hoechst 33342 nucleic acid stain (Invitrogen, #H1399) or fluorescent dye (see Table 1) for 15 min, washed in PBS, mounted with ProLong™ Gold Antifade (Thermo Scientific, #P36934), and stored at RT in the dark until solidified. Samples were then imaged immediately or stored until further analysis at 4 °C in the dark. Samples were imaged using a Leica Thunder Imager.

**Table 1:** Antibody and fluorescent dye list.

	Company	Catalog number	Working dilution	Antigen retrieval
Primary antibodies				
α-SMA-647	Novus Biologicals	NBP2-34522AF647	1:100	No
Phospho-Histone H3	Novus Biologicals	NB600-1168	1:200	Yes
α-SMA	Abcam	ab5694	1:200	No
PDGFRβ	Abcam	ab32570	1:200	Yes (but not required)
ERG	Abcam	ab92513	1:200	Yes
MLC-2v	Synaptic Systems	310 003	1:200	Yes (but not required)
DDAH2	Elabscience	E-AB-10938	1:200	No
Secondary antibodies				
Goat anti-Rabbit IgG (H + L)−488	Invitrogen	A-11034	1:500	NA
Donkey anti-Rabbit IgG (H + L)−568	Invitrogen	A-10042	1:500	NA
Goat anti-Rabbit IgG (H + L)−647	Invitrogen	A-21245	1:500	NA
Goat anti-Rat IgG (H + L)−594	Invitrogen	A-11007	1:500	NA
Goat anti-Rat IgG (H + L)−647	Invitrogen	A-21247	1:500	NA
Fluorescent dyes				
Isolectin GS-IB4-568	Invitrogen	I21412	1:200	NA
Wheat Germ Agglutinin-488	Invitrogen	W11261	1:200	NA
Hoechst 33342	Invitrogen	H3570	1:2000	NA
Phalloidin-TRITC	Sigma Aldrich	P1951	1:500	NA
Dextran-FITC 150 kDa	Sigma Aldrich	46946	10 mg/mL	NA

#### 3D imaging of whole-mount tissue samples

Upon organ excision, scar area or corresponding healthy myocardium was cut out using a 4 mm biopsy punch. Tissues were then fixed overnight at 4 °C in 2% formaldehyde. The next day, fixed tissues were washed three times in PBS, and stored at RT in PBS containing 0.2% gelatin (Sigma Aldrich, #G1393), 0.5% Triton-X-100 (Sigma Aldrich, #X100) and 0.01% Thimerosal (Sigma Aldrich, #T8784) (PBS-GT) for at least 24 h. Samples stored in PBS-GT were incubated with pre-conjugated primary antibody in PBS-GT while shaking, for 36 h at RT (see Table 1). Excessive antibody was removed by thorough washing in PBS-GT for 24 h and refreshing the solution every 3 h during the day. Samples were then cleared for 36 h at RT with Vectashield (Vector, #H-1000) after which they were imaged immediately or stored at 4 °C in the dark. Samples were imaged in 35 mm glass-bottom dishes (VWR, #75856742) using a laser scanning confocal microscope (Leica SP8) whilst submerged in Vectashield. Tissue biopsies were imaged facing the outside epicardial surface to ensure imaging scar tissue and avoid the opposing strand of tissue that could still contain residual myocardium.

### Cell death assay

Detection of apoptotic cells was performed with a TUNEL assay (Sigma Aldrich, #12156792910) according to the manufacturer’s instructions. In brief, 8-µm-thick cryo-sections were fixed in 4% PFA for 20 min at room temperature and washed with PBS for 30 min. The sections were permeabilized with 0.1% Triton-X-100 (Sigma Aldrich, #X100) and 0.1% sodium citrate in PBS for 2 min and washed with PBS thereafter. Subsequently, the sections were incubated with TUNEL reaction mixture in a humidified chamber at 37 °C for 60 min in the dark, rinsed three times with PBS, counterstained with Hoechst 33342 nucleic acid stain (Invitrogen, #H1399) for 15 min and mounted with ProLong™ Gold Antifade (Thermo Scientific, #P36934).

### Dextran-FITC whole-mount imaging

Dextran-FITC (Sigma Aldrich, #46946) was dissolved in physiological salt solution (10 mg/mL) and injected intravascularly as described above for Evans blue. Following blood removal, the heart was excised and the scar area was cut out using a circular 4 mm biopsy punch. The tissue was then fixed in 4% PFA at room temperature for 60 min in the dark. After a brief wash in PBS, samples were immediately imaged using a laser scanning confocal microscope as described above for whole-mount imaging.

#### In vivo EdU labeling

Animals were injected intraperitoneally with 100 μL solution containing 1 mg/mL EdU (Invitrogen, #A10044) dissolved in sterile physiological saline, 5, 3, and 1 day(s) before the point of sacrifice. For the 4-day time point the 5-day injection point was skipped. Following organ excision and fixation overnight in 2% formaldehyde, EdU was visualized using the Click-iT™ EdU Alexa Fluor™ 647 imaging kit (Invitrogen, #C10340), according to the manufacturer’s instructions. The Click-iT® reaction cocktail was incubated with the samples for 2 h at RT. Tissues were then further processed according to the imaging protocol (see ‘2D imaging of murine tissue sections*’*).

#### Masson’s Trichrome histological staining

To visualize deposited extracellular matrix, Masson’s trichrome staining was performed (Sigma Aldrich, #HT15). In brief, samples were incubated for 15 min in Bouin’s solution (Sigma Aldrich, #HT10132) at 56 °C (water bath), and then washed under running tap water for 1 min. Samples were then immersed in Weigert’s iron hematoxylin solution (Sigma Aldrich, #HT1079) for 5–10 min (depending on freshness), and again washed under running tap water for 2 min. Samples were incubated with Briebrich Scarlet-Acid Fuchsin solution for 5 min, rinsed in dH_2_O, and incubated with Phosphotungstic/Phosphomolybdic acid solution for 5 min. Finally, samples were immersed in Aniline Blue solution for 10 min, washed in 1% acetic acid for 2 min, and further washed with dH_2_O, and then dehydrated through an ethanol gradient. Samples were then dipped 15 times and cleared in Roti®-Histol (Carl Roth, #6640) and mounted with Roti®-Histokitt (Carl Roth, #6638).

### Sirius red staining and polarized light microscopy

A picrosirius red solution was made by combining 0.5 g Direct Red 80 (Sigma Aldrich, #365548) in 500 mL of a saturated aqueous solution of picric acid (1.3% in H2O, Sigma Aldrich, #P6744). Cryo-sections were first immersed in Weigert’s iron hematoxylin solution (Sigma Aldrich, #HT1079) for 5–10 min (depending on freshness), and then washed under running tap water for 2 min. Sections were then immersed in pircrosirius red solution for exactly 1 h, and then briefly washed twice in 0.5% acetic acid solution. Sections were finally dehydrated in three changes of 100% ethanol, dipped 15 times in xylene, and mounted with Roti®-Histokitt (Carl Roth, #6638). Polarized light microscopy was then applied using an Olympus SLIDEVIEW VS200 slide scanner to capture the birefringent properties of the collagen fibers. Tissue images were obtained with a ×20 objective lens.

### Labeling of the extracellular matrix on organ surfaces

Alexa Fluor 488 conjugated succinimidyl esters (Invitrogen, #A20000) were diluted in DMSO to a final concentration of 25 mg/mL and stored at −80 °C. To obtain ectopic labeling of the extracellular matrix, a working solution was prepared by mixing NHS ester with 0.1 M sodium bicarbonate buffer (pH 9.0) in a 1:1 ratio. A circular 4 mm biopsy punch was used to prepare sterile grade 1 Whatman filter papers (Sigma Aldrich) that were soaked in 2 µL ester solution. Animals were sacrificed, the heart was flushed, and the ester-soaked filter paper was placed locally on the heart surface for 2 min, after which the filter paper was removed. The labeled tissue was then punched out using the same biopsy punch. Tissues were further processed as described above.

### Scanning electron microscopy

Animals were euthanized and perfused as described above. A circular 4 mm biopsy of the scar or corresponding sham-operated myocardium was used for imaging. After collection, tissues were briefly washed in wash buffer (0.2 M Hepes buffer solution, pH 7.40, Electron Microscopy Sciences, #11494) and then immediately fixed in 3% glutaraldehyde in 0.1 M sodium cacodylate buffer, pH 7.4 (Electron Microscopy Sciences, #16538–15) for 2 h at room temperature. Samples were then washed twice in 0.1 M cacodylate buffer pH 7.4 and dehydrated stepwise in a graded ethanol series. Samples were critical point dried in 100% ethanol with a critical point drying machine (CPD300, Leica, Austria) and afterwards glued onto leit-tabs (Plano, Germany) mounted on aluminum stubs (Plano, Germany) and were sputter-coated with a 10-nm-thin gold layer (ACD600, Leica, Austria). SEM images were acquired with a JEOL JSM-IT200 SEM (JEOL, Japan) at 10 kV with an SE detector.

### Cardiomyocyte isolation and nuclei number/cell surface determination

Ventricular tissues were fixed in 4% formaldehyde for 24 h at 4 °C, followed by incubation in 50% w/v potassium hydroxide solution (Alfa Aesar, #35621) for 16 h at 4 °C under constant agitation. Zebrafish ventricles were only incubated in potassium hydroxide for 3 h at RT. Tissues were gently crushed with tweezers to release dissociated cardiomyocytes, and then pipetted up and down using a 5 mL stripette for approximately 1 min. Cells were washed in PBS, spun down at 300 × *g* for 5 min, liquid was aspirated, and cells were again washed with PBS for a total of three times. Cells were then incubated with Hoechst 33342 nucleic acid stain (Invitrogen, #H1399) in PBS for 15 min, washed one more time, and finally suspended and mounted on a glass slide with ProLong™ Gold Antifade (Thermo Scientific, #P36934). Cells were imaged using a Leica Thunder Imager and Leica Image Files (LIFs) were imported directly in Fiji (ImageJ 1.53c, USA). Nuclei numbers were counted manually, and corresponding surface area was determined by multiplying the width and length of every cell.

### Proteomic profiling

Whole heart ventricles were snap-frozen in liquid nitrogen, and then grinded using a mortar and pestle. Approximately 20 mg of tissue per sample was used for further analysis. Tissues were incubated with 100 µL RIPA lysis buffer (65 mM Tris-HCl, 150 mM NaCl, 1% Triton-X-100, 1% Sodium deoxycholate, 1 mM EDTA, pH 7.4), and a mixture of protease inhibitors using cOmplete™ ULTRA Tablets (Roche, #5892791001). Lysates were kept on ice for 15 min, vortexed, and subsequently sonicated for three cycles (30 s ON, 30 s OFF) on low settings using a Bioruptor® Plus (Diagenode, #B01020001). Samples were then centrifuged for 10 min at 10,000 × *g* and supernatant was collected. A BCA protein assay kit (Thermo Scientific, #23225) was used to determine protein content of the supernatant fractions and 300 µg of total protein was used for the following steps. Angiogenic proteins were measured using a Mouse Angiogenesis Array Kit (R&D Systems, #ARY015) according to the manufacturer’s instructions. Digital images were quantified by densitometry using LI-COR Image Studio Lite software (version 5.2.5).

### Quantitative real-time PCR

Whole heart ventricles were snap-frozen in liquid nitrogen, and then grinded using a mortar and pestle. Total RNA was isolated from heart ventricles using a NucleoSpin RNA Plus isolation kit (Macherey-Nagel, #740984) according to the manufacturer’s instructions, and eluted in nuclease-free water (Invitrogen, #AM9937). Total RNA yield was determined with a NanoDrop ND-1000 spectrophotometer and samples were normalized accordingly. cDNA was synthesized using 300 ng total RNA per sample, using the RevertAid First Strand cDNA Synthesis Kit with random hexamer primers, according to the manufacturer’s instructions (Thermo Scientific, #K1622). The cDNA was diluted 30× with nuclease-free water and used for quantitative PCR (qPCR). Each qPCR reaction was performed in a 10 µL volume, using 5 µL 2× iTaq universal SYBR® green supermix (Biorad, #1725124), 2 µL 2.5 µM target gene-specific forward and reverse oligo mixture and 3 µL cDNA. All primer sets were tested for linearity and single melting curves. A two-step qPCR program of 40 cycles was used with anneal/elongation step of 1 min at 58 °C. All reactions were performed in technical duplicates and all targets were normalized to four housekeeping genes (18S ribosomal RNA, Ribosomal Protein L13A (RPL13A), Ribosomal Protein L32 (RPL32), and Beta-2-Microglobulin (B2M) (*Acomys* only), or TATA-Box Binding Protein (Tbp) (*Mus* only). For the maturation genes, additional non-traditional reference genes were added to account for heterogeneity between neonatal and adult samples. To find appropriate reference genes, the bulk-seq data were used to screen for statistically non-significant genes between neonate and adult samples, for all species. Based on this screen and follow-up RT-qPCR tests we selected *Arl8b*, *Larp6*, and *Nrip1* as the reference genes. Analysis of RT-qPCR data was done using LinRegPCR analysis software^42,43^. Gene units are expressed as the N0, which denotes the (unitless) RNA starting concentration. The N0 per sample is calculated in the unit of the *Y*-axis of the PCR amplification plot, which are arbitrary fluorescence units. Table 2 provides a list with all used primer sequences. A spiny mouse SequenceServer^44^ was used to BLAST and obtain *Acomys* protein-coding transcript sequences used for primer design^45^.

**Table 2:** Primer sequences.

Amplicon	Species	Forward sequence (5ʹ → 3ʹ)	Reverse sequence (5ʹ → 3ʹ)
**18s**	***Mus musculus***	**CGCCGCTAGAGGTGAAATTC**	**TTGGCAAATGCTTTCGCTC**
**Rpl13a**	***Mus musculus***	**ACCGCCCTACGACAAGAAAA**	**GCTGTCACTGCCTGGTACTT**
**Rpl32**	***Mus musculus***	**GGTGAAGCCCAAGATCGTCA**	**CTGGCCCTTGAACCTTCTCC**
**Tbp**	***Mus musculus***	**CCCACCAGCAGTTCAGTAGC**	**CTCTGCTCTAACTTTAGCACCTGTT**
**Arl8b**	***Mus musculus***	**GACTGGTTCCGTTCGCTCT**	**ATTGACCGGACGCGATGA**
**Larp6**	***Mus musculus***	**CAATGAGAACCTCCCCAGCA**	**ACCGATGAGATGACGCCAAA**
**Nrip1**	***Mus musculus***	**TGAGTCGCTTGCTGAGACAG**	**CTTCGGGACCATGCAGATGT**
Nppa	*Mus musculus*	ATCTGATGGATTTCAAGAACCTGC	TCTCAGAGGTGGGTTGACCT
Myh6	*Mus musculus*	ATAGTGGAACGCAGGGATGC	TTGACTCGCCCAAACTCCTC
Myh7	*Mus musculus*	GCCCTTTGACCTCAAGAAAGATG	CACAGTCACCGTCTTGCCA
Myh10	*Mus musculus*	CACAGCCACCCCAATCAGAA	AAGGAATCTGGGAGCTTGCC
Pfkp	*Mus musculus*	TCGGAGATTTGCAGTCCAACG	ACACTCCTTTGCCCTCCTCT
Prnp	*Mus musculus*	GACGGGAGAAGATCCAGCAG	AGACCACGAGAATGCGAAGG
Sorbs2	*Mus musculus*	ACGGATGGTTTGTGGGAACT	CCTGCTGGGATGTTTCAGGT
Mafk	*Mus musculus*	CTACCTGCCCACTGTGAACC	TCATCCTGGTTGGCCATGTG
Tnni1	*Mus musculus*	AGTGCCCTTCAGGACTTGTG	GAGGACGCTTGAACTTCCCA
Tnni3	*Mus musculus*	CTATGACCTCCGTGGCAAGTT	CACCTCCCGGTTTTCCTTCTC
Myl2	*Mus musculus*	GGGACACATTTGCTGCCCTA	TCTCTTCAGGATCAGCCCCTTT
Myl7	*Mus musculus*	TGAGTGCCTTCCGCATGTTC	AAACAGTTGCTCTACCTCAGCAG
**18s**	***Acomys cahirinus***	**TGTGGTCCTTTTCCCGTTCC**	**GAGTCCCTCGCTCACATCAC**
**Rpl13a**	***Acomys cahirinus***	**AGCAAGTTCACAGATGTCCTCA**	**GCTGTCTAAGTCTCCTCTACCATAG**
**Rpl32**	***Acomys cahirinus***	**GTTTCCTCCCCAAGAACCGAA**	**TGGGATTGGTGACTCGGATG**
**B2m**	***Acomys cahirinus***	**CGTATGCCTGCAGAGTCCAA**	**GACCTCCACGAGGCTTGATT**
**Arl8b**	***Acomys cahirinus***	**CACAGGACGAAAGGAAGGCT**	**TAGCGACGGCATTTGTGAGA**
**Larp6**	***Acomys cahirinus***	**CCTCTTAAAGCCGCAAAGGAGT**	**CCAAAAGGGTATGGGGTACGG**
**Nrip1**	***Acomys cahirinus***	**CACAGCGACGGGACTAAGTT**	**GTGGGGAGTTCCACCTGATG**
Nppa	*Acomys cahirinus*	CCACTGAGGTGTGGCGAATA	CACGCCTCTACAGTTAGCGT
Myh6	*Acomys cahirinus*	GACGACTCGGAGGAGCTTTT	ACACCAGCTTTCTCCTCTGC
Myh7	*Acomys cahirinus*	GCAGGAAGCCCACTTCTCTT	TCTCGTTGAGCGGATCCTTG
Myh10	*Acomys cahirinus*	CACACCCAGTCTTCCTAGAGC	CCCCTCACAGTTTGCTCACTA
Pfkp	*Acomys cahirinus*	TCTGCTGTTCGAGTTGGCAT	CTTTCCTGGTAGGGTGCGTT
Prnp	*Acomys cahirinus*	AGCCATTCCGTCCGGTACTA	AGGGGTCCACTTTGGATTCG
Sorbs2	*Acomys cahirinus*	ACCAACAGGCAAGGCATCTT	GGCAGACAGGTTTGTCCACT
Mafk	*Acomys cahirinus*	GGTGGGAGGTCCGTTAGGTA	ACTGCCACCTCCATATCCCT
Tnni1	*Acomys cahirinus*	CTACACGCCCACCTTTTGGA	CCCTTGGGGTAGCTGCATAA
Tnni3	*Acomys cahirinus*	CCAGAAGGGGACCCAACCTA	TAGGCTCGGTAGTTGGCAGA
Myl2	*Acomys cahirinus*	CCTTGGCTCATTGGCTCTGA	CTGATATTCTTGCTGCTTTCACAGA
Myl7	*Acomys cahirinus*	AAGTTCTCTCCTGCCGAGGT	CCCCGTGGGTGATGATGTAG

Housekeeping genes in bold.

### Image analysis

#### Scar area

Masson Trichrome histological stainings were used to mark the collagenous scar in injured hearts. Images were captured using a Leica DM4000 upright brightfield microscope, and source images were subsequently exported as TIFs. White balance was automatically corrected with the eye dropper function using Adobe Photoshop CC 2018, after which they were loaded in Fiji (ImageJ 1.53c, USA). To measure scar area, images were manually color thresholded based on Hue (140−200), Saturation (10−255), and Brightness (0−255) settings. The resulting threshold was selected and area was measured. To measure total heart area, the Hue settings were expanded (60−255) until the entire tissue section was thresholded. To account for changes in scar shape along the heart wall, six equally spaced-out transversal stacks, from point of ligation down to the tip of the apex, were used for image analysis (per animal), and the mean area was used to calculate scar area (relative to total area).

#### Heart size

For the analysis of heart size, the same set of images used to calculate scar area was used, and the corresponding mean area was used to calculate heart size. In brief, source images (Leica Imaging File) were loaded into Fiji (ImageJ 1.53c, USA), and were split into the different RGB channels. Using the green channel, images were Gaussian blurred (sigma = 50), and manually thresholded (‘Triangle’ algorithm) per batch (to account for differences in staining intensity between batches). The resulting threshold was converted to a mask (‘Black Background’ checked) and the particle was analyzed (‘Exclude on edges’, and ‘Include holes’ checked). A macro was written to automate the above steps for smooth processing.

#### General proliferation

In brief, source images (Leica Imaging File) were loaded into Fiji (ImageJ 1.53c, USA), and were split into the different fluorescent channels. Background was subtracted on all channels using the rolling ball algorithm (size = 50). To determine total nuclei and EdU+ nuclei numbers, the Hoechst or EdU channel was selected and a median filter (radius = 1) was applied. The image was subsequently thresholded using the Default (for Hoechst) or Yen (for EdU) algorithm. Particles were then analyzed (‘Exclude on edges’, and ‘Include holes’ checked) to count total nuclei numbers. To determine nuclei numbers inside the scar area, the WGA channel was selected, and the same macro was applied (Yen algorithm, but with a larger filter size (median filter, radius = 20). The resulting segmentation was added as a region of interest (ROI). EdU− and EdU+ nuclei were then counted inside the ROI.

#### Capillary proliferation

To determine the capillary area, the isolectin B4 channel was selected, images were background subtracted using the rolling ball algorithm (size = 50), and then thresholded manually using the Moments algorithm to account for the higher isolectin B4 expression in non-scar regions, therefore precluding automated thresholding. Strict criteria were used to avoid false positives. The resulting segmentation was added as an ROI, and the above settings for Hoechst, EdU, and WGA were applied to count the relevant proliferating capillaries in the scar area. To threshold ERG+ and phosphorylated Histone H3 + nuclei, the Moments algorithm was used.

#### Artery numbers

Source images (Leica Imaging File) were loaded into Fiji (ImageJ 1.53c, USA), and were split into the different fluorescent channels. The α-SMA and WGA channel were selected and stacks were merged via Maximum Intensity Z-projection with a 50% transparency setting. All α-SMA+ circular- and tubular-shaped vessels were then manually counted in the WGA+ area. Additionally, the largest diameter of each vessel was measured to be able to exclude all vessels <10 µm in diameter (to prevent the inclusion of α-SMA + mesenchyme that could be mistaken for small vessels). The above settings for WGA were applied to calculate the scar area.

#### Quantification of dividing cardiomyocytes

To detect EdU+/phosphorylated Histone H3 + cardiomyocytes (CMs), the MLC-2v channel was selected, background subtraction was performed using the rolling ball algorithm (size = 50), and a median filter was applied (size = 1) followed by automated thresholding (Default algorithm). To ensure there were no non-CM nuclei inside the MLC-2v segmentation, a subsequent mask containing all capillaries (which are normally closely intermingled with CMs) was generated that could then be subtracted from the MLC-2v segment. Because the scar region was not relevant here (due to absence of CMs), the Isolectin B4 + capillary area could be automatically thresholded (rolling ball algorithm of size 5 and median filter of radius 20) using the Default algorithm. These settings were less strict as the manual approach, to ensure a faithful exclusion of proliferating capillaries from the MLC-2v segment. Settings for Hoechst and EdU/phosphorylated Histone H3 were then applied as described above. The Image calculator was finally used to determine nuclei numbers whose signal overlapped with that of EdU/phosphorylated Histone H3 and CMs but not with isolectin B4.

#### Fibroblast coverage

The same method was applied as described above. The PDGFRβ channel was used to measure fibroblast coverage. Images were background subtracted using the rolling ball algorithm (size = 50), and then thresholded using the Moments algorithm.

#### Birefringence hue analysis

Quantification of polarized light images was conducted similar to previously established protocols, based on the principle that as fiber thickness increases, hue properties change from green to yellow to orange to red^14^. In brief, source files were exported to TIFs and loaded into Fiji (ImageJ 1.53c, USA). Background color was adjusted by subtracting the mean histogram pixel value of the background area using the math–subtract function. Images were then manually color thresholded based on different Hue, Saturation, and Brightness settings, according to previous work^46^: red (H 1–13, S 10–255, B 20–255), orange (H 14–25, S 10–255, B 20–255), yellow (H 26–52, S 10–255, B 20–255), and green (H 53–110, S 10–255, B 20–255). Only signal within the scar area was quantified, which was manually traced. Total birefringence (sum of all colors) was set to 100% and the individual hues were expressed relative to one another.

#### Waviness index

NHS ester labeled tissues imaged whole mount were used to measure collagen waviness. At least six different frames across the scar surface were used for subsequent image analysis. Waviness parameters were measured using NeuronJ, an ImageJ plugin originally developed for neurite tracing and analysis. A batch script was written to convert all TIF exported images to 8-bit. Images were then loaded into Fiji (ImageJ 1.53c, USA) with the NeuronJ plugin active. At least ten fibers were traced per image frame and the total fiber distance was measured (curved length). A tangential straight line connecting the beginning and the end of each traced fiber contour was then measured (linear length). The waviness index (W) was calculated using the formula: W = Length [curved] / Length [linear].

#### Anisotropy

NHS ester labeled tissues imaged whole mount were used to measure the isotropic value of the collagen fibers. At least six different frames across the scar surface were used for subsequent image analysis. The ImageJ plugin FibrilTool^47^ was used to quantify fibrillar alignment and anisotropy. TIF exported images were loaded in Fiji (ImageJ 1.53c, USA), and at least two ROIs per frame were drawn and saved. An automated batch script was used (github.com/marionlouveaux) to process all images and quantify the corresponding anisotropy value.

#### Fractal dimension and lacunarity

NHS ester labeled tissues imaged whole mount were used to measure the isotropic value of the collagen fibers. At least six different frames across the scar surface were used for subsequent image analysis. Fractal analysis was performed using the ImageJ plugin ‘FracLac’. Prior to loading the images, TIF exported images were background subtracted using a rolling ball radius of 75 pixels. An unsharp mask with a radius of 2 pixels and mask weight of 0.60 was applied as well as a median filter with a radius of 1. Images were converted to 8-bit and then auto-thresholded using the Default white algorithm, and finally despeckled to remove residual noise. Resulting images were saved and loaded into the FracLac plugin. Fractal dimension and lacunarity values were calculated using the box counting scan (slipping and tighten grids at default sampling sizes, minimum pixel density threshold of 0.40).

### Hydroxyproline assay

Total collagen content was determined using a hydroxyproline assay (Sigma Aldrich, #MAK008-1KT) according to the manufacturer’s instructions. In brief, whole heart ventricles were snap-frozen in liquid nitrogen, and then grinded using a mortar and pestle. 100 µL 37% (w/w) hydrochloric acid was added per 10 mg of dry tissue in pressure-tight polypropylene vials with PTFE-lined caps and then hydrolyzed at 120 °C for 90 min. Samples were spun down at 13,000 × *g* for 5 min at room temperature, and the supernatant was collected, avoiding precipitates. 60 µL per sample was added per well in a 96-well flat bottom plate and then left to dehydrate for approximately 2 h at 60 °C in an incubator. 100 µL of the Chloramine T/Oxidation buffer mixture was then added to each sample and standard, and left to incubate at room temperature for 5 min. Finally, 100 µL of diluted DMAB reagent was added per sample and standard, and incubated for another 90 min at 60 °C in an incubator. Absorbance was measured at 560 nm.

### TNBSA cross-linking assay

Whole heart ventricles were snap-frozen in liquid nitrogen, and then grinded using a mortar and pestle. Sodium bicarbonate (pH 8.5) was added per 10 mg of dry tissue in pressure-tight polypropylene vials with PTFE-lined caps. A standard curve was made by preparing a serial dilution of a 5 mg/mL Glycine stock (Fisher Scientific, #BP-381) with 0.1 M sodium bicarbonate (pH 8.5). 250 µL per 10 mg dry tissue of 0.01% (w/v) TNBSA solution (Thermo Scientific, # 28997) was added to each collagen sample and standard curve, mixed with a benchtop vortex, and incubated for 2 h at 37 °C in a thermo block at 800 rpm protected from light. 250 µL per 10 mg dry tissue of 10% (w/v) SDS and 125 µL per 10 mg dry tissue of 1 M HCl was added per sample to stop the reaction, and then hydrolyzed at 120 °C for 20 min. Samples were spun down at 13,000 × *g* for 1 min at room temperature, and the supernatant was collected. 100 µL per sample was added per well in a 96-well flat bottom plate and absorbance was measured at 335 nm using a CLARIOstar Plus microplate reader.

### Bulk sequencing

#### Pre-processing

Whole heart ventricles were snap-frozen in liquid nitrogen, and then grinded using a mortar and pestle. Total RNA was isolated from approximately 20 mg of tissue using TRIzol Reagent (Invitrogen, #15596018). After RNA extraction, pellets were resuspended with barcoded primers (containing an anchored polyT, 6 bp unique barcode, 6 bp unique molecular identifier (UMI), 5ʹ Illumina adapter (used in the Illumina small RNA kit) and a T7 promoter). Barcode design was such that each pair differed by at least two nucleotides, so that a single sequencing error will not produce the wrong barcode. RNA samples were reverse transcribed to generate cDNA, pooled, and in vitro transcribed for linear amplification using the MessageAmp™ II aRNA Amplification Kit (Invitrogen, #AM1751) according to the CEL-seq protocol^48^. Illumina sequencing libraries were prepared using the TruSeq Small RNA Library Prep Kit (Illumina, #RS-200), followed by PCR amplification for 14 rounds. Afterwards, libraries were sequenced paired-end at 26 bp read length for read 1 and 62 bp for read 2, using Illumina NextSeq500. Read 1 contained barcode information, whereas read 2 was used for alignment. The obtained reads were aligned to the mouse transcriptome (GRCm39) using Burrows−Wheeler Aligner’s Smith−Waterman alignment^49^.

#### Differential expression analysis

After obtaining the raw read count expression matrix, samples were normalized and further processed in R using the DESeq2 Bioconductor package (version 3.12)^50^. Values were transformed using regularized-logarithm transformation (Rld), and log2-transformed fold changes were shrunk using the apeglm method. An additive model was employed to compare the effects of age, while controlling for species. After differential expression analyses between relevant samples, the DESeq2 results table was exported for analysis using secondary platforms.

#### Gene set enrichment analysis

Differential analysis data from the DESeq2 pipeline were used for enrichment analysis, using the Bioconductor R package clusterProfiler^51^. Gene symbols were mapped to EntrezIDs using the org.Mm.eg.db R package^52^, and sorted in decreasing order based on the log(2) fold-change. Genes were considered significantly differentially expressed if they had FDR-adjusted values of *P* < 0.05 and a log(2) fold chance threshold of 2.0 unless otherwise stated. The Reactome mouse database was used as a source of pathway data, using the Bioconductor ReactomePA R package^53^. The *compareCluster* function was used to calculate and compare gene clusters between samples (up- or downregulated enrichment terms).

#### Pairwise correlation matrix

Using R, we performed PCA on variable genes (variance > 0.5) expressed (>1 FPKM) in more than two samples. We extracted the genes correlating and anti-correlating with PC1–5, using an absolute PC loading threshold >0.2 with a maximum of 50 genes per principal component to avoid individual principal components swamping the analysis, resulting in 300 genes. To construct the network, we computed a pairwise correlation matrix for all samples, using genes discovered in the PCA analysis. We then generated a weighted adjacency network graph using the graph.adjacency() command in igraph and visualized samples as vertices connected to other samples via edges if the Pearson pairwise correlation between two cells was higher than 0.6. The Fruchterman–Reingold layout was used to plot the network graph.

#### Cell cycle prediction

Cell cycle prediction was applied to classify samples into G1, G2/M, or S phase^23^ based on expression patterns of pre-trained classifiers. Results were generated as part of the single-cell R-analysis tools (SCRAT) pipeline^54^.

### Statistical analyses

Box plots represent the median, interquartile range (IQR), minimum (25th percentile – 1.5 × IQR), maximum (75th percentile – 1.5 × IQR) and presence of outliers marked by thick asterisks. All bar graphs represent the mean ± standard error of the mean (SEM). The majority of plots were graphed in R using the ggplot2 package (version 3.3.3), whereas all remaining plots were graphed using GraphPad Prism version 7 (GraphPad Software, Inc.).

Shapiro−Wilk and D’Agostino & Pearson test (*p* > 0.05) were used to test whether samples were normally distributed (approximately), using the R package rstatix (version 0.7.0) and accompanying ggpubr package (0.4.0) for visual inspection using QQ plots. All statistical tests were carried out using the same rstatix R package. Two group comparisons were made using an unpaired Student’s *t* test for normally distributed data. The majority of analyses were performed using a two-way mixed ANOVA in order to compare the means of groups cross-classified by two independent categorical variables, including species (between-subjects variable) and time after injury (within-subjects variable). Post-hoc testing was carried out using Bonferroni. A value of *p* < 0.05 was considered statistically significant, where * is *p* < 0.05, ** is *p* < 0.01, and *** is *p* < 0.001.

### Reporting summary

Further information on research design is available in the Nature Research Reporting Summary linked to this article.

## Supplementary information

Supplementary information

Reporting Summary

## Data Availability

The sequencing datasets generated in this study are available under BioProject accession number PRJNA692361.

## Code availability

Available upon request.

## References

[1] Bui AL, Horwich TB, Fonarow GC (2011). Epidemiology and risk profile of heart failure. Nat. Rev. Cardiol..

[2] Riehle C, Bauersachs J (2019). Small animal models of heart failure. Cardiovasc. Res..

[3] Kikuchi K (2010). Primary contribution to zebrafish heart regeneration by gata4(+) cardiomyocytes. Nature.

[4] Jopling C (2010). Zebrafish heart regeneration occurs by cardiomyocyte dedifferentiation and proliferation. Nature.

[5] Porrello ER (2011). Transient regenerative potential of the neonatal mouse heart. Science.

[6] Seifert AW (2012). Skin shedding and tissue regeneration in African spiny mice (Acomys). Nature.

[7] Gawriluk TR (2016). Comparative analysis of ear-hole closure identifies epimorphic regeneration as a discrete trait in mammals. Nat. Commun..

[8] Gaire J (2021). Spiny mouse (Acomys): an emerging research organism for regenerative medicine with applications beyond the skin. Npj Regen. Med..

[9] Peng, H. et al. Adult spiny mice (Acomys) exhibit endogenous cardiac recovery in response to myocardial infarction. *Npj Regen. Med.* Preprint at *bioRxiv*10.1101/2020.09.29.317388 (2021). 10.1038/s41536-021-00186-4 PMC8599698 34789749

[10] Thygesen K (2007). Universal definition of myocardial infarction. Circulation.

[11] Emde B, Heinen A, Gödecke A, Bottermann K (2014). Wheat germ agglutinin staining as a suitable method for detection and quantification of fibrosis in cardiac tissue after myocardial infarction. Eur. J. Histochem..

[12] Cohn JN, Ferrari R, Sharpe N (2000). Cardiac remodeling—concepts and clinical implications: a consensus paper from an international forum on cardiac remodeling. J. Am. Coll. Cardiol..

[13] Vigil-Garcia, M. et al. Gene expression profiling of hypertrophic cardiomyocytes identifies new players in pathological remodeling. *Cardiovasc. Res.*10.1093/cvr/cvaa233 (2020). 10.1093/cvr/cvaa233 PMC8152696 32717063

[14] Junqueira LC, Montes GS, Sanchez EM (1982). The influence of tissue section thickness on the study of collagen by the Picrosirius-polarization method. Histochemistry.

[15] Norton GR (1997). Myocardial stiffness is attributed to alterations in cross-linked collagen rather than total collagen or phenotypes in spontaneously hypertensive rats. Circulation.

[16] Badenhorst D (2003). Cross-linking influences the impact of quantitative changes in myocardial collagen on cardiac stiffness and remodelling in hypertension in rats. Cardiovasc. Res..

[17] Berthod F (2001). Collagen fibril network and elastic system remodeling in a reconstructed skin transplanted on nude mice. Matrix Biol. J. Int. Soc. Matrix Biol..

[18] Hirose K (2019). Evidence for hormonal control of heart regenerative capacity during endothermy acquisition. Science.

[19] Patterson M (2017). Frequency of mononuclear diploid cardiomyocytes underlies natural variation in heart regeneration. Nat. Genet..

[20] Camp JG (2015). Human cerebral organoids recapitulate gene expression programs of fetal neocortex development. Proc. Natl Acad. Sci. USA.

[21] Yuxuan G, Pu William T (2020). Cardiomyocyte maturation. Circ. Res..

[22] Bedada FB (2014). Acquisition of a quantitative, stoichiometrically conserved ratiometric marker of maturation status in stem cell-derived cardiac myocytes. Stem Cell Rep..

[23] Scialdone A (2015). Computational assignment of cell-cycle stage from single-cell transcriptome data. Methods.

[24] Birdsey GM (2008). Transcription factor Erg regulates angiogenesis and endothelial apoptosis through VE-cadherin. Blood.

[25] Cao J, Poss KD (2018). The epicardium as a hub for heart regeneration. Nat. Rev. Cardiol..

[26] Baradi AF, Rao SN (1976). A scanning electron microscope study of mouse peritoneal mesothelium. Tissue Cell.

[27] Goldman JA, Poss KD (2020). Gene regulatory programmes of tissue regeneration. Nat. Rev. Genet..

[28] Wei K (2015). Epicardial FSTL1 reconstitution regenerates the adult mammalian heart. Nature.

[29] Nakada Y (2017). Hypoxia induces heart regeneration in adult mice. Nature.

[30] Bassat E (2017). The extracellular matrix protein agrin promotes heart regeneration in mice. Nature.

[31] Aharonov A (2020). ERBB2 drives YAP activation and EMT-like processes during cardiac regeneration. Nat. Cell Biol..

[32] Masci PG (2011). Relationship between location and size of myocardial infarction and their reciprocal influences on post-infarction left ventricular remodelling. Eur. Heart J..

[33] Cuculich PS (2011). The electrophysiological cardiac ventricular substrate in patients after myocardial infarction: noninvasive characterization with electrocardiographic imaging. J. Am. Coll. Cardiol..

[34] Stewart DC (2018). Unique behavior of dermal cells from regenerative mammal, the African Spiny Mouse, in response to substrate stiffness. J. Biomech..

[35] Kramer DG (2010). Quantitative evaluation of drug or device effects on ventricular remodeling as predictors of therapeutic effects on mortality in patients with heart failure and reduced ejection fraction: a meta-analytic approach. J. Am. Coll. Cardiol..

[36] González-Rosa JM (2018). Myocardial polyploidization creates a barrier to heart regeneration in zebrafish. Dev. Cell.

[37] Salimova E (2019). Variable outcomes of human heart attack recapitulated in genetically diverse mice. Npj Regen. Med..

[38] Simkin, J., Gawriluk, T. R., Gensel, J. C. & Seifert, A. W. Macrophages are necessary for epimorphic regeneration in African spiny mice. *eLife* **6** (2017). 10.7554/eLife.24623 PMC5433844 28508748

[39] Qi Y (2021). Functional heart recovery in an adult mammal, the spiny mouse. Int. J. Cardiol..

[40] Sahn DJ, DeMaria A, Kisslo J, Weyman A (1978). Recommendations regarding quantitation in M-mode echocardiography: results of a survey of echocardiographic measurements. Circulation.

[41] Sadhukhan D, Mitra M (2012). R-peak detection algorithm for Ecg using double difference and RR interval processing. Procedia Technol..

[42] Ruijter JM (2009). Amplification efficiency: linking baseline and bias in the analysis of quantitative PCR data. Nucleic Acids Res..

[43] Ruijter JM (2013). Evaluation of qPCR curve analysis methods for reliable biomarker discovery: bias, resolution, precision, and implications. Methods.

[44] Priyam A (2019). Sequenceserver: a modern graphical user interface for custom BLAST databases. Mol. Biol. Evol..

[45] Mamrot J (2017). De novo transcriptome assembly for the spiny mouse (Acomys cahirinus). Sci. Rep..

[46] Bauman TM (2014). Characterization of fibrillar collagens and extracellular matrix of glandular benign prostatic hyperplasia nodules. PLoS ONE.

[47] Boudaoud A (2014). FibrilTool, an ImageJ plug-in to quantify fibrillar structures in raw microscopy images. Nat. Protoc..

[48] Hashimshony T, Wagner F, Sher N, Yanai I (2012). CEL-Seq: single-cell RNA-seq by multiplexed linear amplification. Cell Rep..

[49] Li H, Durbin R (2010). Fast and accurate long-read alignment with Burrows–Wheeler transform. Bioinformatics.

[50] Love MI, Huber W, Anders S (2014). Moderated estimation of fold change and dispersion for RNA-seq data with DESeq2. Genome Biol..

[51] Yu G, Wang L-G, Han Y, He Q-Y (2012). clusterProfiler: an R package for comparing biological themes among gene clusters. OMICS. J. Integr. Biol..

[52] Carlson, M. *org.Mm.eg.db: Genome Wide Annotation for Mouse* (2019). R Bioconductor package.

[53] Yu G, He Q-Y (2016). ReactomePA: an R/Bioconductor package for reactome pathway analysis and visualization. Mol. Biosyst..

[54] Camp JG (2017). Multilineage communication regulates human liver bud development from pluripotency. Nature.

