# Supplementary material for: Ischemic tolerance and cardiac repair in the spiny mouse (Acomys)
Source: NPJ Regen Med. 2021 Nov 17;6:78. doi: 10.1038/s41536-021-00188-2 (PMC8599451; doi:10.1038/s41536-021-00188-2)
Supplement: Supplementary file 1 — Supplementary information [file 41536_2021_188_MOESM1_ESM.pdf]

Supplementary figure 1

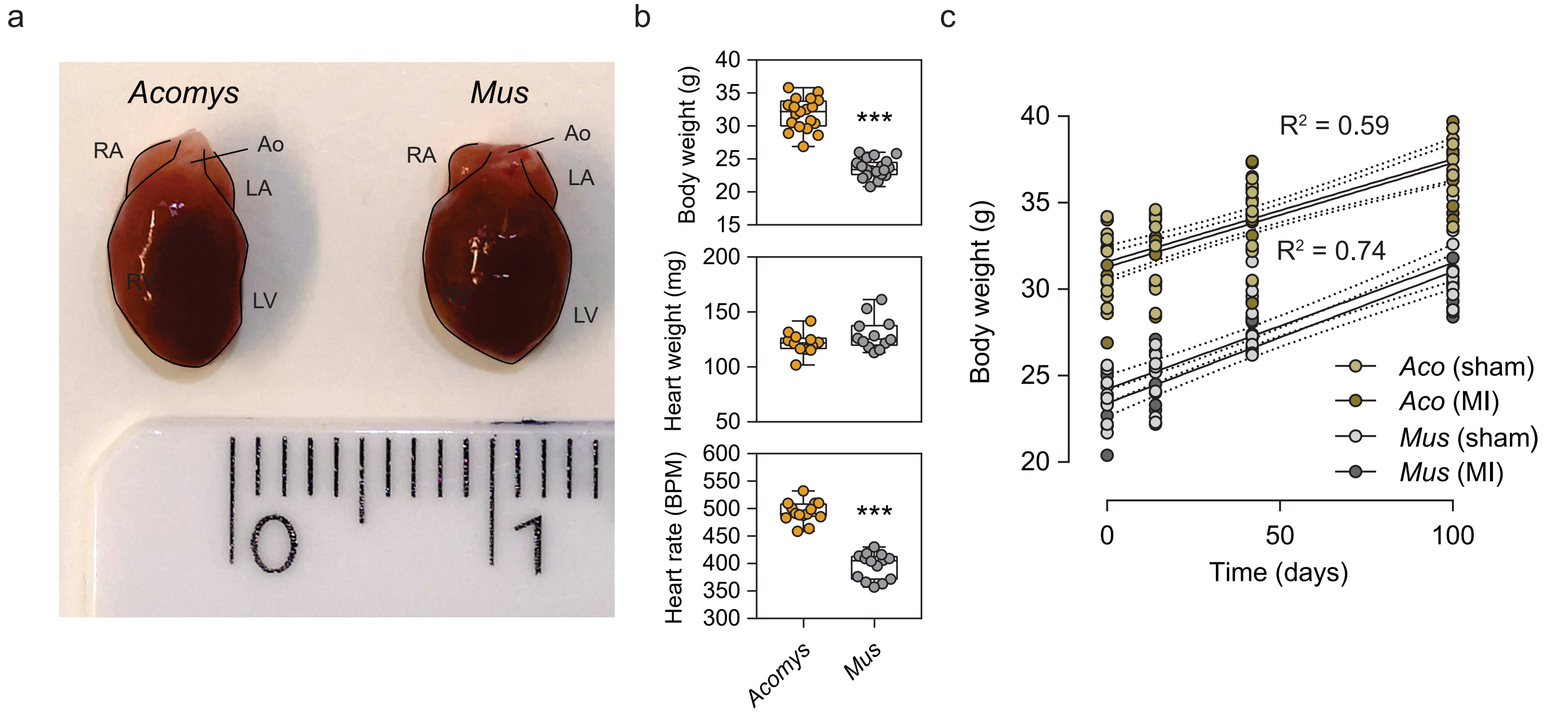

Supplementary figure 1. Basic metrics underscore anatomical similarities of the heart. (a) Photograph of a healthy adult *Acomys* and *Mus* heart. RA (right atrium), LA (left atrium), RV (right ventricle), LV (left ventricle), Ao (aorta). Attached ruler displays metric centimeters. **(b)** Basic metrics comparison between *Acomys* and *Mus*. Unpaired t-test. **(c)** Body weight progression after sham- or MI-surgery. Solid line represents a fitted linear regression. Dashed lines mark the 95% confidence interval boundaries of the best-fit line. All graphs represent the mean ± standard error of the mean (SEM).

Supplementary figure 2

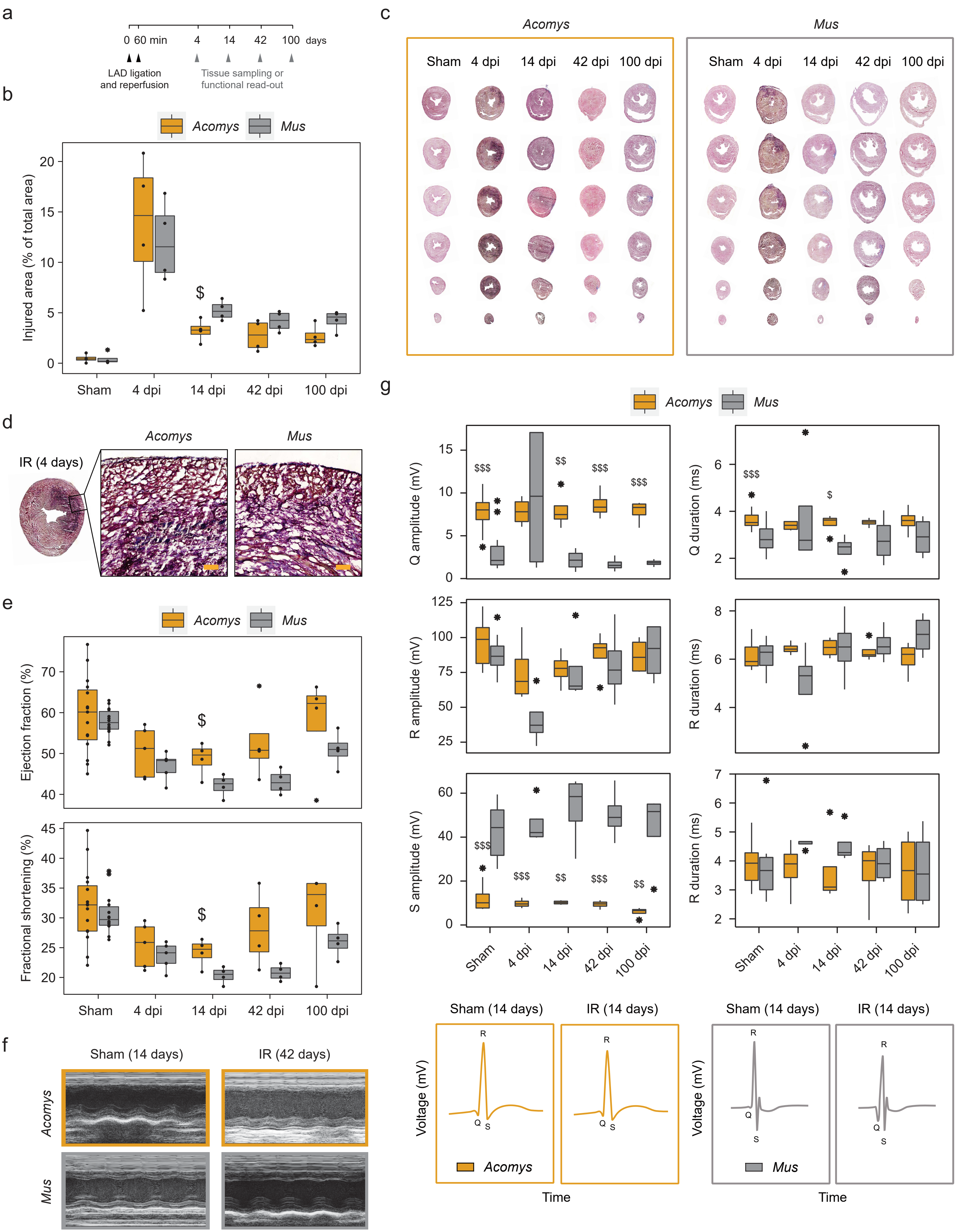

Supplementary figure 2. Ischemic reperfusion in *Acomys* and *Mus*. (a) Overview showing the different time points after reperfusion surgery, used for functional measurements or tissue sampling. **(b)** Injured area (% of total area) development over time, based on transversal stacks stained with Masson's Trichrome (see 'Methods'). **(c)** Representative images from (b), showing transversal progression from point of ligation down to the apex. **(d)** Representative close-up images of a Masson's Trichrome stain showing the injured area at 4 days post-IR. Scale bar = 100 µm. **(e)** Echocardiographic quantification of cardiac output after temporary ligation of the LAD for 60 min (reperfusion injury) or sham-control, including ejection fraction (volumetric percentage of fluid ejected from the chamber with each contraction), and fractional shortening (percentage of diastolic dimension that is lost in systole). Graph contains animals that were only measured at one time point (unpaired samples), and some that were measured over different time points (paired samples). **(f)** Corresponding representative images of the M-mode recording shown in (e). **(g)** Electrocardiogram quantification (see 'Methods') showing QRS wave parameters (amplitude and duration) after IR or sham-control, with corresponding representative illustration of the QRS wave. Graph contains animals that were measured over different time points (paired samples), as well as animals that were only measured at one time point (unpaired samples). n = (from left to right) 15, 5, 4, 4, 4, 14, 5, 4, 4, 4. b, e, g: Two-way mixed ANOVA followed by Bonferroni post hoc test. For all comparisons: * is significant compared to *Acomys*-sham, # compared to *Mus*-sham, $ is significant in an inter-species comparison of the same time point (e.g. 100 days to 100 days). * is $p < 0.05$, ** is $p < 0.01$, and *** is $p < 0.001$. Box plots represent the median, interquartile range (IQR), minimum (25th percentile – 1.5 * IQR), and maximum (75th percentile – 1.5 * IQR).

Supplementary figure 3

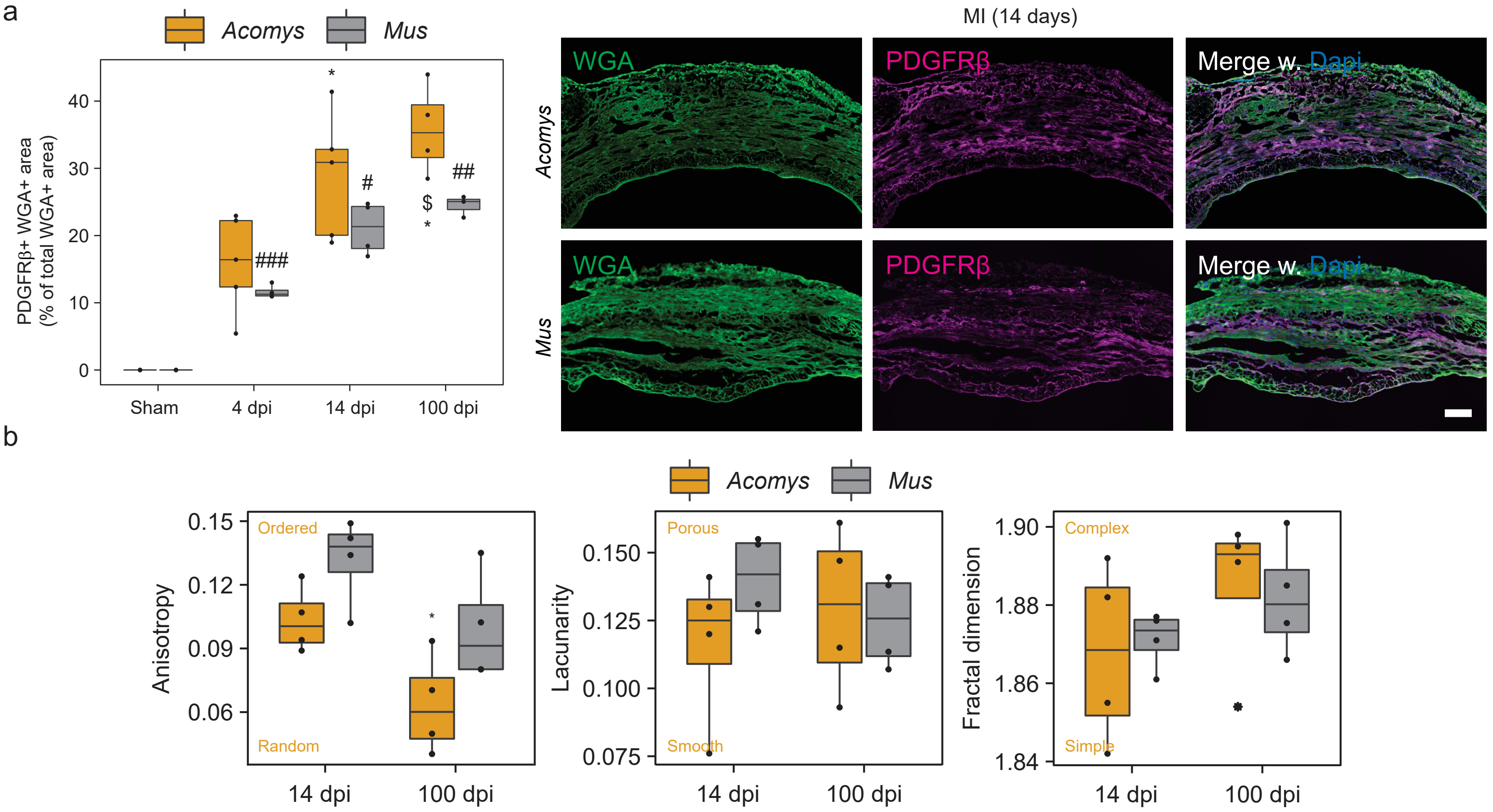

Supplementary figure 3. Scar organization in *Acomys* and *Mus* after cardiac injury. (a) Relative fibroblast coverage in the scar of infarcted hearts, marked by the pan-fibroblast marker PDGFRβ. Scale bar = 200 μm. Two-way mixed ANOVA followed by Bonferroni post hoc test. * is significant compared to *Acomys*-sham, # compared to *Mus*-sham, $ is significant in an inter-species comparison of the same time point (e.g. 100 days to 100 days). * is $p < 0.05$, ** is $p < 0.01$, and *** is $p < 0.001$. **(b)** Extracellular matrix parameters (anisotropy, lacunarity, fractal dimension), quantified from Figure 4d. Box plots represent the median, interquartile range (IQR), minimum (25th percentile – 1.5 * IQR), and maximum (75th percentile – 1.5 * IQR).

Supplementary figure 4

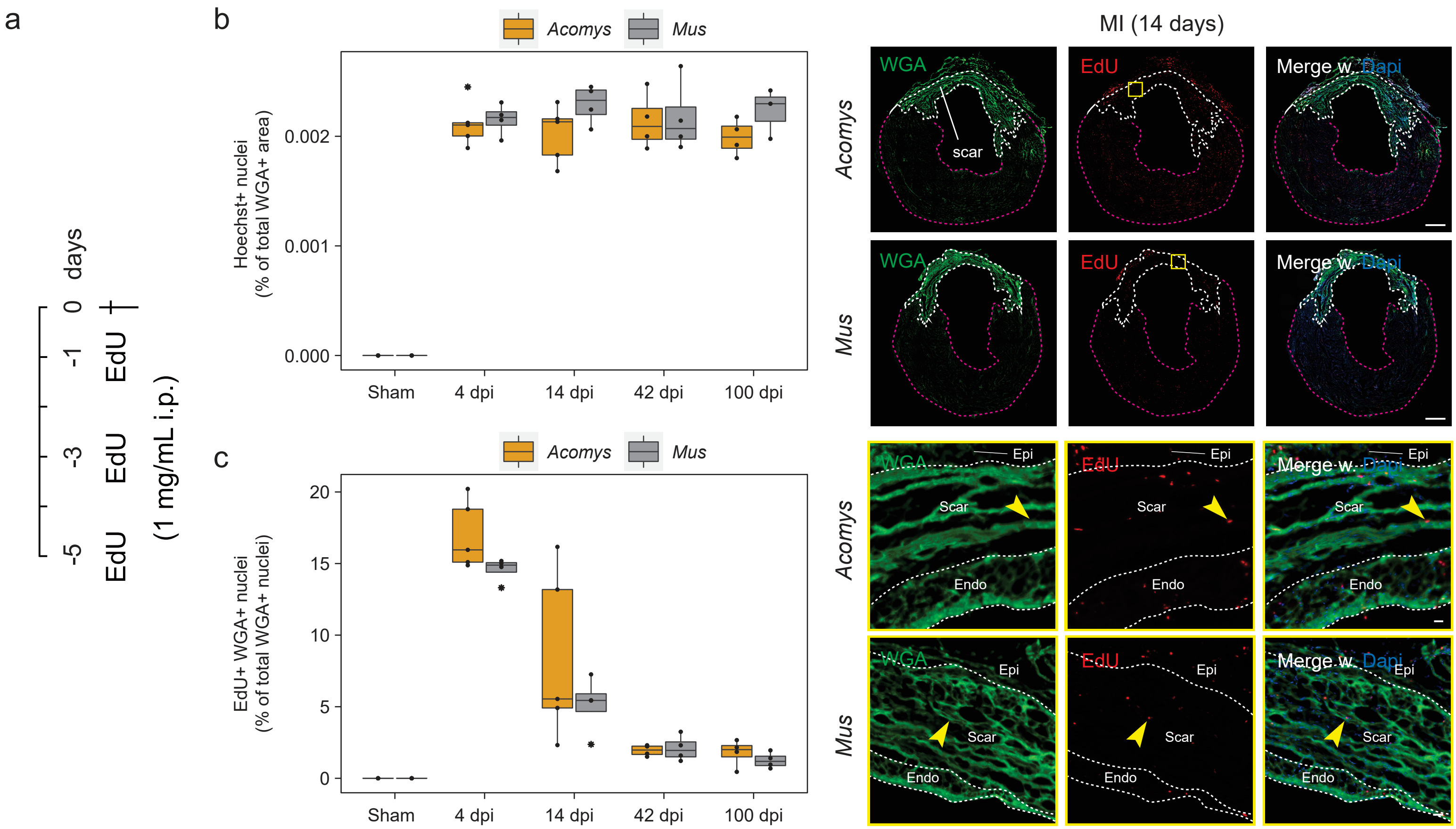

Supplementary figure 4. Proliferation dynamics in the heart. (a) Overview of the EdU injection strategy. **(b)** Cellularity of the scar, quantified using Hoechst. **(c)** Global proliferation dynamics in the scar, marked by EdU+ cells (yellow arrows). Scale bar = 700 µm (overview) or 30 µm (inlet). Epi = epicardium, Endo = endocardium. b, c: Two-way mixed ANOVA followed by Bonferroni post hoc test. For all comparisons: * is significant compared to *Acomys*-sham, # compared to *Mus*-sham, $ is significant in an inter-species comparison of the same time point (e.g. 100 days to 100 days). * is $p < 0.05$, ** is $p < 0.01$, and *** is $p < 0.001$. Box plots represent the median, interquartile range (IQR), minimum (25th percentile – 1.5 * IQR), and maximum (75th percentile – 1.5 * IQR).

Supplementary figure 5

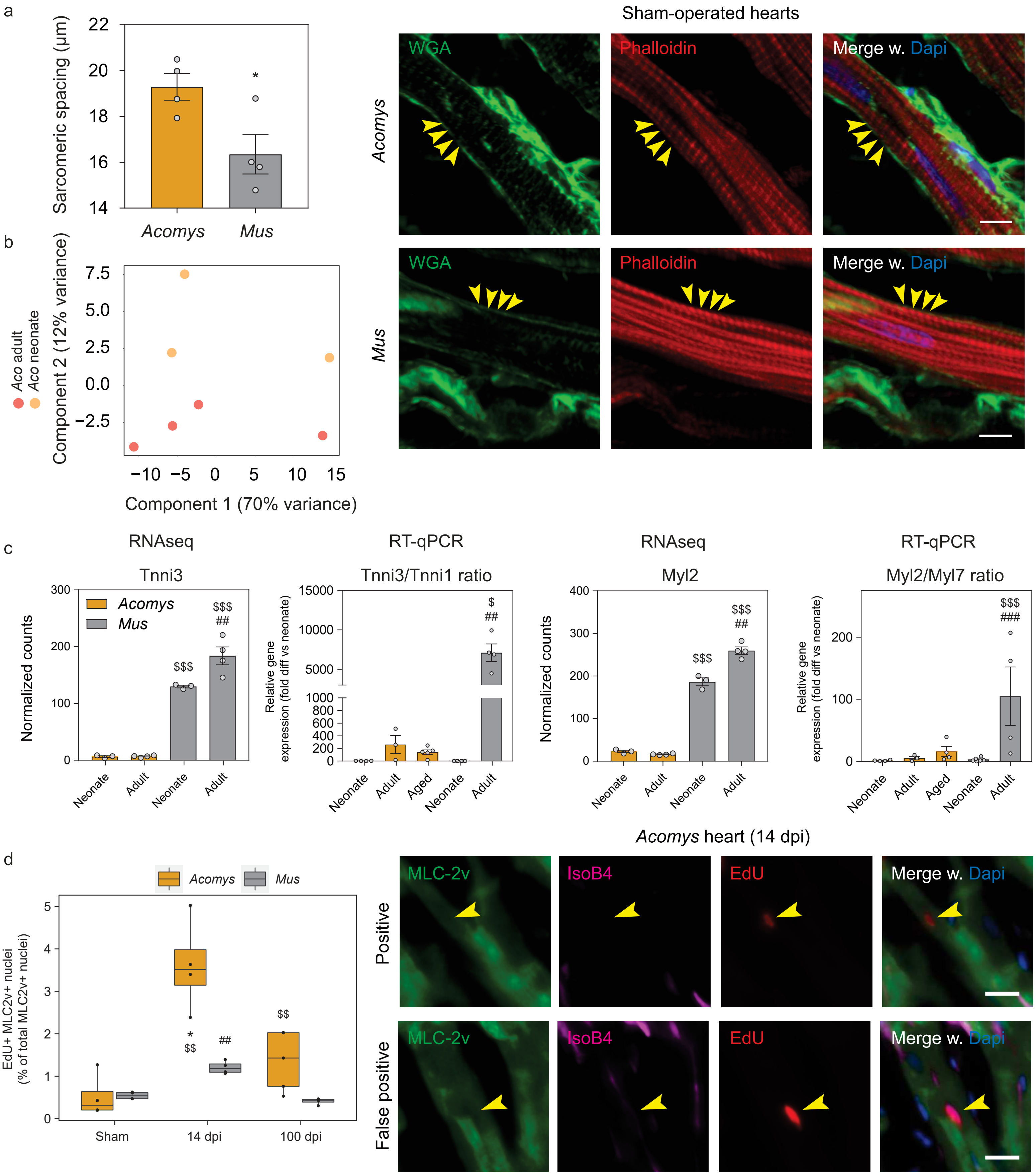

Supplementary figure 5. Cardiomyocyte properties in Acomys and Mus hearts. (a) Distance between sarcomeres (yellow arrows) of healthy adult hearts, measured using a Phalloidin dye stain. Scale bar = 5 μm. **(b)** Sample-to-sample distance visualization through principal component analysis (PCA). **(c)** Normalized counts from RNAseq data and accompanying relative gene expression from whole heart ventricle homogenates subjected to RT-qPCR. Samples have been normalized to the sham-control (*Acomys* or *Mus*). **(d)** CM (marked by MLC-2v) proliferation that are isolectin B4 negative (to exclude capillaries) in heart ventricles, indicated by EdU. Panels at the right side indicate representative fluorescent antibody or dye images showing proliferating (positive and false positive) CMs (yellow arrows). Scale bar = 15 μm. c, d: Two-way mixed ANOVA followed by Bonferroni post hoc test. For all comparisons: * is significant compared to *Acomys*-sham, # compared to *Mus*-sham, $ is significant in an inter-species comparison of the same time point (e.g. 100 days to 100 days). * is $p < 0.05$, ** is $p < 0.01$, and *** is $p < 0.001$. Box plots represent the median, interquartile range (IQR), minimum (25th percentile – 1.5 * IQR), and maximum (75th percentile – 1.5 * IQR). All bar graphs represent the mean ± standard error of the mean (SEM).

Supplementary figure 6

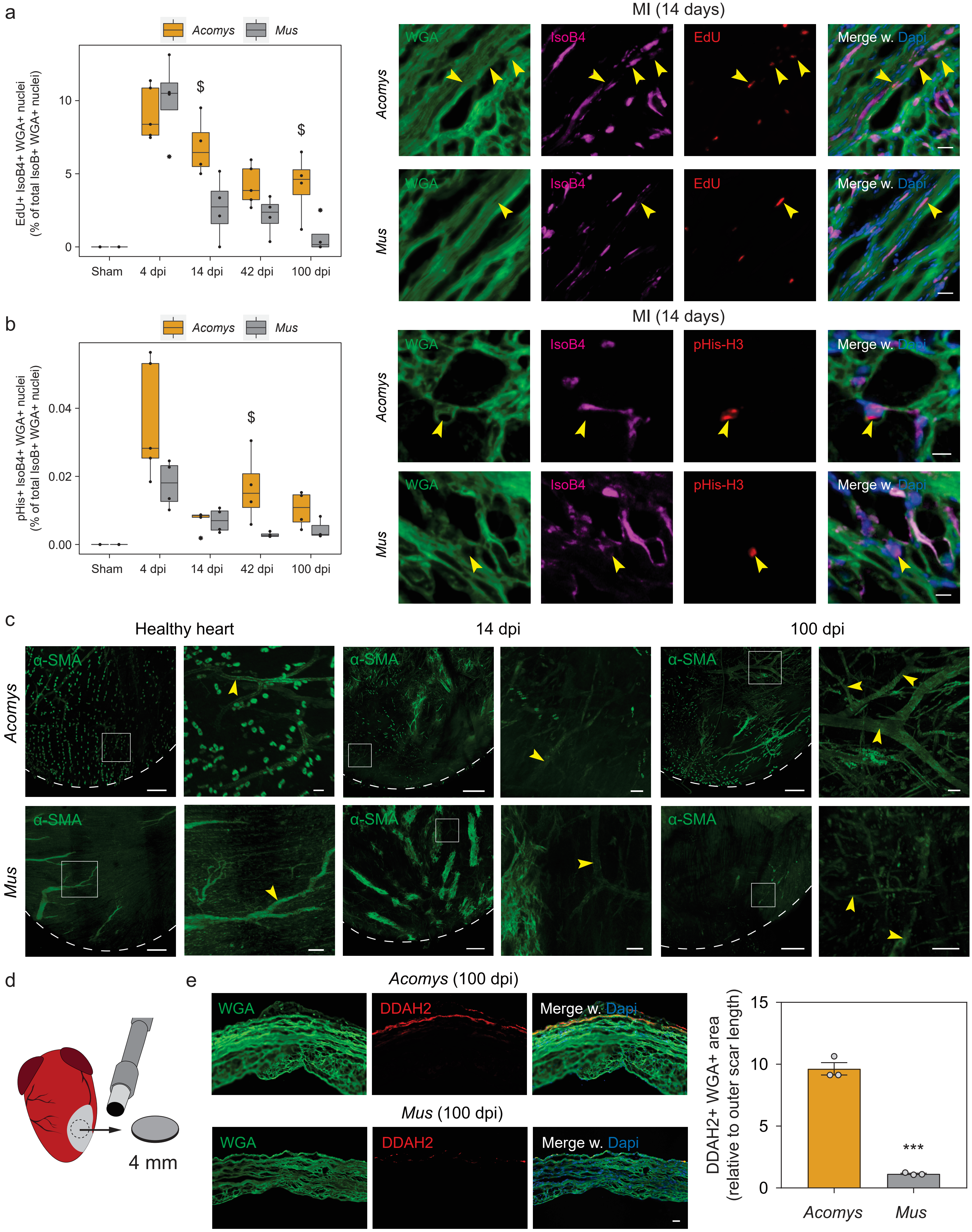

Supplementary figure 6. Angiogenesis in infarcted Acomys hearts. (a) Capillary (marked by isolectin B4, yellow arrows) proliferation in the scar area of infarcted hearts, indicated by EdU labeling. Scale bar = 20 µm. **(b)** Capillary (marked by isolectin B4, yellow arrows) proliferation in the scar area of infarcted hearts, indicated by phosphorylated Histone H3 (ser 28) labeling. Scale bar = 10 µm. **(c)** Whole mount confocal images showing α-SMA+ vessels inside the healthy heart or scar area of 14/100 day old infarcted hearts. Yellow arrows point towards vessels. Scale bar = 300 µm (overview) or 50 µm (inlet). Experiment was repeated two times per condition. **(d)** Schematic of how imaged tissues in (c) were obtained. **(e)** Epicardial immunofluorescence staining of the scar area of infarcted hearts, marked by DDAH2 labeling. Unpaired t-test. Scale bar = 40 µm. a, b: Two-way mixed ANOVA followed by Bonferroni post hoc test. For all comparisons: * is significant compared to *Acomys*-sham, # compared to *Mus*-sham, $ is significant in an inter-species comparison of the same time point (e.g. 100 days to 100 days). * is $p < 0.05$, ** is $p < 0.01$, and *** is $p < 0.001$. Box plots represent the median, interquartile range (IQR), minimum (25th percentile – 1.5 * IQR), and maximum (75th percentile – 1.5 * IQR). All bar graphs represent the mean ± standard error of the mean (SEM).

Supplementary figure 7

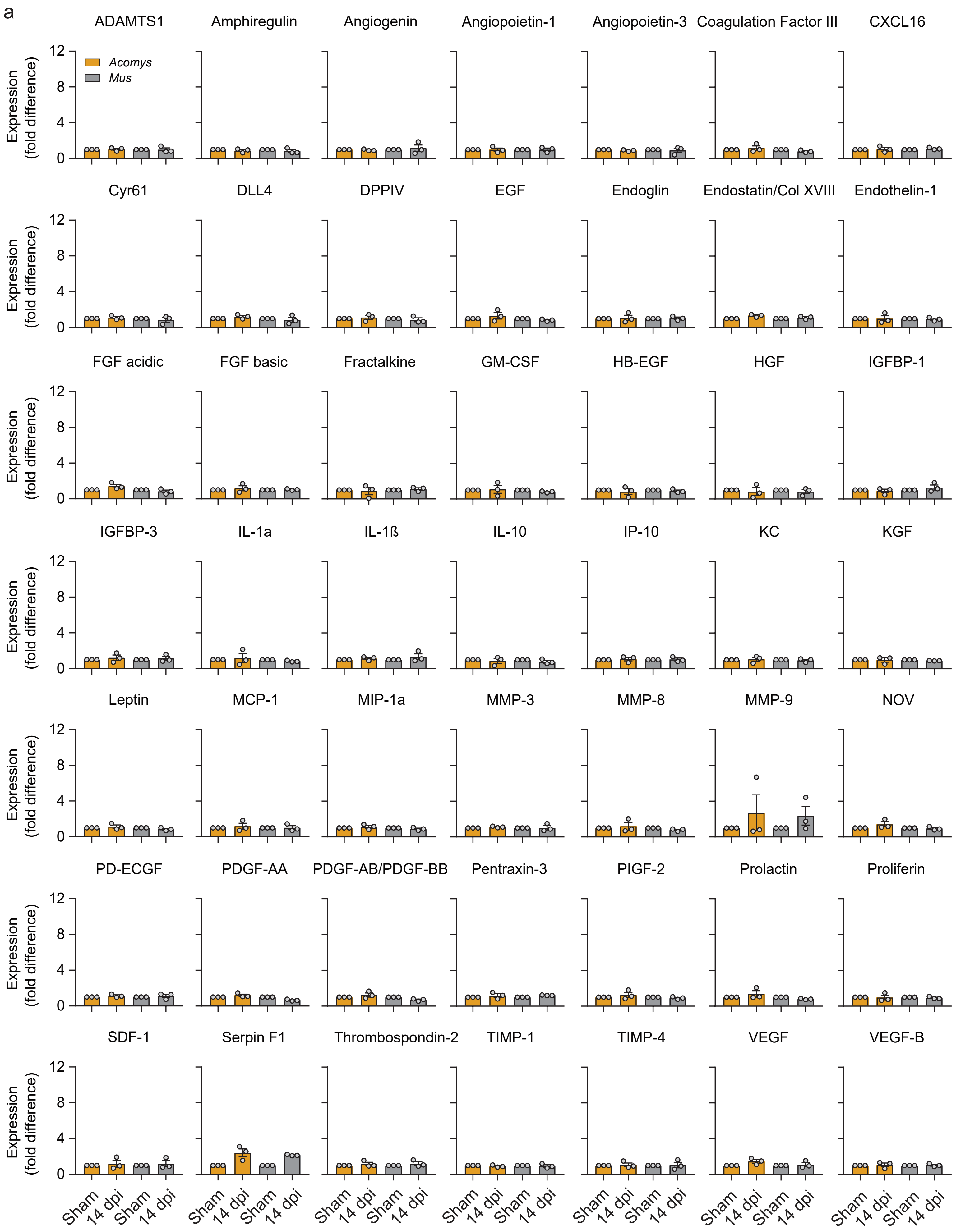

Supplementary figure 7. Proteomic profiling of infarcted hearts. (a) Remaining graphs not shown in Fig. 3d. Proteomic profiling of angiogenic proteins, obtained from whole heart ventricle homogenates (14 day post-MI and 14 day sham-controls). Samples are normalized to the sham-control (*Acomys* or *Mus*). All bar graphs represent the mean ± standard error of the mean (SEM).

Supplementary table 1

Figure 1e (Scar area)

Two-way mixed ANOVA result

	Effect	DFn	DFd	F	p	p<.05	ges
1	group	1	6	29.584	0.002	*	0.407
2	time	4	24	20.465	1.90E-07	*	0.746
3	group:time	4	24	4.969	0.005	*	0.416

Simple pairwise comparison (between groups)

	time	.y.	group1	group2	n1	n2	p	p.signif	p.adj	p.adj.signif
1	control	score	Acomys	Mus	8	4	0.927	ns	0.927	ns
2	dpi.004	score	Acomys	Mus	8	4	0.602	ns	0.602	ns
3	dpi.014	score	Acomys	Mus	8	4	0.195	ns	0.195	ns
4	dpi.042	score	Acomys	Mus	8	4	0.074	ns	0.074	ns
5	dpi.100	score	Acomys	Mus	8	4	0.00189	**	0.00189	**

Simple pairwise comparison (within groups)

	group	.y.	group1	group2	n1	n2	p.adj	p.adj.signif
1	Acomys	score	control	dpi.004	8	8	0.152	ns
2	Acomys	score	control	dpi.014	8	8	0.012	*
3	Acomys	score	control	dpi.042	8	8	0.478	ns
4	Acomys	score	control	dpi.100	8	8	0.52	ns
5	Acomys	score	dpi.004	dpi.014	8	8	0.524	ns
6	Acomys	score	dpi.004	dpi.042	8	8	0.28	ns
7	Acomys	score	dpi.004	dpi.100	8	8	1	ns
8	Acomys	score	dpi.014	dpi.042	8	8	1	ns
9	Acomys	score	dpi.014	dpi.100	8	8	1	ns
10	Acomys	score	dpi.042	dpi.100	8	8	1	ns
11	Mus	score	control	dpi.004	4	4	0.006	**
12	Mus	score	control	dpi.014	4	4	0.058	ns
13	Mus	score	control	dpi.042	4	4	0.202	ns
14	Mus	score	control	dpi.100	4	4	0.037	*
15	Mus	score	dpi.004	dpi.014	4	4	0.108	ns
16	Mus	score	dpi.004	dpi.042	4	4	1	ns
17	Mus	score	dpi.004	dpi.100	4	4	1	ns
18	Mus	score	dpi.014	dpi.042	4	4	1	ns
19	Mus	score	dpi.014	dpi.100	4	4	0.461	ns
20	Mus	score	dpi.042	dpi.100	4	4	0.122	ns

Figure 1f (Ejection fraction)

Two-way mixed ANOVA result

	Effect	DFn	DFd	F	p	p<.05	ges
1	group	1	27	305.914	2.96E-16	*	0.526
2	time	4	108	68.446	9.68E-29	*	0.696
3	group:time	4	108	13.981	3.18E-09	*	0.318

Simple pairwise comparison (between groups)

	time	.y.	group1	group2	n1	n2	p	p.signif	p.adj	p.adj.signif
1	control	score	Acomys	Mus	15	14	0.541	ns	0.541	ns
2	dpi.004	score	Acomys	Mus	15	14	0.00772	**	0.00772	**
3	dpi.014	score	Acomys	Mus	15	14	1.31E-05	****	1.31E-05	****
4	dpi.042	score	Acomys	Mus	15	14	3.33E-10	****	3.33E-10	****
5	dpi.100	score	Acomys	Mus	15	14	8.24E-11	****	8.24E-11	****

Simple pairwise comparison (within groups)

	group	.y.	group1	group2	n1	n2	p.adj	p.adj.signif
1	Acomys	score	control	dpi.004	15	15	0.002	**
2	Acomys	score	control	dpi.014	15	15	0.00027	***
3	Acomys	score	control	dpi.042	15	15	0.00036	***
4	Acomys	score	control	dpi.100	15	15	0.022	*
5	Acomys	score	dpi.004	dpi.014	15	15	1	ns
6	Acomys	score	dpi.004	dpi.042	15	15	1	ns
7	Acomys	score	dpi.004	dpi.100	15	15	0.319	ns
8	Acomys	score	dpi.014	dpi.042	15	15	1	ns
9	Acomys	score	dpi.014	dpi.100	15	15	0.071	ns
10	Acomys	score	dpi.042	dpi.100	15	15	1	ns
11	Mus	score	control	dpi.004	14	14	4.88E-09	****
12	Mus	score	control	dpi.014	14	14	2.94E-09	****
13	Mus	score	control	dpi.042	14	14	8.42E-09	****
14	Mus	score	control	dpi.100	14	14	7.60E-10	****
15	Mus	score	dpi.004	dpi.014	14	14	1	ns
16	Mus	score	dpi.004	dpi.042	14	14	0.07	ns
17	Mus	score	dpi.004	dpi.100	14	14	0.009	**
18	Mus	score	dpi.014	dpi.042	14	14	0.088	ns
19	Mus	score	dpi.014	dpi.100	14	14	0.044	*
20	Mus	score	dpi.042	dpi.100	14	14	1	ns

Figure 1f (Fractional shortening)

Two-way mixed ANOVA result

	Effect	DFn	DFd	F	p	p<.05	ges
1	group	1	27	178.382	2.06E-13	*	0.493
2	time	4	108	75.243	2.42E-30	*	0.704
3	group:time	4	108	11.094	1.41E-07	*	0.26

Simple pairwise comparison (between groups)

	time	.y.	group1	group2	n1	n2	p	p.signif	p.adj	p.adj.signif
1	control	score	Acomys	Mus	15	14	0.496	ns	0.496	ns
2	dpi.004	score	Acomys	Mus	15	14	0.00576	**	0.00576	**
3	dpi.014	score	Acomys	Mus	15	14	1.51E-05	****	1.51E-05	****
4	dpi.042	score	Acomys	Mus	15	14	4.42E-11	****	4.42E-11	****
5	dpi.100	score	Acomys	Mus	15	14	3.46E-10	****	3.46E-10	****

Simple pairwise comparison (within groups)

	group	.y.	group1	group2	n1	n2	p.adj	p.adj.signif
1	Acomys	score	control	dpi.004	15	15	0.00029	***
2	Acomys	score	control	dpi.014	15	15	0.004	**
3	Acomys	score	control	dpi.042	15	15	0.00057	***
4	Acomys	score	control	dpi.100	15	15	0.004	**
5	Acomys	score	dpi.004	dpi.014	15	15	1	ns
6	Acomys	score	dpi.004	dpi.042	15	15	0.236	ns
7	Acomys	score	dpi.004	dpi.100	15	15	0.179	ns
8	Acomys	score	dpi.014	dpi.042	15	15	1	ns
9	Acomys	score	dpi.014	dpi.100	15	15	1	ns
10	Acomys	score	dpi.042	dpi.100	15	15	1	ns
11	Mus	score	control	dpi.004	14	14	2.29E-07	****
12	Mus	score	control	dpi.014	14	14	1.11E-08	****
13	Mus	score	control	dpi.042	14	14	1.81E-08	****
14	Mus	score	control	dpi.100	14	14	2.57E-09	****
15	Mus	score	dpi.004	dpi.014	14	14	1	ns
16	Mus	score	dpi.004	dpi.042	14	14	0.199	ns
17	Mus	score	dpi.004	dpi.100	14	14	0.01	*
18	Mus	score	dpi.014	dpi.042	14	14	0.04	*
19	Mus	score	dpi.014	dpi.100	14	14	0.004	**
20	Mus	score	dpi.042	dpi.100	14	14	1	ns

Figure 1i (Q amplitude)

Two-way mixed ANOVA result

	Effect	DFn	DFd	F	p	p<.05	ges
1	group	1	27	102.925	1.04E-10	*	0.371
2	time	3	80.99	14.87	8.48E-08	*	0.318
3	group:time	3	80.99	9.809	1.37E-05	*	0.235

Simple pairwise comparison (between groups)

	time	.y.	group1	group2	n1	n2	p	p.signif	p.adj	p.adj.signif
1	control	score	Acomys	Mus	15	14	7.84E-06	****	7.84E-06	****
2	dpi.004	score	Acomys	Mus	15	14	0.0025	**	0.0025	**
3	dpi.014	score	Acomys	Mus	15	14	1.58E-05	****	1.58E-05	****
4	dpi.042	score	Acomys	Mus	15	14	0.00024	***	0.00024	***
5	dpi.100	score	Acomys	Mus	15	14	3.11E-08	****	3.11E-08	****

Simple pairwise comparison (within groups)

	group	.y.	group1	group2	n1	n2	p.adj	p.adj.signif
1	Acomys	score	control	dpi.004	15	15	0.622	ns
2	Acomys	score	control	dpi.014	15	15	1	ns
3	Acomys	score	control	dpi.042	15	15	0.088	ns
4	Acomys	score	control	dpi.100	15	15	0.942	ns
5	Acomys	score	dpi.004	dpi.014	15	15	1	ns
6	Acomys	score	dpi.004	dpi.042	15	15	1	ns
7	Acomys	score	dpi.004	dpi.100	15	15	1	ns
8	Acomys	score	dpi.014	dpi.042	15	15	1	ns
9	Acomys	score	dpi.014	dpi.100	15	15	1	ns
10	Acomys	score	dpi.042	dpi.100	15	15	1	ns
11	Mus	score	control	dpi.004	14	14	0.004	**
12	Mus	score	control	dpi.014	14	14	1.25E-05	****
13	Mus	score	control	dpi.042	14	14	0.00035	***
14	Mus	score	control	dpi.100	14	14	1.37E-06	****
15	Mus	score	dpi.004	dpi.014	14	14	1	ns
16	Mus	score	dpi.004	dpi.042	14	14	1	ns
17	Mus	score	dpi.004	dpi.100	14	14	1	ns
18	Mus	score	dpi.014	dpi.042	14	14	1	ns
19	Mus	score	dpi.014	dpi.100	14	14	1	ns
20	Mus	score	dpi.042	dpi.100	14	14	1	ns

Figure 1i (Q duration)

Two-way mixed ANOVA result

	Effect	DFn	DFd	F	p	p<.05	ges
1	group	1	27	127.224	1.01E-11	*	0.467
2	time	4	108	28.162	5.02E-16	*	0.459
3	group:time	4	108	18.356	1.57E-11	*	0.356

Simple pairwise comparison (between groups)

	time	.y.	group1	group2	n1	n2	p	p.signif	p.adj	p.adj.signif
1	control	score	Acomys	Mus	15	14	0.00018	***	0.00018	***
2	dpi.004	score	Acomys	Mus	15	14	0.00034	***	0.00034	***
3	dpi.014	score	Acomys	Mus	15	14	2.25E-10	****	2.25E-10	****
4	dpi.042	score	Acomys	Mus	15	14	1.25E-07	****	1.25E-07	****
5	dpi.100	score	Acomys	Mus	15	14	1.16E-05	****	1.16E-05	****

Simple pairwise comparison (within groups)

	group	.y.	group1	group2	n1	n2	p.adj	p.adj.signif
1	Acomys	score	control	dpi.004	15	15	0.013	*
2	Acomys	score	control	dpi.014	15	15	1	ns
3	Acomys	score	control	dpi.042	15	15	1	ns
4	Acomys	score	control	dpi.100	15	15	0.645	ns
5	Acomys	score	dpi.004	dpi.014	15	15	1	ns
6	Acomys	score	dpi.004	dpi.042	15	15	1	ns
7	Acomys	score	dpi.004	dpi.100	15	15	1	ns
8	Acomys	score	dpi.014	dpi.042	15	15	1	ns
9	Acomys	score	dpi.014	dpi.100	15	15	1	ns
10	Acomys	score	dpi.042	dpi.100	15	15	1	ns
11	Mus	score	control	dpi.004	14	14	2.95E-06	****
12	Mus	score	control	dpi.014	14	14	1.42E-08	****
13	Mus	score	control	dpi.042	14	14	6.77E-06	****
14	Mus	score	control	dpi.100	14	14	3.17E-05	****
15	Mus	score	dpi.004	dpi.014	14	14	0.647	ns
16	Mus	score	dpi.004	dpi.042	14	14	1	ns
17	Mus	score	dpi.004	dpi.100	14	14	1	ns
18	Mus	score	dpi.014	dpi.042	14	14	1	ns
19	Mus	score	dpi.014	dpi.100	14	14	1	ns
20	Mus	score	dpi.042	dpi.100	14	14	1	ns

Figure 1i (R amplitude)

Two-way mixed ANOVA result

	Effect	DFn	DFd	F	p	p<.05	ges
1	group	1	27	8.013	0.009	*	0.06
2	time	4	108	33.994	2.40E-18	*	0.497
3	group:time	4	108	2.005	0.099		0.055

Simple pairwise comparison (between groups)

	time	.y.	group1	group2	n1	n2	p	p.signif	p.adj	p.adj.signif
1	control	score	Acomys	Mus	15	14	0.404	ns	0.404	ns
2	dpi.004	score	Acomys	Mus	15	14	0.0325	*	0.0325	*
3	dpi.014	score	Acomys	Mus	15	14	0.00742	**	0.00742	**
4	dpi.042	score	Acomys	Mus	15	14	0.479	ns	0.479	ns
5	dpi.100	score	Acomys	Mus	15	14	0.799	ns	0.799	ns

Simple pairwise comparison (within groups)

	group	.y.	group1	group2	n1	n2	p.adj	p.adj.signif
1	Acomys	score	control	dpi.004	15	15	9.91E-05	****
2	Acomys	score	control	dpi.014	15	15	0.001	**
3	Acomys	score	control	dpi.042	15	15	3.94E-05	****
4	Acomys	score	control	dpi.100	15	15	0.00056	***
5	Acomys	score	dpi.004	dpi.014	15	15	0.836	ns
6	Acomys	score	dpi.004	dpi.042	15	15	1	ns
7	Acomys	score	dpi.004	dpi.100	15	15	1	ns
8	Acomys	score	dpi.014	dpi.042	15	15	1	ns
9	Acomys	score	dpi.014	dpi.100	15	15	0.411	ns
10	Acomys	score	dpi.042	dpi.100	15	15	1	ns
11	Mus	score	control	dpi.004	14	14	6.26E-06	****
12	Mus	score	control	dpi.014	14	14	8.35E-06	****
13	Mus	score	control	dpi.042	14	14	2.22E-05	****
14	Mus	score	control	dpi.100	14	14	0.002	**
15	Mus	score	dpi.004	dpi.014	14	14	1	ns
16	Mus	score	dpi.004	dpi.042	14	14	0.889	ns
17	Mus	score	dpi.004	dpi.100	14	14	1	ns
18	Mus	score	dpi.014	dpi.042	14	14	1	ns
19	Mus	score	dpi.014	dpi.100	14	14	1	ns
20	Mus	score	dpi.042	dpi.100	14	14	1	ns

Figure 1i (R duration)

Two-way mixed ANOVA result

	Effect	DFn	DFd	F	p	p<.05	ges
1	group	1	27	130.012	7.92E-12	*	0.415
2	time	2.98	80.56	5.157	0.003	*	0.14
3	group:time	2.98	80.56	6.989	0.00032	*	0.181

Simple pairwise comparison (between groups)

	time	.y.	group1	group2	n1	n2	p	p.signif	p.adj	p.adj.signif
1	control	score	Acomys	Mus	15	14	0.979	ns	0.979	ns
2	dpi.004	score	Acomys	Mus	15	14	0.00487	**	0.00487	**
3	dpi.014	score	Acomys	Mus	15	14	4.60E-06	****	4.60E-06	****
4	dpi.042	score	Acomys	Mus	15	14	2.70E-07	****	2.70E-07	****
5	dpi.100	score	Acomys	Mus	15	14	7.22E-05	****	7.22E-05	****

Simple pairwise comparison (within groups)

	group	.y.	group1	group2	n1	n2	p.adj	p.adj.signif
1	Acomys	score	control	dpi.004	15	15	0.418	ns
2	Acomys	score	control	dpi.014	15	15	1	ns
3	Acomys	score	control	dpi.042	15	15	0.753	ns
4	Acomys	score	control	dpi.100	15	15	1	ns
5	Acomys	score	dpi.004	dpi.014	15	15	0.291	ns
6	Acomys	score	dpi.004	dpi.042	15	15	0.231	ns
7	Acomys	score	dpi.004	dpi.100	15	15	0.175	ns
8	Acomys	score	dpi.014	dpi.042	15	15	1	ns
9	Acomys	score	dpi.014	dpi.100	15	15	1	ns
10	Acomys	score	dpi.042	dpi.100	15	15	1	ns
11	Mus	score	control	dpi.004	14	14	0.005	**
12	Mus	score	control	dpi.014	14	14	0.0008	***
13	Mus	score	control	dpi.042	14	14	0.00011	***
14	Mus	score	control	dpi.100	14	14	0.006	**
15	Mus	score	dpi.004	dpi.014	14	14	1	ns
16	Mus	score	dpi.004	dpi.042	14	14	1	ns
17	Mus	score	dpi.004	dpi.100	14	14	1	ns
18	Mus	score	dpi.014	dpi.042	14	14	1	ns
19	Mus	score	dpi.014	dpi.100	14	14	1	ns
20	Mus	score	dpi.042	dpi.100	14	14	1	ns

Figure 1i (S amplitude)

Two-way mixed ANOVA result

	Effect	DFn	DFd	F	p	p<.05	ges
1	group	1	27	224.806	1.30E-14	*	0.534
2	time	4	108	4.175	0.003	*	0.118
3	group:time	4	108	1.054	0.383		0.033

Simple pairwise comparison (between groups)

	time	.y.	group1	group2	n1	n2	p	p.signif	p.adj	p.adj.signif
1	control	score	Acomys	Mus	15	14	0.404	ns	0.404	ns
2	dpi.004	score	Acomys	Mus	15	14	0.0325	*	0.0325	*
3	dpi.014	score	Acomys	Mus	15	14	0.00742	**	0.00742	**
4	dpi.042	score	Acomys	Mus	15	14	0.479	ns	0.479	ns
5	dpi.100	score	Acomys	Mus	15	14	0.799	ns	0.799	ns

Simple pairwise comparison (within groups)

	group	.y.	group1	group2	n1	n2	p.adj	p.adj.signif
1	Acomys	score	control	dpi.004	15	15	0.954	ns
2	Acomys	score	control	dpi.014	15	15	1	ns
3	Acomys	score	control	dpi.042	15	15	0.024	*
4	Acomys	score	control	dpi.100	15	15	0.724	ns
5	Acomys	score	dpi.004	dpi.014	15	15	1	ns
6	Acomys	score	dpi.004	dpi.042	15	15	1	ns
7	Acomys	score	dpi.004	dpi.100	15	15	1	ns
8	Acomys	score	dpi.014	dpi.042	15	15	1	ns
9	Acomys	score	dpi.014	dpi.100	15	15	1	ns
10	Acomys	score	dpi.042	dpi.100	15	15	1	ns
11	Mus	score	control	dpi.004	14	14	0.42	ns
12	Mus	score	control	dpi.014	14	14	1	ns
13	Mus	score	control	dpi.042	14	14	0.136	ns
14	Mus	score	control	dpi.100	14	14	0.234	ns
15	Mus	score	dpi.004	dpi.014	14	14	1	ns
16	Mus	score	dpi.004	dpi.042	14	14	1	ns
17	Mus	score	dpi.004	dpi.100	14	14	1	ns
18	Mus	score	dpi.014	dpi.042	14	14	1	ns
19	Mus	score	dpi.014	dpi.100	14	14	1	ns
20	Mus	score	dpi.042	dpi.100	14	14	1	ns

Figure 1i (S duration)

Two-way mixed ANOVA result

	Effect	DFn	DFd	F	p	p<.05	ges
1	group	1	27	28.611	1.19E-05	*	0.103
2	time	4	108	3.191	0.016	*	0.095
3	group:time	4	108	2.448	0.051		0.075

Simple pairwise comparison (between groups)

	time	.y.	group1	group2	n1	n2	p	p.signif	p.adj	p.adj.signif
1	control	score	Acomys	Mus	15	14	0.624	ns	0.624	ns
2	dpi.004	score	Acomys	Mus	15	14	0.483	ns	0.483	ns
3	dpi.014	score	Acomys	Mus	15	14	0.192	ns	0.192	ns
4	dpi.042	score	Acomys	Mus	15	14	0.00295	**	0.00295	**
5	dpi.100	score	Acomys	Mus	15	14	0.00024	***	0.00024	***

Simple pairwise comparison (within groups)

	group	.y.	group1	group2	n1	n2	p.adj	p.adj.signif
1	Acomys	score	control	dpi.004	15	15	0.997	ns
2	Acomys	score	control	dpi.014	15	15	1	ns
3	Acomys	score	control	dpi.042	15	15	0.001	**
4	Acomys	score	control	dpi.100	15	15	0.003	**
5	Acomys	score	dpi.004	dpi.014	15	15	1	ns
6	Acomys	score	dpi.004	dpi.042	15	15	0.103	ns
7	Acomys	score	dpi.004	dpi.100	15	15	0.23	ns
8	Acomys	score	dpi.014	dpi.042	15	15	0.357	ns
9	Acomys	score	dpi.014	dpi.100	15	15	1	ns
10	Acomys	score	dpi.042	dpi.100	15	15	1	ns
11	Mus	score	control	dpi.004	14	14	1	ns
12	Mus	score	control	dpi.014	14	14	1	ns
13	Mus	score	control	dpi.042	14	14	1	ns
14	Mus	score	control	dpi.100	14	14	1	ns
15	Mus	score	dpi.004	dpi.014	14	14	1	ns
16	Mus	score	dpi.004	dpi.042	14	14	1	ns
17	Mus	score	dpi.004	dpi.100	14	14	1	ns
18	Mus	score	dpi.014	dpi.042	14	14	1	ns
19	Mus	score	dpi.014	dpi.100	14	14	1	ns
20	Mus	score	dpi.042	dpi.100	14	14	1	ns

Figure 2b (TUNEL assay)

Two-way mixed ANOVA result

	Effect	DFn	DFd	F	p	p<.05	ges
1	group	1	6	0.11	0.751		0.004
2	time	1.09	6.54	33.583	0.00076	*	0.811
3	group:time	1.09	6.54	0.212	0.681		0.026

Simple pairwise comparison (between groups)

	time	.y.	group1	group2	n1	n2	p	p.signif	p.adj	p.adj.signif
1	dpi.001	score	Acomys	Mus	5	4	0.935	ns	0.935	ns
2	dpi.004	score	Acomys	Mus	5	4	0.2	ns	0.2	ns
3	dpi.014	score	Acomys	Mus	5	4	0.732	ns	0.732	ns
4	dpi.042	score	Acomys	Mus	5	4	0.157	ns	0.157	ns
5	dpi.100	score	Acomys	Mus	5	4	0.0767	ns	0.0767	ns

Simple pairwise comparison (within groups)

	group	.y.	group1	group2	n1	n2	p.adj	p.adj.signif
1	Acomys	score	control	dpi.001	5	5	0.364	ns
2	Acomys	score	control	dpi.004	5	5	0.774	ns
3	Acomys	score	control	dpi.014	5	5	0.762	ns
4	Acomys	score	control	dpi.042	5	5	0.951	ns
5	Acomys	score	control	dpi.100	5	5	0.064	ns
6	Acomys	score	dpi.001	dpi.004	5	5	0.397	ns
7	Acomys	score	dpi.001	dpi.014	5	5	0.382	ns
8	Acomys	score	dpi.001	dpi.042	5	5	0.366	ns
9	Acomys	score	dpi.001	dpi.100	5	5	0.411	ns
10	Acomys	score	dpi.004	dpi.014	5	5	1	ns
11	Acomys	score	dpi.004	dpi.042	5	5	1	ns
12	Acomys	score	dpi.004	dpi.100	5	5	1	ns
13	Acomys	score	dpi.014	dpi.042	5	5	1	ns
14	Acomys	score	dpi.014	dpi.100	5	5	1	ns
15	Acomys	score	dpi.042	dpi.100	5	5	1	ns
16	Mus	score	control	dpi.001	4	4	0.284	ns
17	Mus	score	control	dpi.004	4	4	0.237	ns
18	Mus	score	control	dpi.014	4	4	0.566	ns
19	Mus	score	control	dpi.042	4	4	1	ns
20	Mus	score	control	dpi.100	4	4	0.273	ns
21	Mus	score	dpi.001	dpi.004	4	4	0.316	ns
22	Mus	score	dpi.001	dpi.014	4	4	0.394	ns
23	Mus	score	dpi.001	dpi.042	4	4	0.272	ns
24	Mus	score	dpi.001	dpi.100	4	4	0.618	ns
25	Mus	score	dpi.004	dpi.014	4	4	1	ns
26	Mus	score	dpi.004	dpi.042	4	4	0.532	ns
27	Mus	score	dpi.004	dpi.100	4	4	1	ns
28	Mus	score	dpi.014	dpi.042	4	4	1	ns
29	Mus	score	dpi.014	dpi.100	4	4	0.848	ns
30	Mus	score	dpi.042	dpi.100	4	4	0.93	ns

Figure 2c (Heart body weight ratio)

Two-way mixed ANOVA result

	Effect	DFn	DFd	F	p	p<.05	ges
1	group	1	18	168.623	1.40E-10	*	0.791
2	time	2.15	38.74	32.155	3.03E-09	*	0.515
3	group:time	2.15	38.74	6.678	0.003	*	0.181

Simple pairwise comparison (between groups)

	time	.y.	group1	group2	n1	n2	p	p.signif	p.adj	p.adj.signif
1	control	score	Acomys	Mus	10	10	3.20E-10	****	3.20E-10	****
2	dpi.014	score	Acomys	Mus	10	10	8.23E-10	****	8.23E-10	****
3	dpi.042	score	Acomys	Mus	10	10	3.99E-07	****	3.99E-07	****
4	dpi.100	score	Acomys	Mus	10	10	5.27E-07	****	5.27E-07	****

Simple pairwise comparison (within groups)

	group	.y.	group1	group2	n1	n2	p.adj	p.adj.signif
1	Acomys	score	control	dpi.014	10	10	0.035	*
2	Acomys	score	control	dpi.042	10	10	0.001	**
3	Acomys	score	control	dpi.100	10	10	0.001	**
4	Acomys	score	dpi.014	dpi.042	10	10	0.024	*
5	Acomys	score	dpi.014	dpi.100	10	10	0.395	ns
6	Acomys	score	dpi.042	dpi.100	10	10	1	ns
7	Mus	score	control	dpi.014	10	10	0.00059	***
8	Mus	score	control	dpi.042	10	10	0.00011	***
9	Mus	score	control	dpi.100	10	10	0.00067	***
10	Mus	score	dpi.014	dpi.042	10	10	0.073	ns
11	Mus	score	dpi.014	dpi.100	10	10	0.29	ns
12	Mus	score	dpi.042	dpi.100	10	10	1	ns

Figure 2d (Heart size)

Two-way mixed ANOVA result

	Effect	DFn	DFd	F	p	p<.05	ges
1	group	1	6	15.586	0.008	*	0.61
2	time	3	18	34.966	1.02E-07	*	0.699
3	group:time	3	18	9.785	0.00047	*	0.394

Simple pairwise comparison (between groups)

	time	.y.	group1	group2	n1	n2	p	p.signif	p.adj	p.adj.signif
1	control	score	Acomys	Mus	5	4	0.672	ns	0.672	ns
2	dpi.014	score	Acomys	Mus	5	4	0.0591	ns	0.0591	ns
3	dpi.042	score	Acomys	Mus	5	4	0.0425	*	0.0425	*
4	dpi.100	score	Acomys	Mus	5	4	0.00053	***	0.00053	***

Simple pairwise comparison (within groups)

	group	.y.	group1	group2	n1	n2	p.adj	p.adj.signif
1	Acomys	score	control	dpi.014	5	5	1	ns
2	Acomys	score	control	dpi.042	5	5	0.357	ns
3	Acomys	score	control	dpi.100	5	5	0.426	ns
4	Acomys	score	dpi.014	dpi.042	5	5	0.05	*
5	Acomys	score	dpi.014	dpi.100	5	5	0.196	ns
6	Acomys	score	dpi.042	dpi.100	5	5	1	ns
7	Mus	score	control	dpi.014	4	4	0.726	ns
8	Mus	score	control	dpi.042	4	4	0.032	*
9	Mus	score	control	dpi.100	4	4	0.002	**
10	Mus	score	dpi.014	dpi.042	4	4	0.563	ns
11	Mus	score	dpi.014	dpi.100	4	4	0.031	*
12	Mus	score	dpi.042	dpi.100	4	4	0.558	ns

Figure 2e (Heart wall thickness)

Two-way mixed ANOVA result

	Effect	DFn	DFd	F	p	p<.05	ges
1	group	1	6	4.755	0.072		0.201
2	time	1.23	7.35	155.302	2.25E-06	*	0.946
3	group:time	1.23	7.35	1.206	0.322		0.121

Simple pairwise comparison (between groups)

	time	.y.	group1	group2	n1	n2	p	p.signif	p.adj	p.adj.signif
1	control	score	Acomys	Mus	6	4	0.862	ns	0.862	ns
2	dpi.014	score	Acomys	Mus	6	4	0.0051	**	0.0051	**
3	dpi.042	score	Acomys	Mus	6	4	0.0101	*	0.0101	*
4	dpi.100	score	Acomys	Mus	6	4	0.00959	**	0.00959	**

Simple pairwise comparison (within groups)

	group	.y.	group1	group2	n1	n2	p.adj	p.adj.signif
1	Acomys	score	control	dpi.014	4	4	0.042	*
2	Acomys	score	control	dpi.042	4	4	0.015	*
3	Acomys	score	control	dpi.100	4	4	0.04	*
4	Acomys	score	dpi.014	dpi.042	4	4	1	ns
5	Acomys	score	dpi.014	dpi.100	4	4	0.218	ns
6	Acomys	score	dpi.042	dpi.100	4	4	1	ns
7	Mus	score	control	dpi.014	4	4	0.009	**
8	Mus	score	control	dpi.042	4	4	0.006	**
9	Mus	score	control	dpi.100	4	4	0.006	**
10	Mus	score	dpi.014	dpi.042	4	4	0.185	ns
11	Mus	score	dpi.014	dpi.100	4	4	0.067	ns
12	Mus	score	dpi.042	dpi.100	4	4	0.672	ns

Figure 2f (Nppa RT-qPCR)

Two-way mixed ANOVA result

	Effect	DFn	DFd	F	p	p<.05	ges
1	group	1	7	303.848	5.03E-07	*	0.655
2	time	1.28	8.96	7.396	0.019	*	0.503
3	group:time	1.28	8.96	9.716	0.01	*	0.57

Simple pairwise comparison (between groups)

	time	.y.	group1	group2	n1	n2	p	p.signif	p.adj	p.adj.signif
1	control	score	Acomys	Mus	6	8	1	ns	1	ns
2	dpi.004	score	Acomys	Mus	6	8	0.00891	**	0.00891	**
3	dpi.014	score	Acomys	Mus	6	8	0.00861	**	0.00861	**
4	dpi.042	score	Acomys	Mus	6	8	0.0311	*	0.0311	*
5	dpi.100	score	Acomys	Mus	6	8	0.00367	**	0.00367	**

Simple pairwise comparison (within groups)

	group	.y.	group1	group2	n1	n2	p.adj	p.adj.signif
1	Acomys	score	control	dpi.004	6	6	0.569	ns
2	Acomys	score	control	dpi.014	6	6	1	ns
3	Acomys	score	control	dpi.042	6	6	0.485	ns
4	Acomys	score	control	dpi.100	6	6	1	ns
5	Acomys	score	dpi.004	dpi.014	6	6	1	ns
6	Acomys	score	dpi.004	dpi.042	6	6	1	ns
7	Acomys	score	dpi.004	dpi.100	6	6	1	ns
8	Acomys	score	dpi.014	dpi.042	6	6	1	ns
9	Acomys	score	dpi.014	dpi.100	6	6	1	ns
10	Acomys	score	dpi.042	dpi.100	6	6	1	ns
11	Mus	score	control	dpi.004	8	8	0.165	ns
12	Mus	score	control	dpi.014	8	8	0.246	ns
13	Mus	score	control	dpi.042	8	8	0.282	ns
14	Mus	score	control	dpi.100	8	8	0.75	ns
15	Mus	score	dpi.004	dpi.014	8	8	1	ns
16	Mus	score	dpi.004	dpi.042	8	8	1	ns
17	Mus	score	dpi.004	dpi.100	8	8	1	ns
18	Mus	score	dpi.014	dpi.042	8	8	0.883	ns
19	Mus	score	dpi.014	dpi.100	8	8	0.949	ns
20	Mus	score	dpi.042	dpi.100	8	8	1	ns

Figure 2f (Myh6 RT-qPCR)

Two-way mixed ANOVA result

	Effect	DFn	DFd	F	p	p<.05	ges
1	group	1	7	7.05	0.033	*	0.234
2	time	4	28	5.312	0.003	*	0.346
3	group:time	4	28	12.3	6.76E-06	*	0.55

Simple pairwise comparison (between groups)

	time	.y.	group1	group2	n1	n2	p	p.signif	p.adj	p.adj.signif
1	control	score	Acomys	Mus	6	8	1	ns	1	ns
2	dpi.004	score	Acomys	Mus	6	8	0.0144	*	0.0144	*
3	dpi.014	score	Acomys	Mus	6	8	0.00022	***	0.00022	***
4	dpi.042	score	Acomys	Mus	6	8	0.331	ns	0.331	ns
5	dpi.100	score	Acomys	Mus	6	8	0.837	ns	0.837	ns

Simple pairwise comparison (within groups)

	group	.y.	group1	group2	n1	n2	p.adj	p.adj.signif
1	Acomys	score	control	dpi.004	6	6	0.042	*
2	Acomys	score	control	dpi.014	6	6	0.591	ns
3	Acomys	score	control	dpi.042	6	6	0.74	ns
4	Acomys	score	control	dpi.100	6	6	0.285	ns
5	Acomys	score	dpi.004	dpi.014	6	6	0.024	*
6	Acomys	score	dpi.004	dpi.042	6	6	0.497	ns
7	Acomys	score	dpi.004	dpi.100	6	6	0.12	ns
8	Acomys	score	dpi.014	dpi.042	6	6	1	ns
9	Acomys	score	dpi.014	dpi.100	6	6	1	ns
10	Acomys	score	dpi.042	dpi.100	6	6	1	ns
11	Mus	score	control	dpi.004	8	8	1	ns
12	Mus	score	control	dpi.014	8	8	0.046	*
13	Mus	score	control	dpi.042	8	8	1	ns
14	Mus	score	control	dpi.100	8	8	1	ns
15	Mus	score	dpi.004	dpi.014	8	8	0.002	**
16	Mus	score	dpi.004	dpi.042	8	8	1	ns
17	Mus	score	dpi.004	dpi.100	8	8	1	ns
18	Mus	score	dpi.014	dpi.042	8	8	0.042	*
19	Mus	score	dpi.014	dpi.100	8	8	0.458	ns
20	Mus	score	dpi.042	dpi.100	8	8	1	ns

Figure 2f (Myh7 RT-qPCR)

Two-way mixed ANOVA result

	Effect	DFn	DFd	F	p	p<.05	ges
1	group	1	7	55.357	0.00014	*	0.588
2	time	1.41	9.85	3.304	0.091		0.279
3	group:time	1.41	9.85	6.399	0.023	*	0.428

Simple pairwise comparison (between groups)

	time	.y.	group1	group2	n1	n2	p	p.signif	p.adj	p.adj.signif
1	control	score	Acomys	Mus	6	8	1	ns	1	ns
2	dpi.004	score	Acomys	Mus	6	8	0.00129	**	0.00129	**
3	dpi.014	score	Acomys	Mus	6	8	0.00915	**	0.00915	**
4	dpi.042	score	Acomys	Mus	6	8	0.0566	ns	0.0566	ns
5	dpi.100	score	Acomys	Mus	6	8	0.00083	***	0.00083	***

Simple pairwise comparison (within groups)

	group	.y.	group1	group2	n1	n2	p.adj	p.adj.signif
1	Acomys	score	control	dpi.004	6	6	1	ns
2	Acomys	score	control	dpi.014	6	6	1	ns
3	Acomys	score	control	dpi.042	6	6	1	ns
4	Acomys	score	control	dpi.100	6	6	1	ns
5	Acomys	score	dpi.004	dpi.014	6	6	1	ns
6	Acomys	score	dpi.004	dpi.042	6	6	1	ns
7	Acomys	score	dpi.004	dpi.100	6	6	1	ns
8	Acomys	score	dpi.014	dpi.042	6	6	1	ns
9	Acomys	score	dpi.014	dpi.100	6	6	1	ns
10	Acomys	score	dpi.042	dpi.100	6	6	1	ns
11	Mus	score	control	dpi.004	8	8	0.043	*
12	Mus	score	control	dpi.014	8	8	0.442	ns
13	Mus	score	control	dpi.042	8	8	0.701	ns
14	Mus	score	control	dpi.100	8	8	0.45	ns
15	Mus	score	dpi.004	dpi.014	8	8	1	ns
16	Mus	score	dpi.004	dpi.042	8	8	1	ns
17	Mus	score	dpi.004	dpi.100	8	8	1	ns
18	Mus	score	dpi.014	dpi.042	8	8	1	ns
19	Mus	score	dpi.014	dpi.100	8	8	1	ns
20	Mus	score	dpi.042	dpi.100	8	8	1	ns

Figure 2f (Myh10 RT-qPCR)

Two-way mixed ANOVA result

	Effect	DFn	DFd	F	p	p<.05	ges
1	group	1	7	52.874	0.00017	*	0.438
2	time	4	28	6.98	0.0005	*	0.472
3	group:time	4	28	10.489	2.54E-05	*	0.573

Simple pairwise comparison (between groups)

	time	.y.	group1	group2	n1	n2	p	p.signif	p.adj	p.adj.signif
1	control	score	Acomys	Mus	6	8	1	ns	1	ns
2	dpi.004	score	Acomys	Mus	6	8	2.00E-04	***	2.00E-04	***
3	dpi.014	score	Acomys	Mus	6	8	0.0885	ns	0.0885	ns
4	dpi.042	score	Acomys	Mus	6	8	0.997	ns	0.997	ns
5	dpi.100	score	Acomys	Mus	6	8	0.89	ns	0.89	ns

Simple pairwise comparison (within groups)

	group	.y.	group1	group2	n1	n2	p.adj	p.adj.signif
1	Acomys	score	control	dpi.004	6	6	0.56	ns
2	Acomys	score	control	dpi.014	6	6	1	ns
3	Acomys	score	control	dpi.042	6	6	0.561	ns
4	Acomys	score	control	dpi.100	6	6	0.36	ns
5	Acomys	score	dpi.004	dpi.014	6	6	1	ns
6	Acomys	score	dpi.004	dpi.042	6	6	1	ns
7	Acomys	score	dpi.004	dpi.100	6	6	0.993	ns
8	Acomys	score	dpi.014	dpi.042	6	6	1	ns
9	Acomys	score	dpi.014	dpi.100	6	6	1	ns
10	Acomys	score	dpi.042	dpi.100	6	6	1	ns
11	Mus	score	control	dpi.004	8	8	0.007	**
12	Mus	score	control	dpi.014	8	8	0.313	ns
13	Mus	score	control	dpi.042	8	8	1	ns
14	Mus	score	control	dpi.100	8	8	1	ns
15	Mus	score	dpi.004	dpi.014	8	8	0.664	ns
16	Mus	score	dpi.004	dpi.042	8	8	0.083	ns
17	Mus	score	dpi.004	dpi.100	8	8	0.544	ns
18	Mus	score	dpi.014	dpi.042	8	8	1	ns
19	Mus	score	dpi.014	dpi.100	8	8	0.656	ns
20	Mus	score	dpi.042	dpi.100	8	8	1	ns

Figure 2f (Pfkp RT-qPCR)

Two-way mixed ANOVA result

	Effect	DFn	DFd	F	p	p<.05	ges
1	group	1	7	41.503	0.00035	*	0.461
2	time	4	28	4.372	0.007	*	0.348
3	group:time	4	28	4.57	0.006	*	0.358

Simple pairwise comparison (between groups)

	time	.y.	group1	group2	n1	n2	p	p.signif	p.adj	p.adj.signif
1	control	score	Acomys	Mus	6	8	1	ns	1	ns
2	dpi.004	score	Acomys	Mus	6	8	0.00431	**	0.00431	**
3	dpi.014	score	Acomys	Mus	6	8	0.00863	**	0.00863	**
4	dpi.042	score	Acomys	Mus	6	8	0.313	ns	0.313	ns
5	dpi.100	score	Acomys	Mus	6	8	0.253	ns	0.253	ns

Simple pairwise comparison (within groups)

	group	.y.	group1	group2	n1	n2	p.adj	p.adj.signif
1	Acomys	score	control	dpi.004	5	5	0.154	ns
2	Acomys	score	control	dpi.014	5	5	0.034	*
3	Acomys	score	control	dpi.100	5	5	0.011	*
4	Acomys	score	dpi.004	dpi.014	5	5	0.4	ns
5	Acomys	score	dpi.004	dpi.100	5	5	0.484	ns
6	Acomys	score	dpi.014	dpi.100	5	5	1	ns
7	Mus	score	control	dpi.004	4	4	0.00091	***
8	Mus	score	control	dpi.014	4	4	0.011	*
9	Mus	score	control	dpi.100	4	4	0.009	**
10	Mus	score	dpi.004	dpi.014	4	4	0.086	ns
11	Mus	score	dpi.004	dpi.100	4	4	0.011	*
12	Mus	score	dpi.014	dpi.100	4	4	1	ns

Figure 2f (Prnp RT-qPCR)

Two-way mixed ANOVA result

	Effect	DFn	DFd	F	p	p<.05	ges
1	group	1	7	90.278	2.99E-05	*	0.572
2	time	4	28	1.044	0.402		0.118
3	group:time	4	28	5.874	0.001	*	0.429

Simple pairwise comparison (between groups)

	time	.y.	group1	group2	n1	n2	p	p.signif	p.adj	p.adj.signif
1	control	score	Acomys	Mus	6	8	1	ns	1	ns
2	dpi.004	score	Acomys	Mus	6	8	0.00025	***	0.00025	***
3	dpi.014	score	Acomys	Mus	6	8	0.00191	**	0.00191	**
4	dpi.042	score	Acomys	Mus	6	8	0.00633	**	0.00633	**
5	dpi.100	score	Acomys	Mus	6	8	0.0731	ns	0.0731	ns

Simple pairwise comparison (within groups)

	group	.y.	group1	group2	n1	n2	p.adj	p.adj.signif
1	Acomys	score	control	dpi.004	6	6	1	ns
2	Acomys	score	control	dpi.014	6	6	1	ns
3	Acomys	score	control	dpi.042	6	6	1	ns
4	Acomys	score	control	dpi.100	6	6	1	ns
5	Acomys	score	dpi.004	dpi.014	6	6	1	ns
6	Acomys	score	dpi.004	dpi.042	6	6	1	ns
7	Acomys	score	dpi.004	dpi.100	6	6	0.493	ns
8	Acomys	score	dpi.014	dpi.042	6	6	1	ns
9	Acomys	score	dpi.014	dpi.100	6	6	1	ns
10	Acomys	score	dpi.042	dpi.100	6	6	1	ns
11	Mus	score	control	dpi.004	8	8	0.024	*
12	Mus	score	control	dpi.014	8	8	0.329	ns
13	Mus	score	control	dpi.042	8	8	0.371	ns
14	Mus	score	control	dpi.100	8	8	1	ns
15	Mus	score	dpi.004	dpi.014	8	8	1	ns
16	Mus	score	dpi.004	dpi.042	8	8	1	ns
17	Mus	score	dpi.004	dpi.100	8	8	0.011	*
18	Mus	score	dpi.014	dpi.042	8	8	1	ns
19	Mus	score	dpi.014	dpi.100	8	8	1	ns
20	Mus	score	dpi.042	dpi.100	8	8	1	ns

Figure 2f (Sorbs2 RT-qPCR)

Two-way mixed ANOVA result

	Effect	DFn	DFd	F	p	p<.05	ges
1	group	1	7	63.968	9.13E-05	*	0.525
2	time	4	28	0.86	0.5		0.098
3	group:time	4	28	8.2	0.00017	*	0.507

Simple pairwise comparison (between groups)

	time	.y.	group1	group2	n1	n2	p	p.signif	p.adj	p.adj.signif
1	control	score	Acomys	Mus	6	8	1	ns	1	ns
2	dpi.004	score	Acomys	Mus	6	8	0.00027	***	0.00027	***
3	dpi.014	score	Acomys	Mus	6	8	2.36E-05	****	2.36E-05	****
4	dpi.042	score	Acomys	Mus	6	8	0.0858	ns	0.0858	ns
5	dpi.100	score	Acomys	Mus	6	8	0.569	ns	0.569	ns

Simple pairwise comparison (within groups)

	group	.y.	group1	group2	n1	n2	p.adj	p.adj.signif
1	Acomys	score	control	dpi.004	6	6	1	ns
2	Acomys	score	control	dpi.014	6	6	0.184	ns
3	Acomys	score	control	dpi.042	6	6	1	ns
4	Acomys	score	control	dpi.100	6	6	1	ns
5	Acomys	score	dpi.004	dpi.014	6	6	1	ns
6	Acomys	score	dpi.004	dpi.042	6	6	1	ns
7	Acomys	score	dpi.004	dpi.100	6	6	1	ns
8	Acomys	score	dpi.014	dpi.042	6	6	1	ns
9	Acomys	score	dpi.014	dpi.100	6	6	0.129	ns
10	Acomys	score	dpi.042	dpi.100	6	6	1	ns
11	Mus	score	control	dpi.004	8	8	0.003	**
12	Mus	score	control	dpi.014	8	8	0.041	*
13	Mus	score	control	dpi.042	8	8	1	ns
14	Mus	score	control	dpi.100	8	8	1	ns
15	Mus	score	dpi.004	dpi.014	8	8	1	ns
16	Mus	score	dpi.004	dpi.042	8	8	0.897	ns
17	Mus	score	dpi.004	dpi.100	8	8	0.00029	***
18	Mus	score	dpi.014	dpi.042	8	8	0.461	ns
19	Mus	score	dpi.014	dpi.100	8	8	1	ns
20	Mus	score	dpi.042	dpi.100	8	8	1	ns

Figure 2f (Mafk RT-qPCR)

Two-way mixed ANOVA result

	Effect	DFn	DFd	F	p	p<.05	ges
1	group	1	7	0.368	0.563		0.011
2	time	2.21	15.46	6.274	0.009	*	0.413
3	group:time	2.21	15.46	4.203	0.032	*	0.321

Simple pairwise comparison (between groups)

	time	.y.	group1	group2	n1	n2	p	p.signif	p.adj	p.adj.signif
1	control	score	Acomys	Mus	6	8	1	ns	1	ns
2	dpi.004	score	Acomys	Mus	6	8	0.0244	*	0.0244	*
3	dpi.014	score	Acomys	Mus	6	8	0.00047	***	0.00047	***
4	dpi.042	score	Acomys	Mus	6	8	0.0115	*	0.0115	*
5	dpi.100	score	Acomys	Mus	6	8	0.137	ns	0.137	ns

Simple pairwise comparison (within groups)

	group	.y.	group1	group2	n1	n2	p.adj	p.adj.signif
1	Acomys	score	control	dpi.004	6	6	1	ns
2	Acomys	score	control	dpi.014	6	6	1	ns
3	Acomys	score	control	dpi.042	6	6	0.272	ns
4	Acomys	score	control	dpi.100	6	6	0.368	ns
5	Acomys	score	dpi.004	dpi.014	6	6	1	ns
6	Acomys	score	dpi.004	dpi.042	6	6	0.103	ns
7	Acomys	score	dpi.004	dpi.100	6	6	0.269	ns
8	Acomys	score	dpi.014	dpi.042	6	6	0.222	ns
9	Acomys	score	dpi.014	dpi.100	6	6	0.276	ns
10	Acomys	score	dpi.042	dpi.100	6	6	1	ns
11	Mus	score	control	dpi.004	8	8	0.095	ns
12	Mus	score	control	dpi.014	8	8	0.043	*
13	Mus	score	control	dpi.042	8	8	1	ns
14	Mus	score	control	dpi.100	8	8	1	ns
15	Mus	score	dpi.004	dpi.014	8	8	0.708	ns
16	Mus	score	dpi.004	dpi.042	8	8	1	ns
17	Mus	score	dpi.004	dpi.100	8	8	1	ns
18	Mus	score	dpi.014	dpi.042	8	8	0.316	ns
19	Mus	score	dpi.014	dpi.100	8	8	1	ns
20	Mus	score	dpi.042	dpi.100	8	8	1	ns

Figure 3a (Hydroxyproline assay)

Two-way mixed ANOVA result

	Effect	DFn	DFd	F	p	p<.05	ges
1	group	1	6	72.244	0.00015	*	0.496
2	time	4	24	15.289	2.42E-06	*	0.701
3	group:time	4	24	4.125	0.011	*	0.387

Simple pairwise comparison (between groups)

	time	.y.	group1	group2	n1	n2	p	p.signif	p.adj	p.adj.signif
1	control	score	Acomys	Mus	10	12	0.586	ns	0.586	ns
2	dpi.004	score	Acomys	Mus	10	12	0.73	ns	0.73	ns
3	dpi.014	score	Acomys	Mus	10	12	0.0103	*	0.0103	*
4	dpi.042	score	Acomys	Mus	10	12	0.0107	*	0.0107	*
5	dpi.100	score	Acomys	Mus	10	12	0.00318	**	0.00318	**

Simple pairwise comparison (within groups)

	group	.y.	group1	group2	n1	n2	p.adj	p.adj.signif
1	Acomys	score	control	dpi.004	10	10	1	ns
2	Acomys	score	control	dpi.014	10	10	0.073	ns
3	Acomys	score	control	dpi.042	10	10	0.006	**
4	Acomys	score	control	dpi.100	10	10	0.01	**
5	Acomys	score	dpi.004	dpi.014	10	10	0.161	ns
6	Acomys	score	dpi.004	dpi.042	10	10	0.012	*
7	Acomys	score	dpi.004	dpi.100	10	10	0.053	ns
8	Acomys	score	dpi.014	dpi.042	10	10	1	ns
9	Acomys	score	dpi.014	dpi.100	10	10	1	ns
10	Acomys	score	dpi.042	dpi.100	10	10	1	ns
11	Mus	score	control	dpi.004	12	12	1	ns
12	Mus	score	control	dpi.014	12	12	0.571	ns
13	Mus	score	control	dpi.042	12	12	0.027	*
14	Mus	score	control	dpi.100	12	12	0.348	ns
15	Mus	score	dpi.004	dpi.014	12	12	0.413	ns
16	Mus	score	dpi.004	dpi.042	12	12	0.036	*
17	Mus	score	dpi.004	dpi.100	12	12	1	ns
18	Mus	score	dpi.014	dpi.042	12	12	1	ns
19	Mus	score	dpi.014	dpi.100	12	12	1	ns
20	Mus	score	dpi.042	dpi.100	12	12	1	ns

Figure 3b (TNBSA assay)

Two-way mixed ANOVA result

	Effect	DFn	DFd	F	p	p<.05	ges
1	group	1	7	45.852	0.00026	*	0.386
2	time	1.3	9.13	7.665	0.017	*	0.497
3	group:time	1.3	9.13	1.089	0.345		0.123

Simple pairwise comparison (between groups)

	time	.y.	group1	group2	n1	n2	p	p.signif	p.adj	p.adj.signif
1	control	score	Acomys	Mus	10	11	1	ns	1	ns
2	dpi.004	score	Acomys	Mus	10	11	0.0111	*	0.0111	*
3	dpi.014	score	Acomys	Mus	10	11	0.00556	**	0.00556	**
4	dpi.100	score	Acomys	Mus	10	11	0.00823	**	0.00823	**

Simple pairwise comparison (within groups)

	group	.y.	group1	group2	n1	n2	p.adj	p.adj.signif
1	Acomys	score	control	dpi.004	10	10	1	ns
2	Acomys	score	control	dpi.014	10	10	0.071	ns
3	Acomys	score	control	dpi.100	10	10	0.036	*
4	Acomys	score	dpi.004	dpi.014	10	10	0.279	ns
5	Acomys	score	dpi.004	dpi.100	10	10	0.108	ns
6	Acomys	score	dpi.014	dpi.100	10	10	0.445	ns
7	Mus	score	control	dpi.004	11	11	1	ns
8	Mus	score	control	dpi.014	11	11	1	ns
9	Mus	score	control	dpi.100	11	11	0.221	ns
10	Mus	score	dpi.004	dpi.014	11	11	0.113	ns
11	Mus	score	dpi.004	dpi.100	11	11	0.42	ns
12	Mus	score	dpi.014	dpi.100	11	11	1	ns

Figure 3c (Birefringence 14 dpi)

Two-way mixed ANOVA result

	Effect	DFn	DFd	F	p	p<.05	ges
1	group	1	8	4.40E-18	1		9.63E-32
2	time	3	24	64.027	1.35E-11	*	0.889
3	group:time	3	24	0.106	0.956		0.013

Simple pairwise comparison (between groups)

	time	.y.	group1	group2	n1	n2	p	p.signif	p.adj	p.adj.signif
1	a.red	score	Acomys	Mus	6	4	7.45E-01	ns	0.745	ns
2	b.orange	score	Acomys	Mus	6	4	0.514	ns	0.514	ns
3	c.yellow	score	Acomys	Mus	6	4	0.955	ns	0.955	ns
4	d.green	score	Acomys	Mus	6	4	0.798	ns	0.798	ns

Simple pairwise comparison (within groups)

	group	.y.	group1	group2	n1	n2	p.adj	p.adj.signif
1	Acomys	score	a.red	b.orange	6	6	9.00E-03	**
2	Acomys	score	a.red	c.yellow	6	6	0.016	*
3	Acomys	score	a.red	d.green	6	6	0.00055	***
4	Acomys	score	b.orange	c.yellow	6	6	0.131	ns
5	Acomys	score	b.orange	d.green	6	6	0.0004	***
6	Acomys	score	c.yellow	d.green	6	6	0.491	ns
7	Mus	score	a.red	b.orange	4	4	1	ns
8	Mus	score	a.red	c.yellow	4	4	0.137	ns
9	Mus	score	a.red	d.green	4	4	0.031	*
10	Mus	score	b.orange	c.yellow	4	4	0.032	*
11	Mus	score	b.orange	d.green	4	4	0.018	*
12	Mus	score	c.yellow	d.green	4	4	0.205	ns

Figure 3c (Birefringence 100 dpi)

Two-way mixed ANOVA result

	Effect	DFn	DFd	F	p	p<.05	ges
1	group	1	6	5.04E-01	0.504		3.42E-15
2	time	1.05	6.3	507.543	2.72E-07	*	0.988
3	group:time	1.05	6.3	15.59	0.007	*	0.722

Simple pairwise comparison (between groups)

	time	.y.	group1	group2	n1	n2	p	p.signif	p.adj	p.adj.signif
1	a.red	score	Acomys	Mus	4	4	7.15E-03	**	0.00715	**
2	b.orange	score	Acomys	Mus	4	4	0.00365	**	0.00365	**
3	c.yellow	score	Acomys	Mus	4	4	0.0583	ns	0.0583	ns
4	d.green	score	Acomys	Mus	4	4	0.00282	**	0.00282	**

Simple pairwise comparison (within groups)

	group	.y.	group1	group2	n1	n2	p.adj	p.adj.signif
1	Acomys	score	a.red	b.orange	4	4	9.24E-04	***
2	Acomys	score	a.red	c.yellow	4	4	0.001	**
3	Acomys	score	a.red	d.green	4	4	0.00046	***
4	Acomys	score	b.orange	c.yellow	4	4	0.064	ns
5	Acomys	score	b.orange	d.green	4	4	0.006	**
6	Acomys	score	c.yellow	d.green	4	4	0.03	*
7	Mus	score	a.red	b.orange	4	4	0.013	*
8	Mus	score	a.red	c.yellow	4	4	0.011	*
9	Mus	score	a.red	d.green	4	4	0.006	**
10	Mus	score	b.orange	c.yellow	4	4	0.008	**
11	Mus	score	b.orange	d.green	4	4	0.022	*
12	Mus	score	c.yellow	d.green	4	4	0.175	ns

Figure 3e (Waviness index)

Two-way mixed ANOVA result

	Effect	DFn	DFd	F	p	p<.05	ges
1	group	1	6	1.09E+01	1.60E-02	*	4.39E-01
2	time	1	6	0.054	8.25E-01		0.005
3	group:time	1	6	1.905	0.217		1.53E-01

Simple pairwise comparison (between groups)

	time	.y.	group1	group2	n1	n2	p	p.signif	p.adj	p.adj.signif
1	dpi.014	score	Acomys	Mus	4	4	2.66E-03	**	0.00266	**
2	dpi.100	score	Acomys	Mus	4	4	0.404	ns	0.404	ns

Simple pairwise comparison (within groups)

	group	.y.	group1	group2	n1	n2	p.adj	p.adj.signif
1	Acomys	score	dpi.014	dpi.100	4	4	5.54E-01	ns
2	Mus	score	dpi.014	dpi.100	4	4	0.207	ns

Figure 4l (MLC2v pHis immuno)

Two-way mixed ANOVA result

	Effect	DFn	DFd	F	p	p<.05	ges
1	group	1	6	6.091	0.049	*	0.337
2	time	3	18	14.206	5.41E-05	*	0.542
3	group:time	3	18	3.01	0.057		0.2

Simple pairwise comparison (between groups)

	time	.y.	group1	group2	n1	n2	p	p.signif	p.adj	p.adj.signif
1	control	score	Acomys	Mus	5	4	0.87	ns	0.87	ns
2	dpi.014	score	Acomys	Mus	5	4	0.0543	ns	0.0543	ns
3	dpi.042	score	Acomys	Mus	5	4	0.0568	ns	0.0568	ns
4	dpi.100	score	Acomys	Mus	5	4	0.234	ns	0.234	ns

Simple pairwise comparison (within groups)

	group	.y.	group1	group2	n1	n2	p.adj	p.adj.signif
1	Acomys	score	control	dpi.014	5	5	0.223	ns
2	Acomys	score	control	dpi.042	5	5	0.09	ns
3	Acomys	score	control	dpi.100	5	5	0.4	ns
4	Acomys	score	dpi.014	dpi.042	5	5	1	ns
5	Acomys	score	dpi.014	dpi.100	5	5	0.397	ns
6	Acomys	score	dpi.042	dpi.100	5	5	0.037	*
7	Mus	score	control	dpi.014	4	4	0.065	ns
8	Mus	score	control	dpi.042	4	4	0.347	ns
9	Mus	score	control	dpi.100	4	4	0.84	ns
10	Mus	score	dpi.014	dpi.042	4	4	1	ns
11	Mus	score	dpi.014	dpi.100	4	4	1	ns
12	Mus	score	dpi.042	dpi.100	4	4	1	ns

Figure 5a (pHis ERG immuno)

Two-way mixed ANOVA result

	Effect	DFn	DFd	F	p	p<.05	ges
1	group	1	5	8.57	0.033	*	0.368
2	time	4	20	16.09	4.84E-06	*	0.68
3	group:time	4	20	4.974	0.006	*	0.397

Simple pairwise comparison (between groups)

	time	.y.	group1	group2	n1	n2	p	p.signif	p.adj	p.adj.signif
1	dpi.004	score	Acomys	Mus	5	4	0.264	ns	0.264	ns
2	dpi.014	score	Acomys	Mus	5	4	0.138	ns	0.138	ns
3	dpi.042	score	Acomys	Mus	5	4	0.0122	*	0.0122	*
4	dpi.100	score	Acomys	Mus	5	4	0.00486	**	0.00486	**

Simple pairwise comparison (within groups)

	group	.y.	group1	group2	n1	n2	p.adj	p.adj.signif
1	Acomys	score	control	dpi.004	5	5	0.159	ns
2	Acomys	score	control	dpi.014	5	5	0.011	*
3	Acomys	score	control	dpi.042	5	5	0.261	ns
4	Acomys	score	control	dpi.100	5	5	0.025	*
5	Acomys	score	dpi.004	dpi.014	5	5	0.083	ns
6	Acomys	score	dpi.004	dpi.042	5	5	1	ns
7	Acomys	score	dpi.004	dpi.100	5	5	1	ns
8	Acomys	score	dpi.014	dpi.042	5	5	0.502	ns
9	Acomys	score	dpi.014	dpi.100	5	5	0.156	ns
10	Acomys	score	dpi.042	dpi.100	5	5	0.974	ns
11	Mus	score	control	dpi.004	4	4	0.104	ns
12	Mus	score	control	dpi.014	4	4	0.549	ns
13	Mus	score	control	dpi.042	4	4	0.236	ns
14	Mus	score	control	dpi.100	4	4	0.803	ns
15	Mus	score	dpi.004	dpi.014	4	4	0.272	ns
16	Mus	score	dpi.004	dpi.042	4	4	0.28	ns
17	Mus	score	dpi.004	dpi.100	4	4	0.28	ns
18	Mus	score	dpi.014	dpi.042	4	4	1	ns
19	Mus	score	dpi.014	dpi.100	4	4	1	ns
20	Mus	score	dpi.042	dpi.100	4	4	1	ns

Figure 5c (aSMA immuno)

Two-way mixed ANOVA result

	Effect	DFn	DFd	F	p	p<.05	ges
1	group	1	4	9.571	0.036	*	0.289
2	time	3	12	8.563	0.003	*	0.64
3	group:time	3	12	4.449	0.025	*	0.48

Simple pairwise comparison (between groups)

	time	.y.	group1	group2	n1	n2	p	p.signif	p.adj	p.adj.signif
1	dpi.014	score	Acomys	Mus	4	3	0.142	ns	0.142	ns
2	dpi.042	score	Acomys	Mus	4	3	0.00232	**	0.00232	**
3	dpi.100	score	Acomys	Mus	4	3	0.0913	ns	0.0913	ns

Simple pairwise comparison (within groups)

	group	.y.	group1	group2	n1	n2	p.adj	p.adj.signif
1	Acomys	score	control	dpi.014	4	4	0.113	ns
2	Acomys	score	control	dpi.042	4	4	0.025	*
3	Acomys	score	control	dpi.100	4	4	0.59	ns
4	Acomys	score	dpi.014	dpi.042	4	4	0.02	*
5	Acomys	score	dpi.014	dpi.100	4	4	0.84	ns
6	Acomys	score	dpi.042	dpi.100	4	4	1	ns
7	Mus	score	control	dpi.014	3	3	0.442	ns
8	Mus	score	control	dpi.042	3	3	0.08	ns
9	Mus	score	control	dpi.100	3	3	0.224	ns
10	Mus	score	dpi.014	dpi.042	3	3	1	ns
11	Mus	score	dpi.014	dpi.100	3	3	1	ns
12	Mus	score	dpi.042	dpi.100	3	3	1	ns

Supplementary Figure 2b (Injured area)

Two-way mixed ANOVA result

	Effect	DFn	DFd	F	p	p<.05	ges
1	group	1	6	0.803	0.405		0.018
2	time	1.25	7.5	23.201	0.001	*	0.769
3	group:time	1.25	7.5	0.618	0.491		0.081

Simple pairwise comparison (between groups)

	time	.y.	group1	group2	n1	n2	p	p.signif	p.adj	p.adj.signif
1	control	score	Acomys	Mus	4	4	0.927	ns	0.927	ns
2	dpi.004	score	Acomys	Mus	4	4	0.672	ns	0.672	ns
3	dpi.014	score	Acomys	Mus	4	4	0.0347	*	0.0347	*
4	dpi.042	score	Acomys	Mus	4	4	0.178	ns	0.178	ns
5	dpi.100	score	Acomys	Mus	4	4	0.0819	ns	0.0819	ns

Simple pairwise comparison (within groups)

	group	.y.	group1	group2	n1	n2	p.adj	p.adj.signif
1	Acomys	score	control	dpi.004	4	4	0.325	ns
2	Acomys	score	control	dpi.014	4	4	0.241	ns
3	Acomys	score	control	dpi.042	4	4	0.41	ns
4	Acomys	score	control	dpi.100	4	4	0.538	ns
5	Acomys	score	dpi.004	dpi.014	4	4	0.617	ns
6	Acomys	score	dpi.004	dpi.042	4	4	0.54	ns
7	Acomys	score	dpi.004	dpi.100	4	4	0.533	ns
8	Acomys	score	dpi.014	dpi.042	4	4	1	ns
9	Acomys	score	dpi.014	dpi.100	4	4	1	ns
10	Acomys	score	dpi.042	dpi.100	4	4	1	ns
11	Mus	score	control	dpi.004	4	4	0.135	ns
12	Mus	score	control	dpi.014	4	4	0.05	ns
13	Mus	score	control	dpi.042	4	4	0.162	ns
14	Mus	score	control	dpi.100	4	4	0.04	*
15	Mus	score	dpi.004	dpi.014	4	4	0.489	ns
16	Mus	score	dpi.004	dpi.042	4	4	0.251	ns
17	Mus	score	dpi.004	dpi.100	4	4	0.439	ns
18	Mus	score	dpi.014	dpi.042	4	4	1	ns
19	Mus	score	dpi.014	dpi.100	4	4	1	ns
20	Mus	score	dpi.042	dpi.100	4	4	1	ns

Supplementary Figure 2e (Ejection fraction)

Two-way mixed ANOVA result

	Effect	DFn	DFd	F	p	p<.05	ges
1	group	1	6	4.825	0.07		0.104
2	time	4	24	3.314	0.027	*	0.321
3	group:time	4	24	1.254	0.315		0.152

Simple pairwise comparison (between groups)

	time	.y.	group1	group2	n1	n2	p	p.signif	p.adj	p.adj.signif
1	control	score	Acomys	Mus	15	14	0.541	ns	0.541	ns
2	dpi.004	score	Acomys	Mus	15	14	0.00772	**	0.00772	**
3	dpi.014	score	Acomys	Mus	15	14	1.31E-05	****	1.31E-05	****
4	dpi.042	score	Acomys	Mus	15	14	3.33E-10	****	3.33E-10	****
5	dpi.100	score	Acomys	Mus	15	14	8.24E-11	****	8.24E-11	****

Simple pairwise comparison (within groups)

	group	.y.	group1	group2	n1	n2	p.adj	p.adj.signif
1	Acomys	score	control	dpi.004	15	15	1	ns
2	Acomys	score	control	dpi.014	15	15	1	ns
3	Acomys	score	control	dpi.042	15	15	1	ns
4	Acomys	score	control	dpi.100	15	15	1	ns
5	Acomys	score	dpi.004	dpi.014	15	15	1	ns
6	Acomys	score	dpi.004	dpi.042	15	15	1	ns
7	Acomys	score	dpi.004	dpi.100	15	15	1	ns
8	Acomys	score	dpi.014	dpi.042	15	15	1	ns
9	Acomys	score	dpi.014	dpi.100	15	15	1	ns
10	Acomys	score	dpi.042	dpi.100	15	15	1	ns
11	Mus	score	control	dpi.004	14	14	0.285	ns
12	Mus	score	control	dpi.014	14	14	0.094	ns
13	Mus	score	control	dpi.042	14	14	0.26	ns
14	Mus	score	control	dpi.100	14	14	0.42	ns
15	Mus	score	dpi.004	dpi.014	14	14	1	ns
16	Mus	score	dpi.004	dpi.042	14	14	0.336	ns
17	Mus	score	dpi.004	dpi.100	14	14	1	ns
18	Mus	score	dpi.014	dpi.042	14	14	1	ns
19	Mus	score	dpi.014	dpi.100	14	14	0.193	ns
20	Mus	score	dpi.042	dpi.100	14	14	0.738	ns

Supplementary Figure 2e (Fractional shortening)

Two-way mixed ANOVA result

	Effect	DFn	DFd	F	p	p<.05	ges
1	group	1	6	2.465	0.167		0.116
2	time	4	24	7.85	0.00034	*	0.471
3	group:time	4	24	1.931	0.138		0.18

Simple pairwise comparison (between groups)

	time	.y.	group1	group2	n1	n2	p	p.signif	p.adj	p.adj.signif
1	control	score	Acomys	Mus	15	14	0.496	ns	0.496	ns
2	dpi.004	score	Acomys	Mus	15	14	0.396	ns	0.396	ns
3	dpi.014	score	Acomys	Mus	15	14	0.0316	*	0.0316	*
4	dpi.042	score	Acomys	Mus	15	14	0.0613	ns	0.0613	ns
5	dpi.100	score	Acomys	Mus	15	14	0.336	ns	0.336	ns

Simple pairwise comparison (within groups)

	group	.y.	group1	group2	n1	n2	p.adj	p.adj.signif
1	Acomys	score	control	dpi.004	15	15	1	ns
2	Acomys	score	control	dpi.014	15	15	1	ns
3	Acomys	score	control	dpi.042	15	15	1	ns
4	Acomys	score	control	dpi.100	15	15	1	ns
5	Acomys	score	dpi.004	dpi.014	15	15	1	ns
6	Acomys	score	dpi.004	dpi.042	15	15	1	ns
7	Acomys	score	dpi.004	dpi.100	15	15	1	ns
8	Acomys	score	dpi.014	dpi.042	15	15	1	ns
9	Acomys	score	dpi.014	dpi.100	15	15	1	ns
10	Acomys	score	dpi.042	dpi.100	15	15	1	ns
11	Mus	score	control	dpi.004	14	14	0.015	*
12	Mus	score	control	dpi.014	14	14	0.054	ns
13	Mus	score	control	dpi.042	14	14	0.079	ns
14	Mus	score	control	dpi.100	14	14	0.092	ns
15	Mus	score	dpi.004	dpi.014	14	14	1	ns
16	Mus	score	dpi.004	dpi.042	14	14	1	ns
17	Mus	score	dpi.004	dpi.100	14	14	1	ns
18	Mus	score	dpi.014	dpi.042	14	14	1	ns
19	Mus	score	dpi.014	dpi.100	14	14	0.154	ns
20	Mus	score	dpi.042	dpi.100	14	14	0.483	ns

Supplementary Figure 2g (Q amplitude)

Two-way mixed ANOVA result

	Effect	DFn	DFd	F	p	p<.05	ges
1	group	1	6	8.397	0.027	*	0.301
2	time	1.35	8.13	2.467	0.152		0.221
3	group:time	1.35	8.13	2.947	0.119		0.254

Simple pairwise comparison (between groups)

	time	.y.	group1	group2	n1	n2	p	p.signif	p.adj	p.adj.signif
1	control	score	Acomys	Mus	15	14	7.84E-06	****	7.84E-06	****
2	dpi.004	score	Acomys	Mus	15	14	0.738	ns	0.738	ns
3	dpi.014	score	Acomys	Mus	15	14	0.00343	**	0.00343	**
4	dpi.042	score	Acomys	Mus	15	14	0.00025	***	0.00025	***
5	dpi.100	score	Acomys	Mus	15	14	0.00013	***	0.00013	***

Simple pairwise comparison (within groups)

	group	.y.	group1	group2	n1	n2	p.adj	p.adj.signif
1	Acomys	score	control	dpi.004	15	15	0.584	ns
2	Acomys	score	control	dpi.014	15	15	0.466	ns
3	Acomys	score	control	dpi.042	15	15	0.291	ns
4	Acomys	score	control	dpi.100	15	15	0.198	ns
5	Acomys	score	dpi.004	dpi.014	15	15	1	ns
6	Acomys	score	dpi.004	dpi.042	15	15	1	ns
7	Acomys	score	dpi.004	dpi.100	15	15	1	ns
8	Acomys	score	dpi.014	dpi.042	15	15	1	ns
9	Acomys	score	dpi.014	dpi.100	15	15	1	ns
10	Acomys	score	dpi.042	dpi.100	15	15	1	ns
11	Mus	score	control	dpi.004	14	14	1	ns
12	Mus	score	control	dpi.014	14	14	1	ns
13	Mus	score	control	dpi.042	14	14	1	ns
14	Mus	score	control	dpi.100	14	14	1	ns
15	Mus	score	dpi.004	dpi.014	14	14	1	ns
16	Mus	score	dpi.004	dpi.042	14	14	1	ns
17	Mus	score	dpi.004	dpi.100	14	14	1	ns
18	Mus	score	dpi.014	dpi.042	14	14	1	ns
19	Mus	score	dpi.014	dpi.100	14	14	1	ns
20	Mus	score	dpi.042	dpi.100	14	14	1	ns

Supplementary Figure 2g (Q duration)

Two-way mixed ANOVA result

	Effect	DFn	DFd	F	p	p<.05	ges
1	group	1	6	8.252	0.028	*	0.099
2	time	4	24	0.489	0.744		0.07
3	group:time	4	24	0.649	0.633		0.091

Simple pairwise comparison (between groups)

	time	.y.	group1	group2	n1	n2	p	p.signif	p.adj	p.adj.signif
1	control	score	Acomys	Mus	15	14	0.00018	***	0.00018	***
2	dpi.004	score	Acomys	Mus	15	14	0.742	ns	0.742	ns
3	dpi.014	score	Acomys	Mus	15	14	0.0312	*	0.0312	*
4	dpi.042	score	Acomys	Mus	15	14	0.213	ns	0.213	ns
5	dpi.100	score	Acomys	Mus	15	14	0.23	ns	0.23	ns

Simple pairwise comparison (within groups)

	group	.y.	group1	group2	n1	n2	p.adj	p.adj.signif
1	Acomys	score	control	dpi.004	15	15	1	ns
2	Acomys	score	control	dpi.014	15	15	1	ns
3	Acomys	score	control	dpi.042	15	15	1	ns
4	Acomys	score	control	dpi.100	15	15	1	ns
5	Acomys	score	dpi.004	dpi.014	15	15	1	ns
6	Acomys	score	dpi.004	dpi.042	15	15	1	ns
7	Acomys	score	dpi.004	dpi.100	15	15	1	ns
8	Acomys	score	dpi.014	dpi.042	15	15	1	ns
9	Acomys	score	dpi.014	dpi.100	15	15	1	ns
10	Acomys	score	dpi.042	dpi.100	15	15	1	ns
11	Mus	score	control	dpi.004	14	14	1	ns
12	Mus	score	control	dpi.014	14	14	1	ns
13	Mus	score	control	dpi.042	14	14	1	ns
14	Mus	score	control	dpi.100	14	14	1	ns
15	Mus	score	dpi.004	dpi.014	14	14	1	ns
16	Mus	score	dpi.004	dpi.042	14	14	1	ns
17	Mus	score	dpi.004	dpi.100	14	14	1	ns
18	Mus	score	dpi.014	dpi.042	14	14	1	ns
19	Mus	score	dpi.014	dpi.100	14	14	1	ns
20	Mus	score	dpi.042	dpi.100	14	14	1	ns

Supplementary Figure 2g (R amplitude)

Two-way mixed ANOVA result

	Effect	DFn	DFd	F	p	p<.05	ges
1	group	1	6	1.341	0.291		0.049
2	time	4	24	3.241	0.029	*	0.294
3	group:time	4	24	1.464	0.244		0.158

Simple pairwise comparison (between groups)

	time	.y.	group1	group2	n1	n2	p	p.signif	p.adj	p.adj.signif
1	control	score	Acomys	Mus	15	14	0.125	ns	0.125	ns
2	dpi.004	score	Acomys	Mus	15	14	0.0647	ns	0.0647	ns
3	dpi.014	score	Acomys	Mus	15	14	0.975	ns	0.975	ns
4	dpi.042	score	Acomys	Mus	15	14	0.649	ns	0.649	ns
5	dpi.100	score	Acomys	Mus	15	14	0.814	ns	0.814	ns

Simple pairwise comparison (within groups)

	group	.y.	group1	group2	n1	n2	p.adj	p.adj.signif
1	Acomys	score	control	dpi.004	15	15	1	ns
2	Acomys	score	control	dpi.014	15	15	1	ns
3	Acomys	score	control	dpi.042	15	15	1	ns
4	Acomys	score	control	dpi.100	15	15	1	ns
5	Acomys	score	dpi.004	dpi.014	15	15	1	ns
6	Acomys	score	dpi.004	dpi.042	15	15	1	ns
7	Acomys	score	dpi.004	dpi.100	15	15	1	ns
8	Acomys	score	dpi.014	dpi.042	15	15	0.413	ns
9	Acomys	score	dpi.014	dpi.100	15	15	1	ns
10	Acomys	score	dpi.042	dpi.100	15	15	1	ns
11	Mus	score	control	dpi.004	14	14	0.416	ns
12	Mus	score	control	dpi.014	14	14	1	ns
13	Mus	score	control	dpi.042	14	14	1	ns
14	Mus	score	control	dpi.100	14	14	1	ns
15	Mus	score	dpi.004	dpi.014	14	14	0.053	ns
16	Mus	score	dpi.004	dpi.042	14	14	1	ns
17	Mus	score	dpi.004	dpi.100	14	14	0.475	ns
18	Mus	score	dpi.014	dpi.042	14	14	1	ns
19	Mus	score	dpi.014	dpi.100	14	14	1	ns
20	Mus	score	dpi.042	dpi.100	14	14	1	ns

Supplementary Figure 2g (R duration)

Two-way mixed ANOVA result

	Effect	DFn	DFd	F	p	p<.05	ges
1	group	1	6	0.118	0.743		0.005
2	time	4	24	1.41	0.261		0.146
3	group:time	4	24	2.121	0.109		0.205

Simple pairwise comparison (between groups)

	time	.y.	group1	group2	n1	n2	p	p.signif	p.adj	p.adj.signif
1	control	score	Acomys	Mus	15	14	0.125	ns	0.125	ns
2	dpi.004	score	Acomys	Mus	15	14	0.0647	ns	0.0647	ns
3	dpi.014	score	Acomys	Mus	15	14	0.975	ns	0.975	ns
4	dpi.042	score	Acomys	Mus	15	14	0.649	ns	0.649	ns
5	dpi.100	score	Acomys	Mus	15	14	0.814	ns	0.814	ns

Simple pairwise comparison (within groups)

	group	.y.	group1	group2	n1	n2	p.adj	p.adj.signif
1	Acomys	score	control	dpi.004	15	15	1	ns
2	Acomys	score	control	dpi.014	15	15	1	ns
3	Acomys	score	control	dpi.042	15	15	1	ns
4	Acomys	score	control	dpi.100	15	15	1	ns
5	Acomys	score	dpi.004	dpi.014	15	15	1	ns
6	Acomys	score	dpi.004	dpi.042	15	15	1	ns
7	Acomys	score	dpi.004	dpi.100	15	15	1	ns
8	Acomys	score	dpi.014	dpi.042	15	15	1	ns
9	Acomys	score	dpi.014	dpi.100	15	15	1	ns
10	Acomys	score	dpi.042	dpi.100	15	15	1	ns
11	Mus	score	control	dpi.004	14	14	1	ns
12	Mus	score	control	dpi.014	14	14	1	ns
13	Mus	score	control	dpi.042	14	14	1	ns
14	Mus	score	control	dpi.100	14	14	1	ns
15	Mus	score	dpi.004	dpi.014	14	14	1	ns
16	Mus	score	dpi.004	dpi.042	14	14	1	ns
17	Mus	score	dpi.004	dpi.100	14	14	0.726	ns
18	Mus	score	dpi.014	dpi.042	14	14	1	ns
19	Mus	score	dpi.014	dpi.100	14	14	1	ns
20	Mus	score	dpi.042	dpi.100	14	14	0.794	ns

Supplementary Figure 2g (S amplitude)

Two-way mixed ANOVA result

	Effect	DFn	DFd	F	p	p<.05	ges
1	group	1	6	74.858	0.00013	*	0.818
2	time	2.31	13.85	1.318	0.303		0.123
3	group:time	2.31	13.85	1.032	0.392		0.099

Simple pairwise comparison (between groups)

	time	.y.	group1	group2	n1	n2	p	p.signif	p.adj	p.adj.signif
1	control	score	Acomys	Mus	15	14	1.27E-09	****	1.27E-09	****
2	dpi.004	score	Acomys	Mus	15	14	0.00042	***	0.00042	***
3	dpi.014	score	Acomys	Mus	15	14	0.00189	**	0.00189	**
4	dpi.042	score	Acomys	Mus	15	14	0.00047	***	0.00047	***
5	dpi.100	score	Acomys	Mus	15	14	0.00673	**	0.00673	**

Simple pairwise comparison (within groups)

	group	.y.	group1	group2	n1	n2	p.adj	p.adj.signif
1	Acomys	score	control	dpi.004	15	15	1	ns
2	Acomys	score	control	dpi.014	15	15	1	ns
3	Acomys	score	control	dpi.042	15	15	1	ns
4	Acomys	score	control	dpi.100	15	15	1	ns
5	Acomys	score	dpi.004	dpi.014	15	15	1	ns
6	Acomys	score	dpi.004	dpi.042	15	15	1	ns
7	Acomys	score	dpi.004	dpi.100	15	15	1	ns
8	Acomys	score	dpi.014	dpi.042	15	15	1	ns
9	Acomys	score	dpi.014	dpi.100	15	15	0.225	ns
10	Acomys	score	dpi.042	dpi.100	15	15	0.032	*
11	Mus	score	control	dpi.004	14	14	1	ns
12	Mus	score	control	dpi.014	14	14	0.336	ns
13	Mus	score	control	dpi.042	14	14	1	ns
14	Mus	score	control	dpi.100	14	14	1	ns
15	Mus	score	dpi.004	dpi.014	14	14	1	ns
16	Mus	score	dpi.004	dpi.042	14	14	1	ns
17	Mus	score	dpi.004	dpi.100	14	14	1	ns
18	Mus	score	dpi.014	dpi.042	14	14	1	ns
19	Mus	score	dpi.014	dpi.100	14	14	1	ns
20	Mus	score	dpi.042	dpi.100	14	14	1	ns

Supplementary Figure 2g (S duration)

Two-way mixed ANOVA result

	Effect	DFn	DFd	F	p	p<.05	ges
1	group	1	6	1.385	0.284		0.009
2	time	4	24	0.295	0.878		0.045
3	group:time	4	24	1.29	0.302		0.171

Simple pairwise comparison (between groups)

	time	.y.	group1	group2	n1	n2	p	p.signif	p.adj	p.adj.signif
1	control	score	Acomys	Mus	15	14	0.624	ns	0.624	ns
2	dpi.004	score	Acomys	Mus	15	14	0.127	ns	0.127	ns
3	dpi.014	score	Acomys	Mus	15	14	0.289	ns	0.289	ns
4	dpi.042	score	Acomys	Mus	15	14	0.655	ns	0.655	ns
5	dpi.100	score	Acomys	Mus	15	14	0.915	ns	0.915	ns

Simple pairwise comparison (within groups)

	group	.y.	group1	group2	n1	n2	p.adj	p.adj.signif
1	Acomys	score	control	dpi.004	15	15	1	ns
2	Acomys	score	control	dpi.014	15	15	1	ns
3	Acomys	score	control	dpi.042	15	15	0.69	ns
4	Acomys	score	control	dpi.100	15	15	1	ns
5	Acomys	score	dpi.004	dpi.014	15	15	1	ns
6	Acomys	score	dpi.004	dpi.042	15	15	1	ns
7	Acomys	score	dpi.004	dpi.100	15	15	1	ns
8	Acomys	score	dpi.014	dpi.042	15	15	1	ns
9	Acomys	score	dpi.014	dpi.100	15	15	1	ns
10	Acomys	score	dpi.042	dpi.100	15	15	1	ns
11	Mus	score	control	dpi.004	14	14	0.257	ns
12	Mus	score	control	dpi.014	14	14	1	ns
13	Mus	score	control	dpi.042	14	14	1	ns
14	Mus	score	control	dpi.100	14	14	1	ns
15	Mus	score	dpi.004	dpi.014	14	14	1	ns
16	Mus	score	dpi.004	dpi.042	14	14	1	ns
17	Mus	score	dpi.004	dpi.100	14	14	1	ns
18	Mus	score	dpi.014	dpi.042	14	14	1	ns
19	Mus	score	dpi.014	dpi.100	14	14	1	ns
20	Mus	score	dpi.042	dpi.100	14	14	1	ns

Supplementary Figure 3a (PDGFRB immuno)

Two-way mixed ANOVA result

	Effect	DFn	DFd	F	p	p<.05	ges
1	group	1	5	15.13	0.012	*	0.238
2	time	1.61	8.04	38.241	0.00011	*	0.873
3	group:time	1.61	8.04	1.242	0.327		0.182

Simple pairwise comparison (between groups)

	time	.y.	group1	group2	n1	n2	p	p.signif	p.adj	p.adj.signif
1	dpi.004	score	Acomys	Mus	5	4	0.291	ns	0.291	ns
2	dpi.014	score	Acomys	Mus	5	4	0.17	ns	0.17	ns
3	dpi.100	score	Acomys	Mus	5	4	0.0386	*	0.0386	*

Simple pairwise comparison (within groups)

	group	.y.	group1	group2	n1	n2	p.adj	p.adj.signif
1	Acomys	score	control	dpi.004	5	5	0.154	ns
2	Acomys	score	control	dpi.014	5	5	0.034	*
3	Acomys	score	control	dpi.100	5	5	0.011	*
4	Acomys	score	dpi.004	dpi.014	5	5	0.4	ns
5	Acomys	score	dpi.004	dpi.100	5	5	0.484	ns
6	Acomys	score	dpi.014	dpi.100	5	5	1	ns
7	Mus	score	control	dpi.004	4	4	0.00091	***
8	Mus	score	control	dpi.014	4	4	0.011	*
9	Mus	score	control	dpi.100	4	4	0.009	**
10	Mus	score	dpi.004	dpi.014	4	4	0.086	ns
11	Mus	score	dpi.004	dpi.100	4	4	0.011	*
12	Mus	score	dpi.014	dpi.100	4	4	1	ns

Supplementary Figure 4b (Nuclei numbers)

Two-way mixed ANOVA result

	Effect	DFn	DFd	F	p	p<.05	ges
1	group	1	5	13.757	0.014	*	0.143
2	time	4	20	138.102	2.89E-14	*	0.963
3	group:time	4	20	0.435	0.782		0.076

Simple pairwise comparison (between groups)

	time	.y.	group1	group2	n1	n2	p	p.signif	p.adj	p.adj.signif
1	control	score	Acomys	Mus	10	10	3.20E-10	****	3.20E-10	****
2	dpi.014	score	Acomys	Mus	10	10	8.23E-10	****	8.23E-10	****
3	dpi.042	score	Acomys	Mus	10	10	3.99E-07	****	3.99E-07	****
4	dpi.100	score	Acomys	Mus	10	10	5.27E-07	****	5.27E-07	****

Simple pairwise comparison (within groups)

	group	.y.	group1	group2	n1	n2	p.adj	p.adj.signif
1	Acomys	score	control	dpi.014	10	10	0.035	*
2	Acomys	score	control	dpi.042	10	10	0.001	**
3	Acomys	score	control	dpi.100	10	10	0.001	**
4	Acomys	score	dpi.014	dpi.042	10	10	0.024	*
5	Acomys	score	dpi.014	dpi.100	10	10	0.395	ns
6	Acomys	score	dpi.042	dpi.100	10	10	1	ns
7	Mus	score	control	dpi.014	10	10	0.00059	***
8	Mus	score	control	dpi.042	10	10	0.00011	***
9	Mus	score	control	dpi.100	10	10	0.00067	***
10	Mus	score	dpi.014	dpi.042	10	10	0.073	ns
11	Mus	score	dpi.014	dpi.100	10	10	0.29	ns
12	Mus	score	dpi.042	dpi.100	10	10	1	ns

Supplementary Figure 4c (Global proliferation)

Two-way mixed ANOVA result

	Effect	DFn	DFd	F	p	p<.05	ges
1	group	1	6	2.348	0.176		0.082
2	time	1.16	6.98	61.21	8.42E-05	*	0.887
3	group:time	1.16	6.98	1.146	0.332		0.128

Simple pairwise comparison (between groups)

	time	.y.	group1	group2	n1	n2	p	p.signif	p.adj	p.adj.signif
1	dpi.004	score	Acomys	Mus	5	4	0.0975	ns	0.0975	ns
2	dpi.014	score	Acomys	Mus	5	4	0.328	ns	0.328	ns
3	dpi.042	score	Acomys	Mus	5	4	0.768	ns	0.768	ns
4	dpi.100	score	Acomys	Mus	5	4	0.373	ns	0.373	ns

Simple pairwise comparison (within groups)

	group	.y.	group1	group2	n1	n2	p.adj	p.adj.signif
1	Acomys	score	control	dpi.004	5	5	0.004	**
2	Acomys	score	control	dpi.014	5	5	0.692	ns
3	Acomys	score	control	dpi.042	5	5	0.021	*
4	Acomys	score	control	dpi.100	5	5	0.332	ns
5	Acomys	score	dpi.004	dpi.014	5	5	0.265	ns
6	Acomys	score	dpi.004	dpi.042	5	5	0.007	**
7	Acomys	score	dpi.004	dpi.100	5	5	0.018	*
8	Acomys	score	dpi.014	dpi.042	5	5	1	ns
9	Acomys	score	dpi.014	dpi.100	5	5	1	ns
10	Acomys	score	dpi.042	dpi.100	5	5	1	ns
11	Mus	score	control	dpi.004	4	4	0.00056	***
12	Mus	score	control	dpi.014	4	4	0.149	ns
13	Mus	score	control	dpi.042	4	4	0.185	ns
14	Mus	score	control	dpi.100	4	4	0.201	ns
15	Mus	score	dpi.004	dpi.014	4	4	0.044	*
16	Mus	score	dpi.004	dpi.042	4	4	0.004	**
17	Mus	score	dpi.004	dpi.100	4	4	0.003	**
18	Mus	score	dpi.014	dpi.042	4	4	0.311	ns
19	Mus	score	dpi.014	dpi.100	4	4	0.269	ns
20	Mus	score	dpi.042	dpi.100	4	4	0.898	ns

Supplementary Figure 5d (MLC2v pHis immuno)

Two-way mixed ANOVA result

	Effect	DFn	DFd	F	p	p<.05	ges
1	group	1	6	19.7	0.004	*	0.535
2	time	1.06	6.35	26.2	0.002	*	0.739
3	group:time	1.06	6.35	9.727	0.018	*	0.513

Simple pairwise comparison (between groups)

	time	.y.	group1	group2	n1	n2	p	p.signif	p.adj	p.adj.signif
1	control	score	Acomys	Mus	5	4	0.946	ns	0.946	ns
2	dpi.014	score	Acomys	Mus	5	4	0.00465	**	0.00465	**
3	dpi.100	score	Acomys	Mus	5	4	0.0322	*	0.0322	*

Simple pairwise comparison (within groups)

	group	.y.	group1	group2	n1	n2	p.adj	p.adj.signif
1	Acomys	score	control	dpi.014	5	5	0.049	*
2	Acomys	score	control	dpi.100	5	5	0.057	ns
3	Acomys	score	dpi.014	dpi.100	5	5	0.127	ns
4	Mus	score	control	dpi.014	4	4	0.001	**
5	Mus	score	control	dpi.100	4	4	0.528	ns
6	Mus	score	dpi.014	dpi.100	4	4	0.016	*

Supplementary Figure 6a (IsoB4 EdU immuno)

Two-way mixed ANOVA result

	Effect	DFn	DFd	F	p	p<.05	ges
1	group	1	6	14.384	0.009	*	0.222
2	time	4	24	30.347	4.51E-09	*	0.817
3	group:time	4	24	3.529	0.021	*	0.341

Simple pairwise comparison (between groups)

	time	.y.	group1	group2	n1	n2	p	p.signif	p.adj	p.adj.signif
1	dpi.004	score	Acomys	Mus	5	4	0.572	ns	0.572	ns
2	dpi.014	score	Acomys	Mus	5	4	0.0297	*	0.0297	*
3	dpi.042	score	Acomys	Mus	5	4	0.0574	ns	0.0574	ns
4	dpi.100	score	Acomys	Mus	5	4	0.0316	*	0.0316	*

Simple pairwise comparison (within groups)

	group	.y.	group1	group2	n1	n2	p.adj	p.adj.signif
1	Acomys	score	control	dpi.004	5	5	0.016	*
2	Acomys	score	control	dpi.014	5	5	0.064	ns
3	Acomys	score	control	dpi.042	5	5	0.071	ns
4	Acomys	score	control	dpi.100	5	5	0.317	ns
5	Acomys	score	dpi.004	dpi.014	5	5	1	ns
6	Acomys	score	dpi.004	dpi.042	5	5	0.034	*
7	Acomys	score	dpi.004	dpi.100	5	5	0.928	ns
8	Acomys	score	dpi.014	dpi.042	5	5	0.304	ns
9	Acomys	score	dpi.014	dpi.100	5	5	0.693	ns
10	Acomys	score	dpi.042	dpi.100	5	5	1	ns
11	Mus	score	control	dpi.004	4	4	0.06	ns
12	Mus	score	control	dpi.014	4	4	0.917	ns
13	Mus	score	control	dpi.042	4	4	0.48	ns
14	Mus	score	control	dpi.100	4	4	1	ns
15	Mus	score	dpi.004	dpi.014	4	4	0.056	ns
16	Mus	score	dpi.004	dpi.042	4	4	0.238	ns
17	Mus	score	dpi.004	dpi.100	4	4	0.187	ns
18	Mus	score	dpi.014	dpi.042	4	4	1	ns
19	Mus	score	dpi.014	dpi.100	4	4	1	ns
20	Mus	score	dpi.042	dpi.100	4	4	0.565	ns

Supplementary Figure 6b (IsoB4 pHis immuno)

Two-way mixed ANOVA result

	Effect	DFn	DFd	F	p	p<.05	ges
1	group	1	5	4.093	0.099		0.231
2	time	4	20	15.737	5.70E-06	*	0.666
3	group:time	4	20	2.12	0.116		0.211

Simple pairwise comparison (between groups)

	time	.y.	group1	group2	n1	n2	p	p.signif	p.adj	p.adj.signif
1	dpi.004	score	Acomys	Mus	5	4	0.085	ns	0.085	ns
2	dpi.014	score	Acomys	Mus	5	4	0.97	ns	0.97	ns
3	dpi.042	score	Acomys	Mus	5	4	0.0386	*	0.0386	*
4	dpi.100	score	Acomys	Mus	5	4	0.162	ns	0.162	ns

Simple pairwise comparison (within groups)

	group	.y.	group1	group2	n1	n2	p.adj	p.adj.signif
1	Acomys	score	control	dpi.004	5	5	0.283	ns
2	Acomys	score	control	dpi.014	5	5	0.00018	***
3	Acomys	score	control	dpi.042	5	5	0.496	ns
4	Acomys	score	control	dpi.100	5	5	0.304	ns
5	Acomys	score	dpi.004	dpi.014	5	5	0.183	ns
6	Acomys	score	dpi.004	dpi.042	5	5	0.301	ns
7	Acomys	score	dpi.004	dpi.100	5	5	0.563	ns
8	Acomys	score	dpi.014	dpi.042	5	5	1	ns
9	Acomys	score	dpi.014	dpi.100	5	5	1	ns
10	Acomys	score	dpi.042	dpi.100	5	5	1	ns
11	Mus	score	control	dpi.004	4	4	0.148	ns
12	Mus	score	control	dpi.014	4	4	0.295	ns
13	Mus	score	control	dpi.042	4	4	0.041	*
14	Mus	score	control	dpi.100	4	4	1	ns
15	Mus	score	dpi.004	dpi.014	4	4	0.12	ns
16	Mus	score	dpi.004	dpi.042	4	4	0.196	ns
17	Mus	score	dpi.004	dpi.100	4	4	0.306	ns
18	Mus	score	dpi.014	dpi.042	4	4	0.676	ns
19	Mus	score	dpi.014	dpi.100	4	4	1	ns
20	Mus	score	dpi.042	dpi.100	4	4	1	ns
