# Supplementary material for: Ischemic tolerance and cardiac repair in the spiny mouse (Acomys)
Source: NPJ Regen Med. 2021 Nov 17;6:78. doi: 10.1038/s41536-021-00188-2 (PMC8599451; doi:10.1038/s41536-021-00188-2)
Supplement: Supplementary file 2 — Reporting Summary [file 41536_2021_188_MOESM2_ESM.pdf]

nature research

Corresponding author(s): Kerstin Bartscherer
Last updated by author(s): June 4th, 2021

Reporting Summary

Nature Research wishes to improve the reproducibility of the work that we publish. This form provides structure for consistency and transparency in reporting. For further information on Nature Research policies, see our Editorial Policies and the Editorial Policy Checklist.

Statistics

For all statistical analyses, confirm that the following items are present in the figure legend, table legend, main text, or Methods section.

n/a	Confirmed	
☐	☒	The exact sample size (*n*) for each experimental group/condition, given as a discrete number and unit of measurement
☐	☒	A statement on whether measurements were taken from distinct samples or whether the same sample was measured repeatedly
☐	☒	The statistical test(s) used AND whether they are one- or two-sided<br>*Only common tests should be described solely by name; describe more complex techniques in the Methods section.*
☐	☒	A description of all covariates tested
☐	☒	A description of any assumptions or corrections, such as tests of normality and adjustment for multiple comparisons
☐	☒	A full description of the statistical parameters including central tendency (e.g. means) or other basic estimates (e.g. regression coefficient) AND variation (e.g. standard deviation) or associated estimates of uncertainty (e.g. confidence intervals)
☐	☒	For null hypothesis testing, the test statistic (e.g. *F*, *t*, *r*) with confidence intervals, effect sizes, degrees of freedom and *P* value noted<br>*Give P values as exact values whenever suitable.*
☒	☐	For Bayesian analysis, information on the choice of priors and Markov chain Monte Carlo settings
☒	☐	For hierarchical and complex designs, identification of the appropriate level for tests and full reporting of outcomes
☐	☒	Estimates of effect sizes (e.g. Cohen's *d*, Pearson's *r*), indicating how they were calculated

Our web collection on statistics for biologists contains articles on many of the points above.

Software and code

Policy information about availability of computer code

Data collection	All raw histological and immuno images were collected as Leica Image File (LIF) format.<br><br>Illumina sequencing libraries were prepared using the TruSeq Small RNA Library Prep Kit (Illumina, #RS-200), followed by PCR amplification. Afterwards, libraries were sequenced paired-end using the Illumina NextSeq500.
Data analysis	Image analysis was performed using ImageJ (version 1.53c). Raw images were converted to TIFFs using Imaris software (version 9.3) for some ImageJ analyses or when used as representative image.<br><br>RNAseq samples were normalized and further processed in R (version 3.8) using the DESeq2 Bioconductor package (version 3.12). Differential analysis data from the DESeq2 pipeline was used for enrichment analysis, using the Bioconductor R package clusterProfiler. Gene symbols were mapped to EntrezIDs using the org.Mm.eg.db R package. The compareCluster function was used to calculate and compare gene clusters between samples.

For manuscripts utilizing custom algorithms or software that are central to the research but not yet described in published literature, software must be made available to editors and reviewers. We strongly encourage code deposition in a community repository (e.g. GitHub). See the Nature Research guidelines for submitting code & software for further information.

Data

Policy information about availability of data

All manuscripts must include a data availability statement. This statement should provide the following information, where applicable:

- Accession codes, unique identifiers, or web links for publicly available datasets
- A list of figures that have associated raw data
- A description of any restrictions on data availability

The sequencing datasets generated in this study are available under BioProject accession number PRJNA692361.

Field-specific reporting

Please select the one below that is the best fit for your research. If you are not sure, read the appropriate sections before making your selection.

☒ Life sciences ☐ Behavioural & social sciences ☐ Ecological, evolutionary & environmental sciences

For a reference copy of the document with all sections, see nature.com/documents/nr-reporting-summary-flat.pdf

Life sciences study design

All studies must disclose on these points even when the disclosure is negative.

Sample size	Sample sizes were determined based on prior experience and published reports on myocardial infarction in Mus musculus.
Data exclusions	Outliers were excluded and defined as three standard deviations above the average.
Replication	Replication was performed at least three times. Experiments were carried out in biological replicates as indicated.
Randomization	Animals were blindly assigned to the experimental groups.
Blinding	Only the ID number corresponding to the animal's unique identity was visible during analysis. ID numbers were linked to the respective experimental treatment after completion of the analysis.

Reporting for specific materials, systems and methods

We require information from authors about some types of materials, experimental systems and methods used in many studies. Here, indicate whether each material, system or method listed is relevant to your study. If you are not sure if a list item applies to your research, read the appropriate section before selecting a response.

Materials & experimental systems

n/a	Involved in the study	
☐	☒	Antibodies
☒	☐	Eukaryotic cell lines
☒	☐	Palaeontology and archaeology
☐	☒	Animals and other organisms
☒	☐	Human research participants
☒	☐	Clinical data
☒	☐	Dual use research of concern

Methods

n/a	Involved in the study	
☒	☐	ChIP-seq
☒	☐	Flow cytometry
☒	☐	MRI-based neuroimaging

Antibodies

Antibodies used	α-SMA-647 Novus Biologicals NBP2-34522AF647 (working dilution 1:100)<br>Phospho-Histone H3 Novus Biologicals NB600-1168 (working dilution 1:200)<br>α-SMA Abcam ab5694 (working dilution 1:200)<br>PDGFRβ Abcam ab32570 (working dilution 1:200)<br>ERG Abcam ab92513 (working dilution 1:200)<br>MLC-2v Synaptic Systems 310 003 (working dilution 1:200)<br>DDAH2 Elabscience E-AB-10938 (working dilution 1:200)
Validation	Antibodies were validated using a standard immunofluorescence protocol described in the manuscript, with or without antigen retrieval (using Tris pH 9.0, or sodium citrate buffer pH 6.0).

Animals and other organisms

Policy information about studies involving animals; ARRIVE guidelines recommended for reporting animal research

Laboratory animals	C57BL/6 male (Mus musculus) animals were bred in-house or obtained from Charles River Laboratories. Male African spiny mice (Acomys cahirinus) were bred in-house.
Wild animals	*Provide details on animals observed in or captured in the field; report species, sex and age where possible. Describe how animals were caught and transported and what happened to captive animals after the study (if killed, explain why and describe method; if released, say where and when) OR state that the study did not involve wild animals.*
Field-collected samples	*For laboratory work with field-collected samples, describe all relevant parameters such as housing, maintenance, temperature, photoperiod and end-of-experiment protocol OR state that the study did not involve samples collected from the field.*
Ethics oversight	All animal experiments were conducted under strict governmental and European guidelines and were approved by the Animal Welfare Committee of the Royal Netherlands Academy of Arts and Sciences, under license number AVD80100 2018 7144.

Note that full information on the approval of the study protocol must also be provided in the manuscript.
